# The Effect of N6-Methyladenosine Regulators and m6A Reader YTHDC1-Mediated N6-Methyladenosine Modification Is Involved in Oxidative Stress in Human Aortic Dissection

**DOI:** 10.1155/2023/3918393

**Published:** 2023-02-09

**Authors:** Fanxing Yin, Kun Liu, Wanfu Peng, Deying Jiang, Hao Zhang, Panpan Guo, Yinhao Wu, Xiaoxu Zhang, Chenxi Sun, Yaxuan Wang, Hecheng Wang, Yanshuo Han

**Affiliations:** ^1^School of Life and Pharmaceutical Sciences, Dalian University of Technology, Panjin, China; ^2^Department of Cardiac Surgery, Affiliated Hospital of Guizhou Medical University, Guiyang, China; ^3^Department of Vascular Surgery, Dalian Municipal Central Hospital, Dalian, China

## Abstract

Aortic dissection (AD) develops pathological changes in the separation of the true and false aortic lumen, with high lethality. m6A methylation and oxidative stress have also been shown to be involved in the onset of AD. Through bioinformatics methods, three differentially expressed m6A regulators (YTHDC1, YTHDC2, and RBM15) were excavated from the GSE52093 dataset in the Gene Expression Omnibus (GEO) database, and functional enrichment analysis of the differentially expressed genes (DEGs) regulated by m6A regulators was performed. Then, the genes with oxidative stress-related functions among these genes were found. The protein interaction network of the oxidative stress-related genes and the competing endogenous RNA- (ceRNA-) miRNA-mRNA network were constructed. Among them, DHCR24, P4HB, and PDGFRA, which have m6A differences in AD samples, were selected as key genes. We also performed immune infiltration analysis, as well as cell-gene correlation analysis, on samples from the dataset. The results showed that YTHDC1 was positively correlated with macrophage M1 and negatively correlated with macrophage M2. Finally, we extracted AD and healthy aorta RNA and protein from human tissues that were taken from AD patients and patients who received heart transplants, performed quantitative real-time PCR (qRT-PCR) on YTHDC2 and RBM15, and performed qRT-PCR and western blot (WB) detection on YTHDC1 to verify their differences in AD. The mRNA and protein levels of YTHDC1 were consistent with the results of bioinformatics analysis and were downregulated in AD. Immunofluorescence (IF) was used to colocalize YTHDC1 and endothelial cell marker CD31. After knocking down YTHDC1 in human umbilical vein endothelial cells (HUVECs), reactive oxygen species (ROS) levels had a tendency to increase and the expression of peroxide dismutase SOD2 was decreased. This study provides assistance in discovering the role of m6A regulator YTHDC1 in AD. In particular, m6A modification participates in oxidative stress and jointly affects AD.

## 1. Introduction

AD is a life-threatening medical emergency in which the inner layer of the aorta is torn or bleeding inside the aortic wall, resulting in the separation of different layers of the aortic wall; incidence rates in the general population range from 3.5 to 6 per 100,000 people [1, 2]. It is characterized by separation of the aortic parietal and subsequent creation of a false lumen that may compress the true aortic lumen. Hypertension, atherosclerosis, and some connective tissue diseases (Marfan and Ehlers-Danlos syndromes) are predisposing factors that may induce AD [3]. Its pathological process is closely related to the abnormal state of endothelial cells and smooth muscle cells and the infiltration of inflammatory cells such as macrophages into the aortic wall [4–6]. The classification of AD depends on the duration of treatment after symptom onset. Therefore, AD was acute dissection in the first two weeks, subacute after three months, and chronic after three months [7]. In patients with untreated acute aortic dissection (AAD), mortality after symptom onset increases by 1-2% per hour [8]. The current treatment for AD is surgery; although the outcome is improving, many debates about the optimal treatment remain [9]. The surgery requires a high level of skill and needs to be performed in a medical center. Patients in some underdeveloped areas are not treated in time, resulting in poor survival [10]. Prevention or treatment other than surgery is necessary. A study by Beam and Moore found that the occurrence of AD was associated with a positive family history of the patient [11]. Therefore, by identifying some key genes of AD, it may provide new ideas or methods for the treatment of AD. It is difficult to mine these genes through experiments, but it is very feasible to screen them through bioinformatics.

Adenosine methylation at the N6 position, known as m6A, is the most abundant posttranscriptional chemical modification, both in human mRNA and noncoding RNA [12]. Aberrant m6A modification levels have been implicated in various cytopathological processes such as nuclear RNA export, splicing, mRNA stability, miRNA biogenesis, and lncRNA metabolism [13]. In the study of vascular diseases, there is emerging evidence that abnormal m6A is closely related to the pathogenesis of cardiovascular diseases (CVDs) such as aortic aneurysm, vascular calcification, and pulmonary hypertension [14]. Moreover, the reduction of m6A modification level helps to inhibit the differentiation of vascular smooth muscle [15]. These are potential predisposing factors for AD, so the occurrence of AD is likely related to m6A modification. The effects of m6A on mRNA are mediated by “readers” (effectors recognizing m6A), “writers”-complex components (m6A methyltransferases), and potential “erasers” (m6A demethyltransferases) [16, 17]. Modification of m6A is performed by m6A writers, removed by m6A erasers, and recognized by reader proteins [18]. These m6A regulators are the basis and key to study m6A modification. Some m6A regulators have been found to be related to CVDs. For example, YTHDF1 can regulate pulmonary hypertension and ALKBH5 has the function of regulating the proliferation of cardiomyocytes and cardiac regeneration, as well as METTL14 leading to increased endothelial inflammation and the development of atherosclerosis [19–21]. In the aspect of AD, Zhou et al. found that the high expression of MTEEL14 and the low expression of FTO may act on some functional genes to alter their m6A levels, thereby regulating the pathogenesis of AD [22].

An imbalance between the production of ROS and the endogenous antioxidant defense system is called oxidative stress [23]. Oxidative stress has been identified as one of the underlying common causes of CVDs with vascular damage [24]. Basal concentrations of ROS are essential for the performance of cellular functions, but uncontrolled ROS concentrations can adversely affect cellular macromolecules, which can further lead to endothelial and vascular smooth muscle cell damage [25–27]. These adverse consequences of ROS cannot be avoided if endogenous antioxidants do not sufficiently function as checkpoints. Therefore, oxidative stress may also be a potential inducer of AD to a large extent. A complex relationship exists between oxidative stress-related and m6A-regulated signaling pathways, while ROS signaling has the function of regulating m6A modification [28]. Oxidative stress can upregulate or downregulate some m6A regulators to alter the degree of m6A modification and affect related molecular mechanisms [29, 30]. The molecular mechanism by which m6A and oxidative stress coact in AD is the focus of this study.

We were dedicated to discovering the underlying molecular mechanisms underlying m6A regulator-mediated m6A modification in relation to oxidative stress in AD, to provide a theoretical ground for the progress of AD etiology in the direction of the synergistic effects of m6A and oxidative stress. In this study, we first analyzed the datasets containing AD tissues and normal tissues in the GEO database, found differentially expressed m6A regulators, and constructed a network of m6A regulators and their target genes related to oxidative stress. We performed a comprehensive analysis of these genes, including enrichment analysis, ceRNA regulatory network construction, immune infiltration correlation, and drug interaction analysis. Then, we identified the differential expression of m6A regulators by quantitative real-time PCR and western blot. IF was used for colocalization. Finally, we attempted to verify the effect of YTHDC1 on ROS levels in HUVECs.

## 2. Materials and Methods

### 2.1. Data Collection and Screening of Differentially Expressed m6A Regulatory Genes

We searched the database for studies on human AD in the GEO database (https://www.ncbi.nlm.nih.gov/geo/). The mRNA expression profiling dataset GSE52093 which included AD (*n* = 5) and normal control samples (*n* = 7) was selected. Differential expression analysis of genes between AD and normal aorta was performed by using the R software package “limma.” Genes with adjusted *P* value < 0.05 and |logFC| ≥ 0.5 were identified as DEGs. Common m6A methylation regulators include ten m6A readers (HNRNPA2B1, HNRNPC, IGF2BP1, IGF2BP2, IGF2BP3, YTHDC1, YTHDC2, YTHDF1, YTHDF2, and YTHDF3), ten m6A writers (HAKAI, METTL3, METTL14, METTL16, RBM15, RBM15B, WTAP, VIRMA, ZC3H13, and ZCZHC4), and two kinds of m6A erasers (ALKBH5 and FTO). Thereafter, the m6A regulators with *P* value < 0.05 and |logFC| ≥ 0.7 were considered as differentially expressed m6A regulatory genes (DEMRGs).

### 2.2. Functional Enrichment Analysis of Differential m6A Regulatory-Related Genes

The m6A2Target database (http://m6a2target.canceromics.org) was used for prediction of the target genes of DEMRGs. The common parts of the target genes and the DEGs were differential m6A regulation-related genes. Gene Ontology (GO) enrichment analysis and Kyoto Encyclopedia of Genes and Genomes (KEGG) pathway analysis of DEMRGs were performed using the Database for Annotation, Visualization and Integrated Discovery (DAVID) (https://david.ncifcrf.gov/). GO enrichment analysis was split into the terms of biological processes (BP), cellular components (CC), and molecular functions (MF). The enrichment results with FDR < 0.05 were regarded as statistically significant.

### 2.3. The Network Construction of Exosome and Programmed Cell Death-Related Genes

Oxidative stress-related genes were downloaded from the GO database. The above genes were crossed with the differential m6A regulatory-related genes to obtain the overlapped genes. The network of association between DEMRGs and overlapping genes was constructed. The PPI network was analyzed for the overlapped genes employing the Search Tool for the Retrieval of Interacting Genes (STRING) database (https://www.string-db.org) with a combined score > 0.4. Subsequently, two networks were visualized by the Cytoscape software.

### 2.4. ceRNA Network Construction for DEMRGs

The differentially expressed long noncoding RNAs (DE-lncRNAs) in GSE52093 were screened by using the R software package “limma.” The interactions between microRNAs (miRNAs) and DEMRGs were predicted through starBase database (https://starbase.sysu.edu.cn) and miRDB database (http://mirdb.org). And the association pairs between target miRNAs and DE-lncRNAs were also predicted using starBase database. Then, the ceRNA network of mRNA-miRNA-lncRNA was constructed by using interactive miRNAs with DE-lncRNAs and DEMRGs. Cytoscape was utilized to visualize the ceRNA network of mRNA (DEMRGs)-miRNA-ceRNA (DE-lncRNA).

### 2.5. Establishment of a LASSO Model and ROC Curve Analysis

A LASSO model for the identification of DEMRGs was established by “glmnet” package. Determine the best variable in the model by the minimum lambda value. ROC monofactor analysis which could evaluate the stability and sensitivity was performed to evaluate the diagnostic value of DEMRGs in AD. The LASSO model and the ROC curve were both based on the gene expression profiles of hub genes which are differentially expressed between AD and control.

### 2.6. Identification and Analysis of Key m6A Oxidative Stress-Related Genes

The differentially methylated m6A genes between AAD and normal human aorta were obtained from the GSE147027 dataset. We found the DEGs with oxidative stress-related functions, differential m6A methylation, and regulated by DEMRGs. The GSE153434 dataset was used to verify the differential expression of these genes in different kinds of samples. These genes were defined as key genes. The “rms” package was used to establish a nomo-diagram-logistic model based on the key genes. Then, the calibration curve was established to evaluate the prediction accuracy of the nomo-diagram-logistic model. The “rmda” package was used to analyze the decision curve and clinical impact curve for assessing the nomo-diagram-logistic model. The Drug Gene Interaction Database (DGIdb) is a database of drug gene interaction data. The drugs interacting with key genes were screened by DGIdb.

### 2.7. Correlation Analysis between Key Genes and Immune Cells

CIBERSORTx is used to perform immune infiltration analysis of GSE52093 (https://cibersortx.stanford.edu). As the signature gene file, leukocyte signature matrix (LM22) can distinguish 22 human hematopoietic cell phenotypes. The proportion of immune cells in each sample was obtained, and further, the relationship between immune cells and genes which included DEMRGs and key genes was analyzed. The relationship of DEMRGs and key genes was also analyzed based on the expression levels in each sample.

### 2.8. Human Aortic Samples

All protocols using human specimens were approved by the Human Research Ethics Committees of the Affiliated Hospital of Guizhou Medical University (Guiyang, China), with approval number 2022-LS018. Aortic tissue was obtained from AD patients and patients receiving heart transplantation (controls), and informed consent was obtained from patients or their family members. All samples were stored in liquid nitrogen or paraformaldehyde as soon as possible after surgical resection to avoid sample spoilage.

### 2.9. The mRNA Expression Levels of DEMRGs in AD Samples

qRT-PCR was conducted to validate part of m6A methylation regulators in tissue samples. Grind human AD tissue and normal aortic tissue in liquid nitrogen to extract total RNA. Then, total RNA was reverse transcribed to cDNA using PrimeScript™ RT Master Mix (RR036A, TaKaRa Bio, Shiga, Japan), and qRT-PCR was performed using the TB Green® Premix Ex Taq™ II (RR820A, TaKaRa Bio, Shiga, Japan). GAPDH was used as the reference gene, and the relative expression levels of mRNAs in tissues were calculated with the 2−∆∆CT method.

### 2.10. The Protein Expression Levels of YTHDC1 in AD Tissues

Protein samples from AD and normal tissues were added to sodium dodecyl sulfate-polyacrylamide gel (15 *μ*g sample per gel lane) to separate, and then, the protein bands were transferred to polyvinylidene fluoride membranes. The membranes were blocked, followed by incubation with rabbit anti-YTHDC1 (abs117811, Absin, Shanghai, China) and rabbit anti-GAPDH (D110016, Sangon Biotech, Shanghai, China). The blots were then incubated with goat anti-rabbit IgG which was labeled by horseradish peroxidase. Finally, the density of immunoreactive bands normalized to the signal intensity of GAPDH was determined using the Electro Chemical Luminescence Kit and ImageJ software 1.53.

### 2.11. IF Colocalization of YTHDC1 and CD31 in Human AD Tissues

IF staining analysis of YTHDC1 and endothelial cell marker CD31 was carried out for cellular localization in AD tissue. AD tissue sections were passed through xylene, isopropanol, and ethanol for dewaxing and rehydrating. Dehydrated sections were placed in citrate buffer under high temperature and pressure for antigen retrieval. Then, add hydrogen peroxide to block endogenous peroxidase. After incubation, AD sections were blocked by Immunol Staining Blocking Buffer (P0102, Beyotime, Shanghai, China). Incubate tissue with rabbit anti-YTHDC1 antibody (abs117811, Absin, Shanghai, China) and the Cy3 (red)-conjugated goat anti-rabbit IgG (GB21303, Servicebio, Wuhan, China). Then, rabbit anti-CD31 antibody (abs120102, Absin, Shanghai, China) and the 488 (green)-conjugated goat anti-rabbit IgG (abs20025, Absin, Shanghai, China) were used to incubate the tissue sequentially. Finally, the sections were stained by Antifade Mounting Medium with DAPI (P0131, Beyotime, Shanghai, China). The prepared sections can be viewed under a fluorescence microscope.

### 2.12. Endothelial Cell Culture and YTHDC1 siRNA Transfection

HUVECs were cultured with complete growth medium DMEM High Glucose (MA0545, Meilunbio, Dalian, China), 10% fetal bovine serum (10100-147, Gibco, Thermo Fisher Scientific, USA), and 1% penicillin/streptomycin (10000 U/mL; SV30010, HyClone, USA) at 37°C in moist air with 5% CO_2_. YTHDC1 siRNA and control (NC) siRNA were from RiboBio (Guangzhou, China). HUVECs were seeded into 6-well plates or 24-well plates at a concentration of 30%. After cell spreading, HUVECs were transfected with 75 nM YTHDC1 siRNAs or NC siRNAs for 48 h by riboFECT™ CP Transfection Kit (C10511-05, RiboBio, Guangzhou, China). ROS detection kit (WLA131a, Wanleibio, Shanghai, China) was used to detect ROS levels of HUVECs in the si-YTHDC1 and si-NC groups under a luciferase microplate reader. The excitation wavelength was 488 nm, and the emission wavelength at 525 nm had the maximum wave peak, and the intensity was proportional to the intracellular ROS levels. As a superoxide dismutase, SOD2 inhibits ROS levels and interacts with YTHDC1; we further examined the difference of SOD2 RNA expression between human AD tissues and normal aortic tissues. Then, total RNA was extracted after siRNA transfection, for further qRT-PCR analysis of transfection efficiency as well as RNA levels of SOD2 and pattern recognition receptors (PRRs) such as NOD1, NOD2, and NLRP3.

## 3. Results

### 3.1. Identification of Differentially Expressed m6A Regulatory Genes

**Figure 1:**
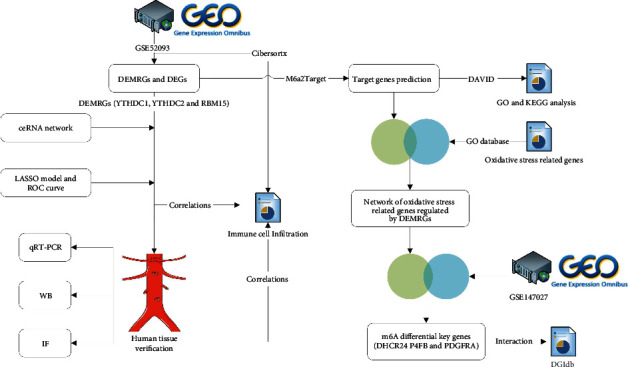
Study workflow.

**Figure 2:**
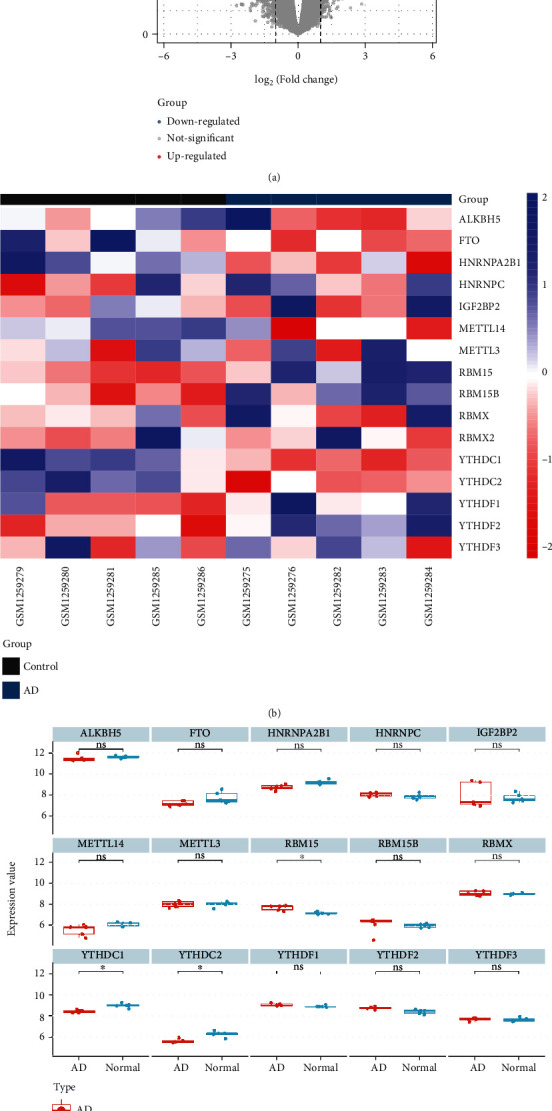
Identification of DEMRGs and DEGs. (a) Volcano plot of DEGs in GSE52093. (b) Heat map of differential expression of m6A regulators. (c) Expression boxplot of m6A regulators. “^∗^” means adj. *P* value < 0.05.

The main workflow of this research is summarized in Figure 1. To obtain DEGs related to AD, we compared them with healthy controls. 1807 DEGs were obtained in GSE52093 with criteria for adjusted *P* value < 0.05 and |logFC| ≥ 0.7 (Figure 2(a) and Supplementary File 1). And the expression of m6A regulatory genes is shown in Figures 2(b) and 2(c). Through the screening of differential expression, we finally obtained three m6A regulators (YTHDC1, YTHDC2, and RBM15) as DEMRGs. Compared with normal samples, YTHDC1 and YTHDC2 were upregulated in AD tissues, whereas RBM15 was downregulated in contrast.

### 3.2. Functional Enrichment of Differential m6A Regulatory-Related Genes

**Figure 3:**
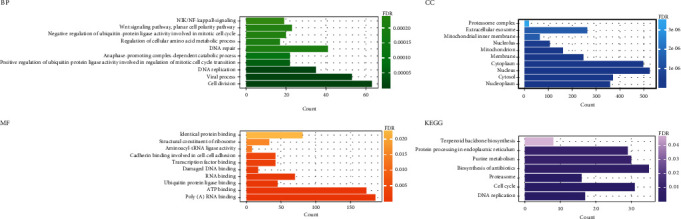
GO function and KEGG pathway enrichment results of DEGs regulated by DEMRGs.

Through the m6A2Target database, we obtained a total of 7685 target genes that may be modified by DEMRGs. The target genes of YTHDC1, YTHDC2, and RBM15 methylation modification had 5547, 2724, and 2702, respectively. The intersection genes of these target genes and DEGs were differential m6A regulatory-related genes in AD. Then, 1306 m6A regulatory-related genes were used to perform GO and KEGG enrichment analyses. 87 GO terms and 7 KEGG pathways with FDR < 0.05 were obtained. GO enrichment analysis revealed that differential m6A regulatory-related genes were mainly enriched in cell division, viral process, nucleoplasm, cytosol, poly(A) RNA binding, ATP binding, etc. Overall, more functions were enriched in the translation process and cell division stage. In the KEGG pathway, differential m6A regulatory-related genes were enriched in DNA replication, cell cycle, proteasome biosynthesis of antibiotics, purine metabolism, protein processing in endoplasmic reticulum, and terpenoid backbone biosynthesis (Figure 3 and Table 1).

### 3.3. Association Network of Exosome and Programmed Cell Death-Related Genes

**Figure 4:**
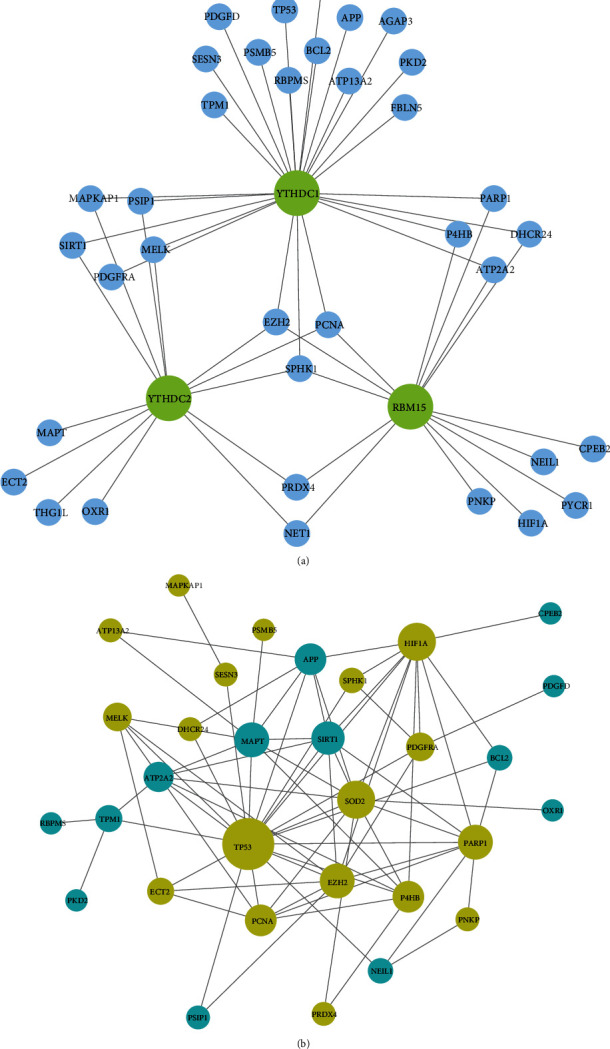
The network of oxidative stress-related genes regulated by DEMRGs. (a) Network of the relationship between DEMRGs and oxidative stress-related genes. (b) PPI network of oxidative stress-related genes: genes in yellow were upregulated, and genes in blue were downregulated.

A total of 418 oxidative stress-related genes were obtained from the GO database. Then, we got the oxidative stress-related genes among the differential m6A regulatory-related genes in AD. An interaction network was constructed to elucidate the association of DEMRGs with oxidative stress (Figure 4(a)). SOD2 was interacted with YTHDC1. Then, the PPI network of these genes was also constructed to further show the association of oxidative stress (Figure 4(b)).

### 3.4. ceRNA Network Construction for DEMRGs

**Figure 5:**
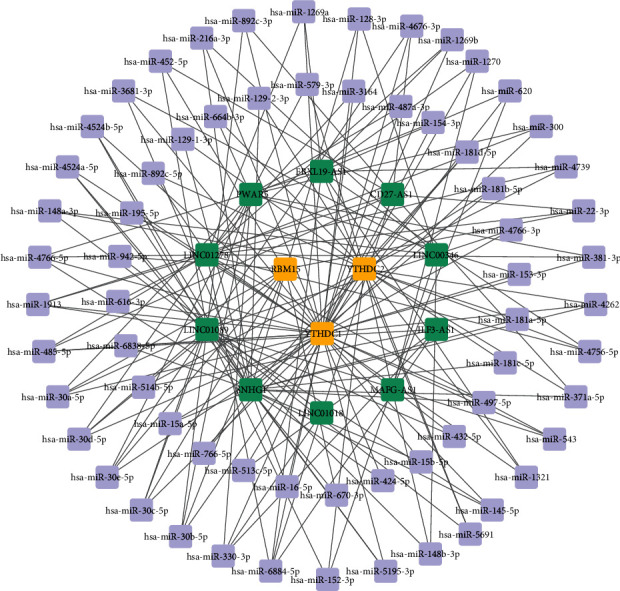
The ceRNA network of mRNA-miRNA-lncRNA for DEMRGs: yellow stands for DEMRGs, purple stands for miRNAs, and green stands for ceRNAs (lncRNAs).

After differential expression analysis, 16 DE-lncRNAs were screened. lncRNA-miRNA interaction pairs were predicted by the starBase database. Then, we selected the DEMRG target miRNA predicted by starBase database and miRDB database jointly. The ceRNA network of mRNA-miRNA-lncRNA for AD-related m6A regulators was constructed with 3 m6A regulators, 65 miRNAs, and 10 lncRNAs (42 miRNAs for YTHDC1, 21 miRNAs for YTHDC2, and 8 miRNAs for RBM15) (Figure 5).

### 3.5. Establishment of the LASSO Model and Assessment of the ROC Curve

**Figure 6:**
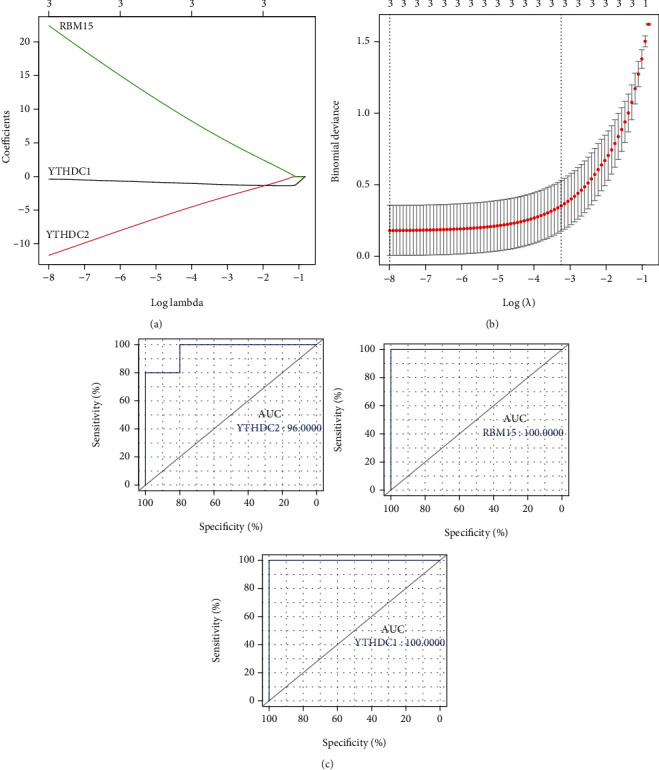
Identification of DEMRGs. (a) Coefficient plot of LASSO regression. (b) Cross verification curve of LASSO regression. (c) ROC curve analysis of DEMRGs.

The gene signature of YTHDC1, YTHDC2, and RBM15 was all determined by LASSO regression analysis (Figures 6(a) and 6(b)). Then, the ROC curve was used to determine the accuracy of the LASSO model by the AUC value. Consequently, the diagnostic accuracy (AUC) of YTHDC1, YTHDC2, and RBM15 for AD was 100, 96, and 100, respectively, in the GSE52093 dataset (Figure 6(c)).

### 3.6. Identification and Analysis of Key m6A Oxidative Stress-Related Genes

**Figure 7:**
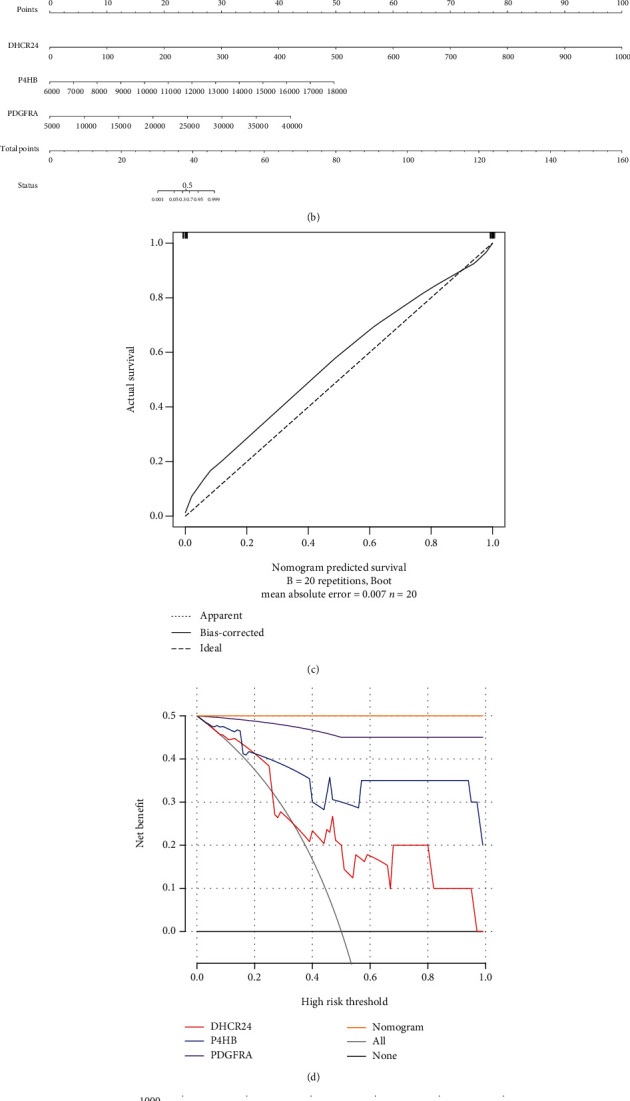
Identification of key m6A oxidative stress-related genes. (a) Boxplot of expression of key m6A oxidative stress-related genes in GSE153434: “^∗^” means *P* < 0.05, “^∗∗∗^” means *P* < 0.001, and “^∗∗∗∗^” means *P* < 0.0001. (b) Nomo-diagram-logistic model. (c) Calibration curve analysis. (d) Decision curve analysis. (e) Clinical impact analysis.

**Figure 8:**
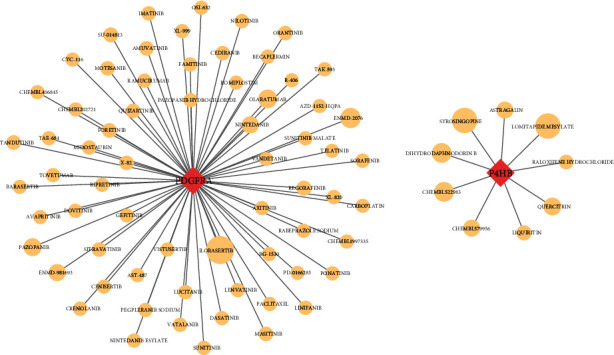
Interaction of drugs with P4HB and PDGFRA: the size of the shape represents the degree of interaction.

A total of six differentially m6A methylated DEGs regulated by m6A regulators were involved in oxidative stress function. Three of the genes also had the same trend of expression differences in GSE153434, namely, DHCR24, P4HB, and PDGFRA (Figure 7(a)). Three key genes were all upregulated in AD. The calibration curve indicated that the error between the actual AD risk and the predicted risk is very small, suggesting that the nomo-diagram-logistic model owns high accuracy to predict AD (Figures 7(b) and 7(c)). Decision curve analysis indicated that the nomo-diagram-logistic curve was higher than the gray line, “DHCR24” curve, “P4HB” curve, and “PDGFRA” curve (Figures 7(d) and 7(e)). Drugs that interact with P4HB and PDGFRA were found in DGIdb (Figure 8).

### 3.7. Correlations between DEMRGs and Immune Cells

**Figure 9:**
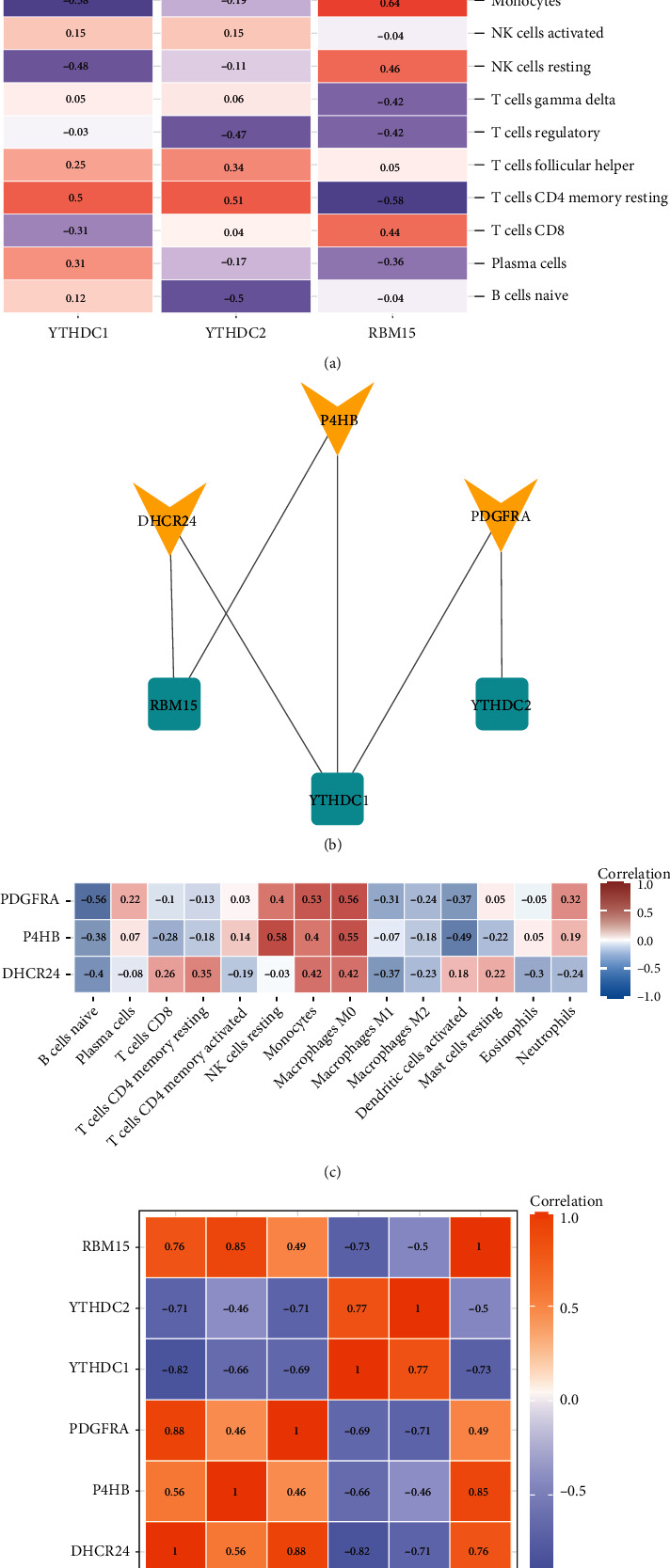
Correlations between genes and immune cells. (a) Correlation between DEMRGs and immune cells. (b) The correlation between key genes and immune cells. (c) The regulatory relationship between DEMRGs and key genes. (d) Correlation between DEMRGs and key genes.

The CIBERSORTx database is based on the expression matrix of GSE52093 and LM22 to analyze immune cell infiltration. The percentage of immune cells in each sample was evaluated (Supplementary File 2). According to the expression of DEMRGs and immune cell content in each sample, the correlation of DEMRGs and key genes with immune cells was calculated by the Pearson correlation analysis (Figures 9(a) and 9(b)). DHCR24 and P4HB were regulated by YTHDC1 and RBM15, and PDGFRA was regulated by YTHDC1 and YTHDC2 (Figure 9(c)). Then, the Pearson correlation between DEMRGs and key genes was also analyzed (Figure 9(d)).

### 3.8. Validation of DEMRGs in Human Aortic Tissue Level

**Figure 10:**
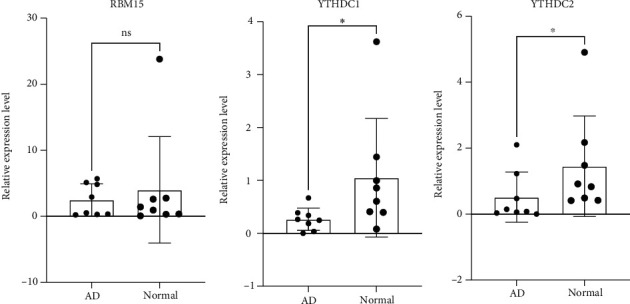
Human aortic tissue validation, qRT-PCR results of DEMRGs: “^∗^” means *P* < 0.05.

**Figure 11:**
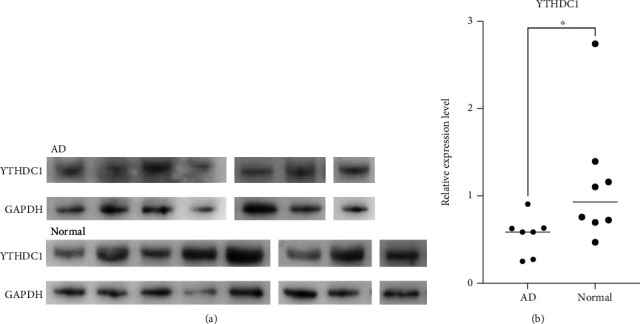
Human aortic tissue validation, WB results of YTHDC1. (a) Western blot in AD and normal. (b) Significance test of difference: “^∗^” means *P* < 0.05.

**Figure 12:**
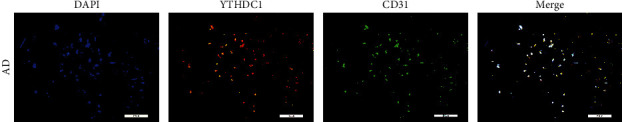
IF staining of DAPI, YTHDC1, and CD31 and their merged images.

We performed qRT-PCR to detect the mRNA expression of YTHDC1, YTHDC2, and RBM15 in human aorta specimens. The primer sequences are shown in Table 2. YTHDC1 and YTHDC2 mRNA was confirmed to be significantly downregulated in AD samples in comparison with control aortas. The *P* value of Mann–Whitney test of YTHDC1 and YTHDC2 was 0.0148 and 0.0499, respectively (Figure 10). We also detected the mRNA levels of other m6A regulators (YTHDF1, YTHDF2, YTHDF3, RRP8, ALKBF1, and ALKBF3) in aortic tissue by qRT-PCR (Supplementary File 3). We selectively performed western blot analysis for YTHDC1 in AD and normal aorta tissues. The blot images of YTHDC1 and its relative expression levels measured were displayed through western blotting in all samples (Figures 11(a) and 11(b)). The results of IF staining of paraffin sections of AD tissue showed that the fluorescent double staining of YTHDC1 and CD31 had obvious colocalization and often coexpressed in the same cell (Figure 12). At the same time, we noticed that YTHDC1 was expressed in the intima, media, and adventitia of AD, and CD31 was more expressed in the intima.

### 3.9. Effect of YTHDC1 Knockdown on ROS Levels and Inflammation in HUVEC

**Figure 13:**
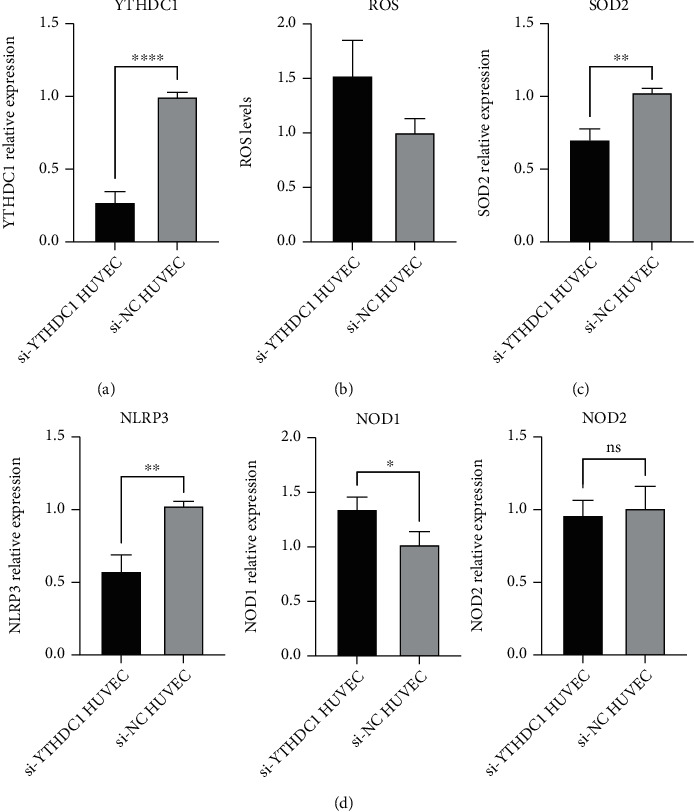
Effects of YTHDC1 on ROS and inflammation. “^∗^” means *P* < 0.05, “^∗∗^” means *P* < 0.01, and “^∗∗∗∗^” means *P* < 0.0001. (a) YTHDC1 knockdown efficiency. (b) ROS levels of the si-YThDC1 groups and si-NC groups. (c) SOD2 relative expression of the si-YTHDC1 groups and si-NC groups. (d) PRR (NOD1, NOD2, and NLRP3) relative expression of the si-YTHDC1 groups and si-NC groups.

The average knockdown efficiency of YTHDC1 detected by qRT-PCR was 73% (Figure 13(a)). Compared with that of the si-NC group, the signal intensity of the si-YTHDC1 group had a tendency to increase (Figure 13(b)). The decreased expression of YTHDC1 may lead to the increase of ROS. In qRT-PCR of human tissues, SOD2 expression was downregulated in AD (Supplementary File 4). Then, we found that the mRNA expression quantity of peroxide dismutase SOD2 was significantly reduced in the si-YTHDC1 groups compared with the si-NC groups (Figure 13(c)). In the same way, NOD2 expression was significantly increased after YTHDC1 knockdown, but NLPR3 moved towards low expression (Figure 13(d)).

## 4. Discussion

Although many studies have demonstrated that m6A methylation and oxidative stress play an important role in the occurrence of CVDs, the molecular mechanism of their joint effect on AD is rarely reported. Based on AD samples and normal aortic samples, we used bioinformatics and molecular biology experimental methods to study the potential molecular mechanism of m6A and oxidative stress in AD. Three DEMRGs in AD tissue and normal aortic tissue were analyzed by the GSE52093 dataset. YTHDC1 and YTHDC2 were downregulated in AD, while RBM15 was upregulated. Subsequent ROC analysis also showed that YTHDC1, YTHDC2, and RBM15 have good diagnostic capabilities. We found DEGs regulated by three DEMRGs, and then, the function and pathway enrichment of these genes were analyzed. Cell division and viral process in biological process were more prominent. The cellular components in which they reside were abundantly enriched in the nucleoplasm, nucleus, and cytoplasm. Molecular functions mainly included poly(A) RNA binding and ATP binding. KEGG enrichment results showed that these genes were involved in the periodic division of cells. According to the DE-lncRNAs and DEMRGs in GSE52093 to predict miRNAs, the ceRNA-miRNA-mRNA relationship chain was obtained, and the ceRNA network was constructed. The oxidative stress-related genes were obtained from the GO database, and DEGs regulated by DEMRGs with oxidative stress-related functions were obtained through Venn diagram analysis. Then, the protein interaction network of these genes was constructed, and the upregulated genes with high degree values such as TP53 and SOD2 were found. Three of these genes were significantly different in the degree of m6A methylation in AD and normal tissues, namely, DHCR24, P4HB, and PDGFRA, and they were significantly upregulated in two different datasets. In the gene-cell correlation analysis, both YTHDC1 and RBM15 were strongly associated with resting and activated mast cells, and DHCR24, P4HB, and PDGFRA were generally associated with macrophage M0. Finally, we found that the expression of YTHDC1 was obvious in endothelial cells, and the decreased expression of YTHDC1 had an effect on ROS.

The molecular mechanism of m6A regulators is the core of the scientific question of this study. YTHDC1, whose full name is YTH domain containing 1, is a type of m6A “reader” protein. Roundtree et al. found that YTHDC1 mediates nuclear-to-cytoplasmic transport of m6A methylated mRNAs. They knocked down YTHDC1, which resulted in boosted signal intensity around the nuclear region and decreased in the cytoplasmic region [31]. This suggests that YTHDC1 plays an active role in nuclear mRNA export. They also found that overexpression of YTHDC1 reduced m6A levels of nuclear mRNA, whereas our study found that YTHDC1 was underexpressed in AD, in line with the conclusion that m6A levels are elevated in AD. Gao et al. demonstrated in a mouse model that Ythdc1 knockout resulted in left ventricular enlargement and systolic dysfunction, as well as decreased cardiomyocyte contractility and disordered sarcomere arrangement at the cellular level [32]. YTHDC1 affects mRNA splicing through recruitment and regulation of pre-mRNA splicing factors, which may be the underlying cause of aberrant splicing of titin in Ythdc1-deficient cardiomyocytes and thus aberrant sarcomere contraction [33]. m6A modification of Ythdc1 directs promoter-proximal RNA polymerase II to suspend work, which is also critical for precise control of gene expression [34]. YTHDC2 contains multiple domains that can bind to RNAs with different nucleotide preferences, and when YTHDC2 binds to RNA helicase, m6A modification of the coding region can promote the translation of structured mRNA [35, 36]. In the YTH-binding domains of YTHDC1 and YTHDC2, a1, a2, and the coils preceding a1, a2, and b4 could constitute the m6A-binding pocket that can recognize cavity-inserted methyl groups, and the m6A-binding surface is highly conserved in evolution, compared to other YTH domains [37, 38]. This is an important molecular mechanism of YTH domain binding to m6A nucleotides. As a special RNA-binding protein, m6A methyltransferase RBM15 plays an important role in cell growth and apoptosis by regulating various signaling pathways such as Notch and Wnt [39]. However, the role of RBM15 in CVDs was rarely reported, and only a few studies linked it to some blood disorders.

GO-BP results showed that these genes were extensively involved in cell cycle-related processes, as well as Wnt signaling pathway and planar cell polarity pathway. A bioinformatics study found that cell cycle-related genes were significantly upregulated in thoracic aortic aneurysm and dissection tissues in a mouse model, and they were essential for cell proliferation [40]. In response to changes in the local vascular environment, vascular smooth muscle cells proliferate to alter the differentiation/contraction phenotype [41]. These genes might play a role in VSMC proliferation and phenotypic changes. The Wnt signaling pathway may be involved in ventricular hypertrophy, and the planar cell polarity pathway has the function of regulating the morphology of polarization, helping the development of the poles of the arteries and veins of the heart [42, 43]. As indicated by GO-CC, these DEGs are mainly enriched around the nucleoplasm or nucleus. This is strongly associated with nuclear mRNA export of YTHDC1. These DEGs were also enriched for molecular functions related to transcription, such as damaged DNA, ATP, transcription factor, poly(A) RNA, and RNA binding. KEGG analysis also showed their high correlation with cell cycle.

In our study, we found three oxidative stress-related DEGs with m6A differences and regulated by DEMRGs, namely, DHCR24, P4HB, and PDGFRA. DHCR24 plays a vital role in lipid metabolism and is an enzyme that catalyzes conversion of the bioactive metabolite desmosterol to cholesterol [44]. Desmosterol is an endogenous agonist of the liver X receptor which plays an important role in the regulation of lipid metabolism and is involved in atherosclerosis and inflammation. Inhibition of DHCR24 leads to accumulation of desmosterol, and depletion of desmosterol in myeloid cells by overexpression of DHCR24 promotes the progression of atherosclerosis [45, 46]. DHCR24 is also one of the host markers of oxidative stress, and it has been reported that the expression of DHCR24 was upregulated in the acute response to oxidative stress and, in contrast, downregulated in the chronic response [47, 48]. High expression of DHCR24 gene is associated with apoptosis induced by antioxidative stress [49]. P4HB protein is an endoplasmic reticulum molecular chaperone that contributes to the maintenance of endoplasmic reticulum protein homeostasis [50]. PDIA1 encoded by it participates in vascular redox cell signaling. The expression of P4HB splice variants was elevated in smooth muscle cells, especially isoforms P4HB-02 and P4HB-021 [51]. Increased P4HB expression was also produced in the serum of the Kawasaki disease causing systemic vasculitis [52]. A possible effect of P4hb in regulating oxidative stress in middle cerebral ischemia/reperfusion has been reported in mouse models [53]. PDGFRA has a relevant functional role in vascular remodeling; its expression can characterize aortic-derived telocytes, and telocyte-mediated regulation of aortic remodeling may promote aortic repair and prevent adverse remodeling [54, 55]. In mouse model studies, it was demonstrated that PDGFRA cell populations were functionally capable of promoting differentiation into cardiomyocytes, and silencing of PDGFRA suppressed this ability [56]. We found that PDGFRA was highly correlated with ILORASERTIB in drug interactions. ILORASERTIB is an ATP-competitive, multitargeted kinase inhibitor that inhibits cellular autophosphorylation [57]. cGMP-dependent protein kinase 1 (PKG1) can help regulate vascular tone and smooth muscle cell phenotype, and cGMP-PKG1 increases the degree of autophosphorylation in PRKG1 gene mutations that cause thoracic AD [58].

In the analysis of the correlation between DEMRGs and immune cells, we found that YTHDC1 was positively correlated with macrophage M1 and negatively correlated with macrophage M2, and it was also positively correlated with resting mast cells and negatively correlated with activated mast cells. The presence of aortic inflammatory cell infiltration and elastic fiber destruction in AD undoubtedly highlights the involvement of macrophages [59]. M1 macrophages can promote inflammation, while M2 macrophages can eliminate inflammation [60]. Macrophages are central to aortic wall inflammation, and angiotensin II regulates macrophages leading to AD development. Thrombospondin 1, which can be induced by angiotensin II, is markedly elevated in AD patients and may be involved in AD by promoting M1 macrophage planning and vascular smooth muscle apoptosis [61, 62]. M2 macrophages act as a protective factor with phenotype repair cell function in the study of blood blister-like aneurysms [63]. Macrophages may not only participate in the onset of AD but also in the repair of AD. m6A methylation modifies and facilitates macrophage pyroptosis and inflammation in atherosclerosis [64]. Another study found that important m6A “writers”-METTL3 promote M1 macrophage programming through m6A methylation [65]. Therefore, whether YTHDC1 can induce the polarization of M0 macrophages is a question worthy of our further study.

Elevated ROS levels are a feature of most vascular diseases, and their occurrence in endothelial cells has been found to contribute to susceptibility to aortic dissection [66]. As a manganese-containing superoxide dismutase located in mitochondria, SOD2 scavenge ROS to protect cells from oxidative stress [67]. Our conclusion found that the downregulation of YTHDC1 may cause the increase of ROS level and the decrease of SOD2 expression, which was consistent with the above molecular mechanism. Therefore, the decreased expression of YTHDC1 may disrupt the homeostasis of endothelial cells. However, the specific mechanism of ROS elevation and the influence of other factors on ROS in AD need to be further studied.

This study is aimed at providing help in discovering the role of m6A regulators in AD, but our findings still need more in vivo experiments to confirm and further research to explore deeper molecular mechanisms.

## 5. Conclusions

YTHDC1, YTHDC2, and RBM15, as m6A regulators, had significant differences in expression in AD. m6A methylation and oxidative stress mediated by these three factors may be associated with the onset of AD. Among them, YTHDC1 was also found to be related to the polarization of macrophages and the activation of mast cells in correlation with immune cells. We also verified the significant downregulation of YTHDC1 and YTHDC2 in subsequent human tissues including AD tissue and healthy aortic tissue. In addition, we found three oxidative stress-related DEGs with differential m6A modification, namely, DHCR24, P4HB, and PDGFRA. The expression of YTHDC1 in endothelial cells was verified by IF, and the ROS level increased after the expression of YTHDC1 decreased, while the expression of SOD2 decreased. These findings may further advance the discovery of the molecular mechanisms of AD, helping to aid subsequent research and the development of treatments.

## Figures and Tables

**Table 1:** GO function and KEGG pathway enrichment results of DEGs regulated by DEMRGs.

ID	Description	Count	FDR
BP			
GO:0051301	Cell division	63	1.29*E* − 07
GO:0016032	Viral process	53	4.91*E* − 06
GO:0006260	DNA replication	35	5.03*E* − 06
GO:0051437	Positive regulation of ubiquitin-protein ligase activity involved in the regulation of mitotic cell cycle transition	22	5.45*E* − 05
GO:0031145	Anaphase-promoting complex-dependent catabolic process	22	9.07*E* − 05
GO:0006281	DNA repair	41	1.75*E* − 04
GO:0006521	Regulation of cellular amino acid metabolic process	17	1.75*E* − 04
GO:0051436	Negative regulation of ubiquitin-protein ligase activity involved in mitotic cell cycle	20	1.91*E* − 04
GO:0060071	Wnt signaling pathway, planar cell polarity pathway	23	1.91*E* − 04
GO:0038061	NIK/NF-kappaB signaling	19	2.38*E* − 04

CC			
GO:0005654	Nucleoplasm	359	2.27*E* − 34
GO:0005829	Cytosol	371	3.79*E* − 23
GO:0005634	Nucleus	525	1.40*E* − 20
GO:0005737	Cytoplasm	498	1.46*E* − 17
GO:0016020	Membrane	247	3.51*E* − 14
GO:0005739	Mitochondrion	161	4.36*E* − 11
GO:0005730	Nucleolus	106	2.07*E* − 07
GO:0005743	Mitochondrial inner membrane	64	1.35*E* − 06
GO:0070062	Extracellular exosome	263	1.35*E* − 06
GO:0000502	Proteasome complex	19	3.52*E* − 06

MF			
GO:0044822	Poly(A) RNA binding	186	4.37*E* − 25
GO:0005524	ATP binding	173	7.19*E* − 08
GO:0031625	Ubiquitin protein ligase binding	45	4.40*E* − 04
GO:0003723	RNA binding	70	7.82*E* − 04
GO:0003684	Damaged DNA binding	17	0.001372238
GO:0008134	Transcription factor binding	42	0.002543152
GO:0098641	Cadherin binding involved in cell-cell adhesion	42	0.003669701
GO:0004812	Aminoacyl-tRNA ligase activity	8	0.01359803
GO:0003735	Structural constituent of ribosome	33	0.015456237
GO:0042802	Identical protein binding	81	0.022626324

KEGG			
hsa03030	DNA replication	17	1.13*E* − 06
hsa04110	Cell cycle	31	3.92*E* − 06
hsa03050	Proteasome	16	7.54*E* − 05
hsa01130	Biosynthesis of antibiotics	35	0.005013539
hsa00230	Purine metabolism	30	0.008102754
hsa04141	Protein processing in the endoplasmic reticulum	29	0.008102754
hsa00900	Terpenoid backbone biosynthesis	8	0.046861012

**Table 2:** qRT-PCR primer sequences.

Gene	Primer	Sequence (5′ to 3′)
YTHDC1	Forward primer	TCT TCC GTT CGT GCT GTC C
	Reverse primer	GGA CCA TAC ACC CTT CGC TT

YTHDC2	Forward primer	GAG AAT TGG GCT GTC GTT AAA G
	Reverse primer	TGA AGC AGG ATG AAA TCG TAC T

RBM15	Forward primer	CCT TGT GAG TTC TCC CAG CAG TTC
	Reverse primer	GGA CGC ACC ACG GAC AAT GAT C

YTHDF1	Forward primer	TGG ACA CCC AGA GAA CAA AAG G
	Reverse primer	TGA GGT ATG GAA TCG GAG GGT

YTHDF2	Forward primer	AGT GTC AGG GAC AAA AGC CTC C
	Reverse primer	TTT TGG TCT CTG CTC CAA GAG G

YTHDF3	Forward primer	TAG GGA GTC TGT CCG CCA TT
	Reverse primer	GAC ATT CTT CAC CGC AAC CC

RRP8	Forward primer	CAG TGG TAA GAG GTT GCT CCA T
	Reverse primer	TGG TAT GCT CTT CCC TCT GC

ALKBH3	Forward primer	AGA GAA CCG AGA GTC AAC CTG ACC
	Reverse primer	CTA GCA GCA CCA GCC TCT TGA AG

ALKBH1	Forward primer	TCA GCA GAT CAT TAC ACA CCT T
	Reverse primer	CTA GCT CAG ATC TGT CTA CGT G

## Data Availability

The datasets used or analyzed during the current study are available from the corresponding author on reasonable request. The names of the repository/repositories and accession number(s) can be found in the article/Supplementary Material.

## Supplementary Materials

Supplementary Materials Supplementary material 1: differential expression analysis of genes in GSE52093. Supplementary material 2: immune infiltration analysis of GSE52093. Supplementary material 3: qRT-PCR results of additional m6A regulators (YTHDF1, YTHDF2, YTHDF3, RRP8, ALKBF1, and ALKBF3) in human aortic dissection tissue and healthy aortic tissue, “^∗^” represents *P* < 0.05; “^∗∗∗^” represents *P* < 0.001. Supplementary material 4: the mRNA expression of SOD2 in human AD samples and normal samples.
