# Supplementary material for: The Effect of N6-Methyladenosine Regulators and m6A Reader YTHDC1-Mediated N6-Methyladenosine Modification Is Involved in Oxidative Stress in Human Aortic Dissection
Source: Oxid Med Cell Longev. 2023 Feb 9;2023:3918393. doi: 10.1155/2023/3918393 (PMC9935809; doi:10.1155/2023/3918393)
Supplement: Supplementary Materials — Supplementary material 1: differential expression analysis of genes in GSE52093. Supplementary material 2: immune infiltration analysis of GSE52093. Supplementary material 3: qRT-PCR results of additional m6A regulators (YTHDF1, YTHDF2, YTHDF3, RRP8, ALKBF1, and ALKBF3) in human aortic dissection tissue and healthy aortic tissue, “∗” represents P < 0.05; “∗∗∗” represents P < 0.001. Supplementary material 4: the mRNA expression of SOD2 in human AD samples and normal samples. [file 3918393.f1.zip › Supplementary File 2.pdf]

CIBERSORTx Output Display

Input Sample	B cells naive	B cells memory	Plasma cells	T cells CD8	T cells CD4 naive	T cells CD4 memory resting	T cells CD4 memory activated	T cells follicular helper	T cells regulatory (Tregs)	T cells gamma delta	NK cells resting	NK cells activated	Monocytes	Macrophages M0	Macrophages M1	Macrophages M2	Dendritic cells resting	Dendritic cells activated	Mast cells resting	Mast cells activated	Eosinophils	Neutrophils
GSM1259279	0.17	0	0.124	0	0	0.141	0	0.056	0	0	0	0.025	0	0.014	0.093	0.238	0	0	0.138	0	0	0
GSM1259280	0.102	0.042	0	0.034	0	0.173	0	0.111	0	0	0	0.069	0	0.098	0.049	0.243	0	0	0.078	0	0	0
GSM1259281	0.102	0	0.172	0.055	0	0.113	0	0.02	0.043	0	0	0.048	0	0	0.085	0.218	0.048	0	0.097	0	0	0
GSM1259285	0.074	0	0.18	0.002	0	0.185	0	0.075	0	0.028	0	0.063	0	0	0.051	0.258	0.057	0	0.026	0	0	0
GSM1259286	0.121	0	0.078	0	0	0.193	0	0	0.047	0.085	0	0	0.023	0.078	0.041	0.213	0	0	0.123	0	0	0
GSM1259275	0.237	0	0.19	0	0	0.109	0	0.056	0.05	0	0.001	0.037	0	0.062	0.061	0.153	0	0	0.045	0	0	0
GSM1259276	0.067	0	0.011	0.076	0	0.124	0	0.037	0	0	0.039	0.004	0.09	0.077	0.085	0.279	0	0	0	0.087	0	0.023
GSM1259282	0.087	0	0.076	0.038	0	0.084	0	0.011	0.04	0	0.014	0.047	0.025	0.218	0	0.257	0	0.075	0.014	0	0	0.013
GSM1259283	0.126	0	0	0	0.027	0.007	0	0.037	0	0	0	0.024	0	0	0.024	0.605	0.065	0	0	0.083	0	0.002
GSM1259284	0.12	0	0.196	0.098	0	0.156	0	0.094	0	0	0	0.099	0.084	0	0.058	0.088	0	0	0	0	0	0.007
