# Supplementary figures and images for: The Effect of N6-Methyladenosine Regulators and m6A Reader YTHDC1-Mediated N6-Methyladenosine Modification Is Involved in Oxidative Stress in Human Aortic Dissection
Source: Oxid Med Cell Longev. 2023 Feb 9;2023:3918393. doi: 10.1155/2023/3918393 (PMC9935809; doi:10.1155/2023/3918393)

# ALKBF1

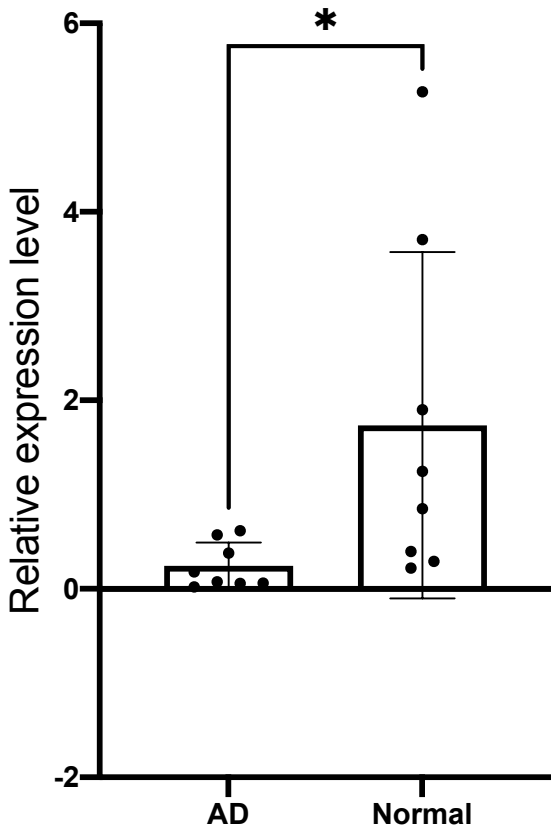

Supplement: Supplementary Materials — Supplementary material 1: differential expression analysis of genes in GSE52093. Supplementary material 2: immune infiltration analysis of GSE52093. Supplementary material 3: qRT-PCR results of additional m6A regulators (YTHDF1, YTHDF2, YTHDF3, RRP8, ALKBF1, and ALKBF3) in human aortic dissection tissue and healthy aortic tissue, “∗” represents P < 0.05; “∗∗∗” represents P < 0.001. Supplementary material 4: the mRNA expression of SOD2 in human AD samples and normal samples. [file 3918393.f1.zip › Supplementary File 3-ALKBF1.pdf]

# ALKBF3

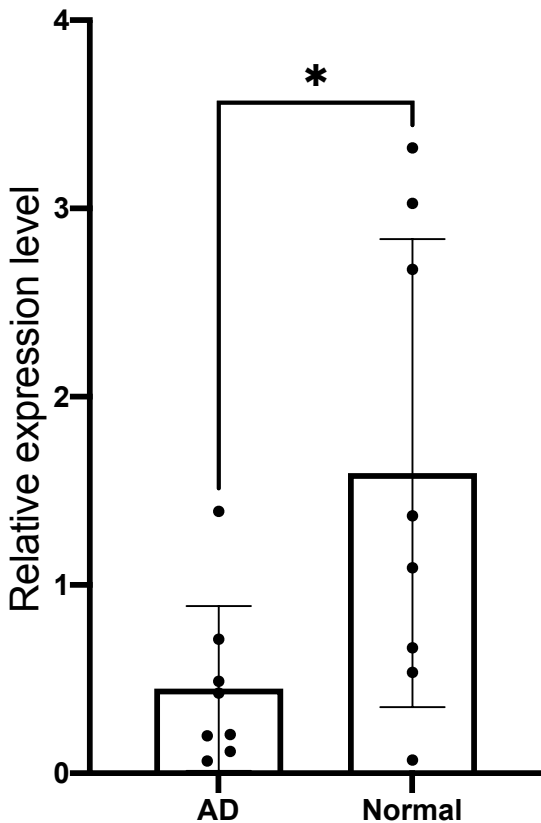

Supplement: Supplementary Materials — Supplementary material 1: differential expression analysis of genes in GSE52093. Supplementary material 2: immune infiltration analysis of GSE52093. Supplementary material 3: qRT-PCR results of additional m6A regulators (YTHDF1, YTHDF2, YTHDF3, RRP8, ALKBF1, and ALKBF3) in human aortic dissection tissue and healthy aortic tissue, “∗” represents P < 0.05; “∗∗∗” represents P < 0.001. Supplementary material 4: the mRNA expression of SOD2 in human AD samples and normal samples. [file 3918393.f1.zip › Supplementary File 3-ALKBF3.pdf]

# RRP8

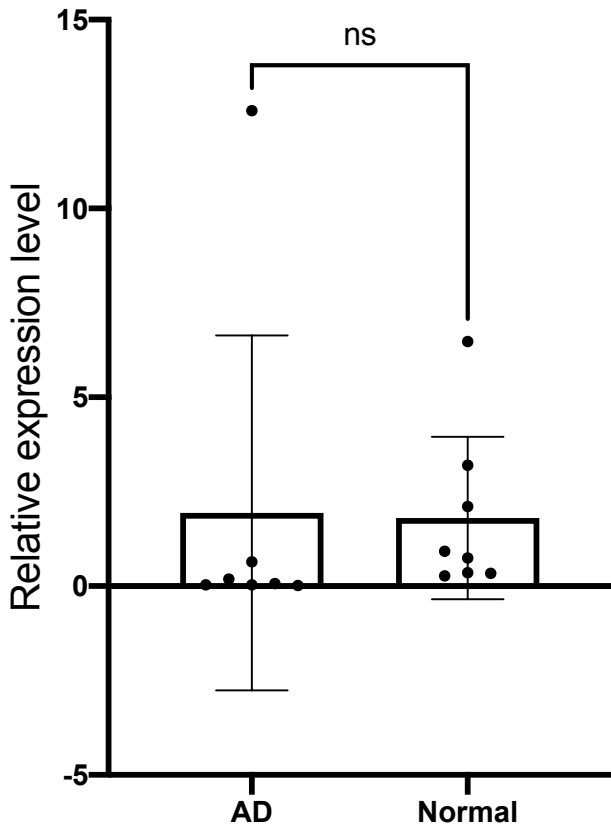

Supplement: Supplementary Materials — Supplementary material 1: differential expression analysis of genes in GSE52093. Supplementary material 2: immune infiltration analysis of GSE52093. Supplementary material 3: qRT-PCR results of additional m6A regulators (YTHDF1, YTHDF2, YTHDF3, RRP8, ALKBF1, and ALKBF3) in human aortic dissection tissue and healthy aortic tissue, “∗” represents P < 0.05; “∗∗∗” represents P < 0.001. Supplementary material 4: the mRNA expression of SOD2 in human AD samples and normal samples. [file 3918393.f1.zip › Supplementary File 3-RRP8.pdf]

# YTHDF1

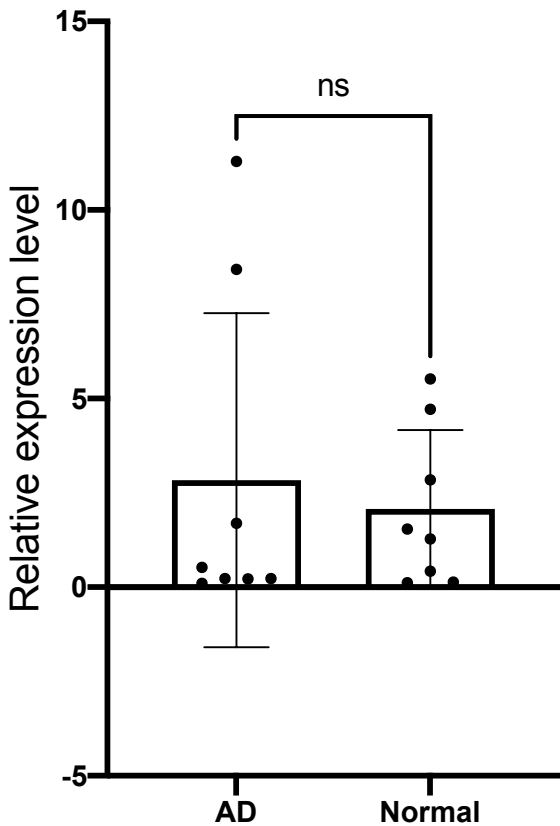

Supplement: Supplementary Materials — Supplementary material 1: differential expression analysis of genes in GSE52093. Supplementary material 2: immune infiltration analysis of GSE52093. Supplementary material 3: qRT-PCR results of additional m6A regulators (YTHDF1, YTHDF2, YTHDF3, RRP8, ALKBF1, and ALKBF3) in human aortic dissection tissue and healthy aortic tissue, “∗” represents P < 0.05; “∗∗∗” represents P < 0.001. Supplementary material 4: the mRNA expression of SOD2 in human AD samples and normal samples. [file 3918393.f1.zip › Supplementary File 3-YTHDF1.pdf]

## YTHDF2

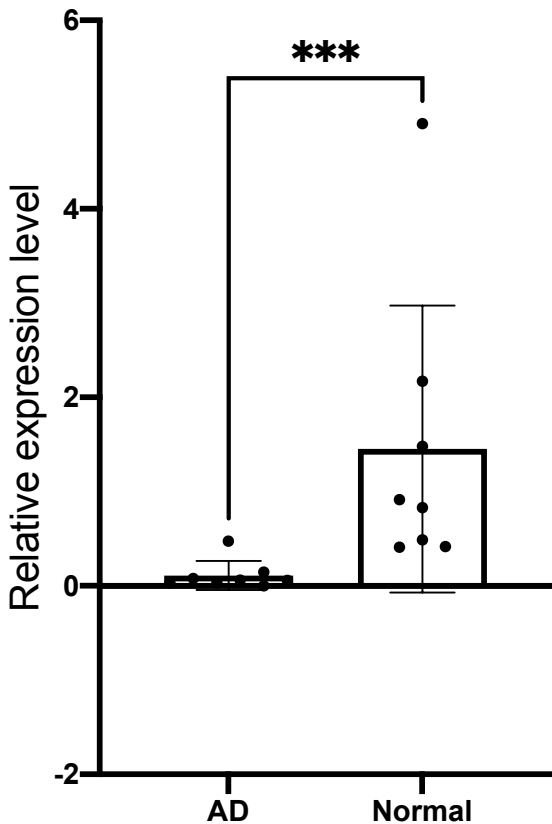

Supplement: Supplementary Materials — Supplementary material 1: differential expression analysis of genes in GSE52093. Supplementary material 2: immune infiltration analysis of GSE52093. Supplementary material 3: qRT-PCR results of additional m6A regulators (YTHDF1, YTHDF2, YTHDF3, RRP8, ALKBF1, and ALKBF3) in human aortic dissection tissue and healthy aortic tissue, “∗” represents P < 0.05; “∗∗∗” represents P < 0.001. Supplementary material 4: the mRNA expression of SOD2 in human AD samples and normal samples. [file 3918393.f1.zip › Supplementary File 3-YTHDF2.pdf]

# YTHDF3

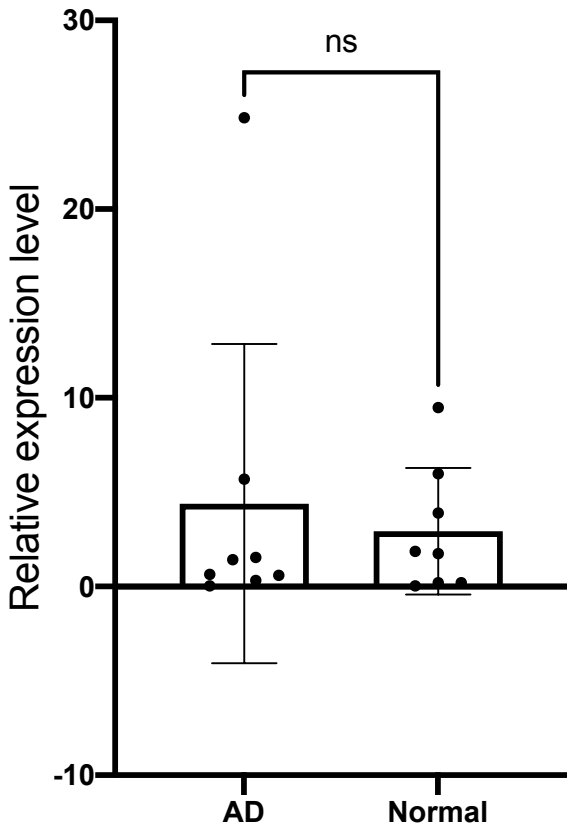

Supplement: Supplementary Materials — Supplementary material 1: differential expression analysis of genes in GSE52093. Supplementary material 2: immune infiltration analysis of GSE52093. Supplementary material 3: qRT-PCR results of additional m6A regulators (YTHDF1, YTHDF2, YTHDF3, RRP8, ALKBF1, and ALKBF3) in human aortic dissection tissue and healthy aortic tissue, “∗” represents P < 0.05; “∗∗∗” represents P < 0.001. Supplementary material 4: the mRNA expression of SOD2 in human AD samples and normal samples. [file 3918393.f1.zip › Supplementary File 3-YTHDF3.pdf]

# SOD2

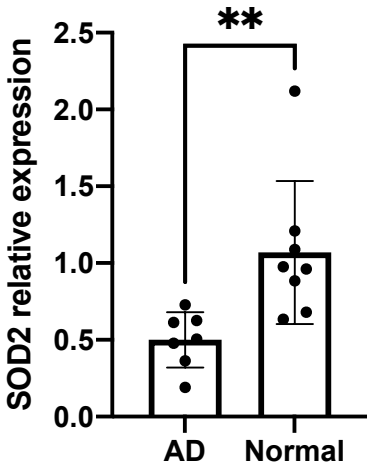

Supplement: Supplementary Materials — Supplementary material 1: differential expression analysis of genes in GSE52093. Supplementary material 2: immune infiltration analysis of GSE52093. Supplementary material 3: qRT-PCR results of additional m6A regulators (YTHDF1, YTHDF2, YTHDF3, RRP8, ALKBF1, and ALKBF3) in human aortic dissection tissue and healthy aortic tissue, “∗” represents P < 0.05; “∗∗∗” represents P < 0.001. Supplementary material 4: the mRNA expression of SOD2 in human AD samples and normal samples. [file 3918393.f1.zip › Supplementary File 4.pdf]
