# Supplementary material for: The Effect of N6-Methyladenosine Regulators and m6A Reader YTHDC1-Mediated N6-Methyladenosine Modification Is Involved in Oxidative Stress in Human Aortic Dissection
Source: Oxid Med Cell Longev. 2023 Feb 9;2023:3918393. doi: 10.1155/2023/3918393 (PMC9935809; doi:10.1155/2023/3918393)
Supplement: Supplementary Materials — Supplementary material 1: differential expression analysis of genes in GSE52093. Supplementary material 2: immune infiltration analysis of GSE52093. Supplementary material 3: qRT-PCR results of additional m6A regulators (YTHDF1, YTHDF2, YTHDF3, RRP8, ALKBF1, and ALKBF3) in human aortic dissection tissue and healthy aortic tissue, “∗” represents P < 0.05; “∗∗∗” represents P < 0.001. Supplementary material 4: the mRNA expression of SOD2 in human AD samples and normal samples. [file 3918393.f1.zip › Supplementary File 1.doc]

**GeneID	logFC	adj.P.Val**

REEP1	-4.179709414	0.000166494

SLC2A6	2.189863998	0.000166494

ACTC1	-5.722002646	0.000166494

RPL29	3.588154388	0.000250001

CCDC34	2.27274848	0.000250001

SLC22A4	2.586590984	0.000250001

GINS2	4.661730294	0.000250001

RYR2	-2.579124184	0.000250001

NME1	2.48177726	0.000276941

SLC20A1	1.759192934	0.00032286

SLMAP	-2.738358078	0.000328137

TINAGL1	-2.803954562	0.000328137

TMEM158	3.754496236	0.000328137

RPS7	2.005257884	0.000328137

CKB	-3.13573759	0.000328137

STS-1	3.0336992	0.00039056

JAK2	-1.980581672	0.000446748

CNN1	-3.987390364	0.000446748

M6PRBP1	1.55685891	0.000456417

FCER1A	-4.421640996	0.000467442

SRPRB	1.442876162	0.000467442

TMEM30B	-2.94558387	0.000467442

MAPKAPK3	1.702318108	0.000467442

PLAUR	3.003099622	0.000467442

C1QTNF7	-3.890230744	0.000483862

CLCF1	2.617946662	0.000483862

RAB13	1.739199168	0.000483862

GEM	-3.119241738	0.000488329

CCDC146	-3.868765092	0.000493167

SERPINE1	2.23744318	0.000493167

RPP40	1.540005182	0.000493167

FZD7	-1.913485496	0.000493167

ELOVL6	2.490618646	0.00052041

NT5DC3	-2.118161592	0.000549089

PKD2	-2.273050062	0.000549123

MAD2L2	1.551979008	0.00055048

C5orf24	-1.797219768	0.00055048

PHLDA1	2.612986166	0.00055048

ZNF323	-2.173309058	0.00055048

KIAA1671	-2.30689	0.00055048

UNC84A	-1.644210214	0.000561194

PDLIM5	-1.69244028	0.000561194

ABHD5	1.464321302	0.000561194

EFHD1	-3.01290757	0.000561194

LOC440731	1.887374426	0.000561439

FILIP1L	-2.837978592	0.000561439

ADARB1	-1.983671504	0.000604408

THSD4	-2.069018396	0.000620297

PDZRN3	-2.272834344	0.000620297

C4orf18	-1.442334196	0.000630067

NEXN	-3.000228662	0.000630067

FHOD3	-1.93884898	0.000630067

ECT2	2.510835144	0.000630067

ADRA1B	-3.274907616	0.00063199

ZNF364	-1.62164715	0.000646504

CCRN4L	2.280890958	0.000706456

UBE2T	2.951188786	0.000706456

LHFP	-1.580113794	0.000706456

CFL2	-1.165476958	0.000755596

C9orf30	1.467690598	0.000756832

C6orf145	-1.736201816	0.000760143

LOC441019	2.999721686	0.000774898

PGM5	-2.762254406	0.000779083

PPPDE2	1.578461006	0.000779083

ANGPTL4	4.216974184	0.000779083

LOC647886	-2.298549998	0.000802839

IPO4	2.024423948	0.000817934

MCM4	2.635107516	0.000822789

LHFPL2	1.392246012	0.000822789

PPP2R2B	-1.755074382	0.000822789

ITGA5	2.554980762	0.000842524

FKBP11	2.156594878	0.000842524

RAG1AP1	1.308526536	0.000842524

CASZ1	-1.75899097	0.000851493

CD82	1.825911178	0.000851493

NOP14	1.546757364	0.000851493

ACADL	-2.66813349	0.000851493

CD59	2.297715124	0.000851493

C6orf129	1.48353831	0.000851493

CAMK2G	-1.975177176	0.000851493

MGC87042	2.750004686	0.000851493

NANS	1.550300388	0.000851493

MN1	-2.331259934	0.000851493

TRIP13	3.374136766	0.000851493

MT2A	3.17952917	0.000851493

PI15	5.605006722	0.000851493

BCKDHB	-1.166005584	0.000851493

NT5DC2	2.751633418	0.000851493

DHCR24	1.725457476	0.000851493

HMGA1	2.492507182	0.000851493

MRGPRF	-2.178985724	0.000851493

CSRP1	-1.535196454	0.000865421

UBXN8	1.93383152	0.000901943

LGI1	-2.937632308	0.000901943

BOP1	1.35399608	0.000901943

PTGS1	2.35237905	0.000901943

PAICS	1.404590254	0.000901943

S100A3	3.17785347	0.000901943

GGCT	1.251980532	0.000901943

TMEM47	-2.592007908	0.000901943

PRUNE2	-2.30797537	0.000901943

METTL1	1.527529696	0.000913572

TIMP1	1.162354664	0.000934357

PLCB4	-2.019968488	0.000934357

SLC38A5	2.195325934	0.000934357

MYOM1	-2.871391396	0.000937627

JPH2	-1.62824679	0.000956485

ATL3	1.426089838	0.000977068

RANGAP1	1.673537556	0.000992665

RIMS3	-2.43423274	0.001014402

PPP1R12A	-1.247862338	0.001014402

CHEK1	3.137361228	0.001040627

APITD1	1.393290602	0.001054415

LOC286016	1.539174684	0.001054415

MGC4677	1.92154342	0.001054415

LDOC1	-2.295703786	0.001054415

ITGA2	2.560950598	0.001054415

MTP18	3.658482386	0.001054415

LOC731366	-1.454499002	0.001055919

WDR54	1.53031036	0.001070813

SHC1	1.238028154	0.001070813

ZDHHC17	-1.108957088	0.001070813

GRAMD3	-1.392822484	0.001070813

RNF115	-1.958246566	0.001070813

HIF1A	1.615135276	0.001070813

LIMS2	-2.133408742	0.001070813

GALK1	1.235703334	0.001070813

WTIP	-1.692631988	0.001070813

MCTS1	1.232378736	0.001070813

DUSP14	1.143188066	0.001072649

HIST2H2AA4	1.679539274	0.001072649

GSTZ1	1.459555634	0.001072649

WDR4	1.668045684	0.00108452

LOC728264	-1.945555482	0.001088162

YARS	1.236518736	0.00110364

WFS1	-1.503093288	0.00110364

PPP1R14A	-2.750955082	0.00110364

MRPL12	1.593819342	0.00110364

HIST1H1C	1.718055108	0.00110364

RP11-529I10.4	1.460295668	0.001160257

EFTUD2	1.461917272	0.001160257

SERPINH1	2.20029904	0.001160257

TSPAN5	1.517990938	0.001163911

STK38L	-2.124055334	0.001178651

HLF	-3.365009458	0.001215046

TUBB3	5.285561138	0.001236191

TFRC	1.867154052	0.001240323

CDC7	1.99472687	0.001240345

SLC25A22	2.309141152	0.001240345

KANK1	-2.32559959	0.001240345

TIMELESS	2.340436802	0.001240345

LOC202227	1.664745838	0.001278695

MRPL37	1.039223002	0.001281228

BAMBI	-2.364675774	0.001286293

MT1A	2.604173374	0.00132809

PLN	-3.241180542	0.001343267

TXNDC17	1.000698296	0.001347799

PITPNM1	1.485086368	0.001347799

STEAP1	2.908834742	0.001364138

TNIP2	1.263457536	0.001364138

LOC100131857	-2.95663384	0.001373193

RPUSD1	1.378454058	0.001373965

UGDH	1.423561822	0.001373965

RPL36A	2.061617774	0.001373965

COBLL1	-1.399356246	0.001373965

MXRA7	-1.248303798	0.001373965

EIF4A1	1.203524608	0.001402232

GMNN	1.698320658	0.001409827

FEN1	2.694813254	0.001409827

FNDC4	1.883628346	0.001411288

SH3BGRL3	1.58625923	0.001411288

PER3	-2.85348962	0.001434178

NPM3	1.75721898	0.001434178

MIPEP	1.09404003	0.001434178

IL17D	-1.986308038	0.001434178

DUSP8	-1.44113292	0.001450898

STX1A	1.983895758	0.001450898

BRP44L	-1.176487976	0.001450898

ALG3	1.331506228	0.001470313

TJAP1	-1.49577943	0.001470313

MEF2C	-1.530544254	0.001470313

LOC652545	1.329839564	0.001470313

WDR51A	2.618398052	0.001487114

IRAK1	1.454110768	0.001487114

UHRF1	3.204152486	0.001487114

TBC1D16	1.578666688	0.001487114

FZD1	-1.280105606	0.001487114

CDKN1C	-1.940017474	0.001487114

MBOAT7	1.265945588	0.001507318

ACTG2	-2.143886412	0.001519524

LOC646993	2.768016728	0.001525295

IL1R1	1.46869697	0.001525295

FLJ21986	-2.780195736	0.001525295

MIR21	2.513182638	0.00152989

APOO	1.298407428	0.001556135

PPIL5	1.287228022	0.001556135

BOLA3	1.201495758	0.001556135

SFRP1	-2.469969338	0.001556135

SLC25A4	-2.058199498	0.001556135

ADK	1.401890856	0.001556135

INPP5A	-1.45848133	0.001566426

BOLA2	1.08368193	0.001581684

S100A11	1.11058703	0.001586907

G6PD	1.35927108	0.001586907

GPN2	0.888241268	0.001592199

TTC26	1.426135772	0.001598878

RCAN2	-2.931289016	0.001598878

PPM1B	-1.117674952	0.001598878

CHST1	2.036964948	0.001598878

CARD9	1.943094664	0.001598878

SLC9A1	0.970601114	0.001613556

ALDH2	-1.372885112	0.001625406

PKM2	1.661690226	0.001625406

PDIA4	1.837005206	0.001625406

GPD1L	-1.59748905	0.001625406

LMO3	-2.425570106	0.001625406

CCDC81	-2.441266414	0.001625406

RRP12	1.765621162	0.001625406

CISD1	1.315996342	0.001690564

B9D2	1.679279532	0.001690564

UNC13C	-3.453356802	0.001690564

PPP1R12B	-2.862161582	0.001697215

MVP	1.084649826	0.001751547

SHMT2	1.54789694	0.001768602

PLOD2	1.364601466	0.001778945

NRIP3	1.137819626	0.001779864

VARS	1.221934632	0.00179236

MCM2	2.956711534	0.001804731

CXorf64	1.378351512	0.001804731

CDC20	4.306854534	0.0018244

FOSL1	2.420478754	0.0018244

SOX4	1.526670508	0.001833344

P2RY6	1.808421	0.001833344

MC1R	1.454869834	0.001850107

GSN	-1.693782092	0.001850107

LRRC1	-1.242487122	0.001850107

MYLK	-2.348300446	0.001850107

NEK6	2.077096182	0.001850107

HYAL3	1.375792242	0.001850107

C9orf114	1.048716468	0.001886671

BMP1	1.497821518	0.001886671

MYL9	-2.220450764	0.001886671

CDKN3	4.101514312	0.001903185

CRIM1	-2.660321928	0.001974257

ANKS1A	-1.27291023	0.001977058

XPO5	0.985127514	0.001987916

ACY1	1.419744776	0.001987916

UBTD1	1.262942728	0.001988607

NLGN1	-1.836104074	0.002005434

FNBP1	-1.000231714	0.002005434

ADHFE1	-2.468187246	0.002005434

LOC100130511	0.876014506	0.002005434

OSBPL1A	-1.063617608	0.002005434

LOC728698	1.788761372	0.002005434

SARS2	1.128770936	0.002005434

CYFIP2	-2.490674346	0.002005434

DARS2	1.612886038	0.002005434

C10orf125	1.431482452	0.002005434

FXYD5	1.30971851	0.002005434

GART	1.092704228	0.002005434

CD9	-1.550975324	0.002005434

SMOX	1.48845142	0.002005434

S100A16	1.595855844	0.002016142

CAP2	-2.4463161	0.002017835

HNRNPF	1.328353614	0.002017835

SLC24A4	-2.123658176	0.002017835

TCEB3	1.42538494	0.002017835

C21orf7	-2.225311788	0.002019401

MYRIP	-2.338308608	0.002037637

SDK1	-1.83074577	0.002054313

WIBG	0.913779488	0.002067419

REM1	-2.106457072	0.002074617

FNDC5	-2.968650346	0.00208256

C20orf29	1.247031428	0.00208256

NXPH3	-3.584982966	0.00208256

CRYL1	-1.427197152	0.00208256

SCARNA10	1.500884566	0.00208256

AASS	-1.356739934	0.002097296

SBNO2	2.02889025	0.002097296

SYNM	-1.991511122	0.002097296

PTTG3P	3.21874532	0.002097366

HGF	2.884196554	0.002097366

C19orf48	1.8810191	0.002112369

POLE2	2.11619865	0.002119512

EIF4E2	1.21732948	0.002119512

ATG7	1.057295926	0.002119512

UBFD1	1.510140376	0.002158596

C1orf24	-1.697614064	0.002160025

RCC2	1.278282706	0.002164106

LOC650518	0.888802812	0.002169115

ALDH1A1	-2.318866612	0.002169115

MRPS2	1.136918636	0.002188001

PRRX1	1.084659212	0.002194623

LOC100129267	1.24414623	0.002194623

CCNE1	2.957055698	0.002194623

MCM6	0.995895682	0.002194623

COL6A3	2.082536702	0.002194623

C16orf35	1.09497218	0.002200586

LOC151579	1.059486918	0.002200586

FLJ10781	-2.63595901	0.002215487

BYSL	1.672887998	0.002215487

MGC18216	-1.33432925	0.002215487

TBL1X	-1.162718686	0.002220562

VLDLR	-1.28878476	0.002243781

NRBP2	-1.417359502	0.002243781

ENO1	1.169284246	0.002249918

AMPH	-2.611673262	0.002249918

CASQ2	-4.292976162	0.002249918

MLEC	1.191003792	0.002249918

DHX37	1.258271868	0.002277005

ANXA2P3	1.22052183	0.002315125

YIPF1	0.771337368	0.002315125

SNORD83B	1.812364348	0.002315125

HADH	-0.99579437	0.002338734

PEMT	1.05468512	0.002338734

CYLD	-0.935496316	0.002345529

LOC643856	1.20687797	0.002351432

SLC16A3	2.455547884	0.002351432

MELK	4.012491558	0.002351432

ADCY9	-1.468641488	0.002351432

MT1E	1.715617876	0.002351432

PYGL	1.45505117	0.002351432

PFAAP5	-1.543165858	0.002351432

PDCD5	0.801519728	0.002351432

GK	1.854074214	0.002351432

PFAS	1.484976128	0.002351432

GPR137	0.902307884	0.002351935

C5orf23	-2.665967884	0.002351935

SLC7A5	3.456274062	0.002351935

MPDU1	2.015626464	0.002353722

MRPS12	1.031251104	0.002366445

ITGB3BP	1.415378608	0.002366445

DSTN	-1.713485144	0.002366445

POLA2	1.590850454	0.002366445

LOC100129673	1.367972092	0.002366445

LAMA3	-1.990519472	0.002390951

HIST2H2AA3	1.640923814	0.002392033

HK2	1.392724996	0.002404748

CANT1	0.930025038	0.002404748

ACTN4	-1.956694978	0.002404748

MYOZ2	-3.524699742	0.002418582

C11orf54	-0.867285166	0.002465883

TARBP2	1.343043346	0.00247362

NAT14	1.359834782	0.00247872

PANX1	0.966391384	0.002483155

CHSY1	1.04081895	0.002483155

HMBS	1.820867302	0.002496801

PSMB3	0.78686549	0.002500115

TRIAP1	1.023984122	0.002500115

LOC100129218	1.61063168	0.002500115

C19orf24	1.338059118	0.002500115

CALD1	-1.565358738	0.002500115

LOC100129118	1.172556262	0.002500115

FAM57A	1.104597006	0.002500115

PTPLA	-2.09713278	0.00250405

C17orf53	2.46577864	0.002504396

ANGPT2	2.082964372	0.002514541

TTLL7	-2.355213572	0.00254231

TKT	1.166291068	0.002579344

GGTA1	-2.756650512	0.002584319

IMPDH1	1.193878348	0.002617713

C16orf5	-1.62467489	0.002669655

GRWD1	1.058116666	0.002669655

ACOT7	1.544668528	0.002677692

CHES1	-1.250532966	0.002683457

E2F3	0.989337742	0.002683457

RAD54L	2.840375644	0.002683471

ZDHHC9	1.267699604	0.002685664

RERG	-2.452885368	0.002685664

CTF1	-1.579074882	0.002685664

TOMM40	1.179086858	0.002685664

HELLS	1.5286434	0.002685664

SNORD96A	3.006997136	0.002685664

PTRH1	1.46205655	0.002685664

ZNF25	-1.0933061	0.002685664

ANKRD6	-1.77276578	0.002699458

LBH	-1.466286674	0.002699458

C1orf198	-1.734208592	0.002706994

Magmas	0.984534394	0.002721553

IKBIP	1.73988855	0.002755064

EDEM2	1.327843798	0.002755064

SORBS1	-2.035513712	0.002755064

LOC730052	0.940006358	0.002755064

ANXA2P1	1.138085646	0.002755064

KPTN	1.246631348	0.002770895

SORT1	-2.025527852	0.002770895

LRP8	2.125960292	0.002812789

DACT1	-1.851951296	0.00281728

PLSCR4	-1.551583062	0.00281728

TUBA3D	1.198125208	0.00281728

ARHGAP12	-1.087770776	0.002830282

NEDD9	-2.014263186	0.00283191

PER2	-2.361800864	0.00283191

KIF14	2.323110372	0.002833187

ATP1B3	1.13449425	0.002836755

MTE	2.557633488	0.002859177

CLK1	-0.932468044	0.00287081

HSPB8	-2.222739942	0.002876052

SPHK1	2.703635374	0.002876345

LOC653394	-2.359546354	0.002876345

C3orf70	-2.235736294	0.002876345

CCDC86	1.308061554	0.002876345

AIMP2	0.97236583	0.002876345

FLJ45032	1.044396048	0.002876345

MPP2	-1.470077372	0.002918217

DCUN1D5	0.931694984	0.002918217

SESN3	1.996445146	0.002918217

SLC39A14	2.299397642	0.002918217

ZNF438	-0.952264826	0.002918217

TSPAN17	1.121299872	0.002918217

GEMIN6	0.889344902	0.002922208

KLHDC5	-1.41170532	0.002930433

ANO1	-2.184982126	0.002930433

MGST3	-1.012165214	0.002930433

NCLN	1.47520793	0.002930433

PDSS1	1.463666494	0.002932308

APBB2	-1.221265994	0.002936348

BZW2	1.459077326	0.002936348

LOC100130919	1.361715748	0.002962313

ST6GALNAC4	1.51721272	0.002962313

RAB23	-1.433004972	0.002962313

HCFC1R1	-1.509384772	0.002962313

LOC652264	-1.151521578	0.002970643

CAV2	-1.154930368	0.002976462

NSDHL	0.887251248	0.003001367

MRPL2	1.288835938	0.003001367

PNPLA7	-1.320595096	0.003001367

PSMB5	0.811963922	0.003028067

C20orf194	-1.052131102	0.003028067

ANO6	-1.015531038	0.003028067

CYP27A1	-1.43971685	0.003028067

FMO3	-1.760136872	0.003044892

PSPH	1.551793868	0.003047547

LOC644101	1.234145364	0.003053353

C1GALT1	1.290028066	0.003053353

SLC35E4	1.307342972	0.003053353

DUS3L	1.22766016	0.003053353

EMP3	1.341343566	0.003053353

STYXL1	1.276636664	0.003053353

TPM1	-2.037185912	0.003053353

PURA	-1.058700326	0.003053353

PLD5	-3.582115102	0.003053353

ZDHHC16	1.063265618	0.003053353

SYTL2	-1.564016156	0.003053353

PNMAL1	-2.468088168	0.003058418

CYSLTR1	-1.925141106	0.003064133

EIF3C	0.94419681	0.003126377

GPR172A	1.173549632	0.003148955

SLC35B1	1.024549476	0.003155139

ANKRD29	-2.53598037	0.003175566

TOMM34	1.143821446	0.003175566

GATAD2A	1.011495748	0.003175566

OTUD1	-1.04607483	0.003175566

CKAP4	1.633461868	0.003179126

LOC283932	1.666565286	0.003203751

LOC391811	0.962931626	0.003217373

C14orf132	-1.879076922	0.003255473

EPM2AIP1	-1.033147092	0.003256011

SNORD13	-1.70525377	0.003276174

TYMS	2.920159376	0.003297573

CD46	-1.06637325	0.003297573

ATAD3A	1.51167504	0.003303526

CDCA5	3.699929056	0.003308445

LOC100133012	1.570858144	0.003343622

GLRX	2.292619904	0.003354436

C3orf58	-1.09312106	0.003355209

MLKL	1.239235354	0.003362447

NOV	-3.217616658	0.00336618

CPEB2	-1.314828914	0.00336618

DUS2L	1.260310104	0.00336618

MACF1	-1.00058695	0.00336618

KBTBD7	-1.130696238	0.00336618

HIST1H2BD	1.908082964	0.00336618

SGCD	-1.877464494	0.00336618

GLS	-1.459141934	0.00336618

LATS2	-1.419356012	0.00336618

RNF122	1.645905854	0.00336618

SF3B3	1.183005998	0.00336618

RFC4	1.285224088	0.00336618

BMPR1A	-1.583784888	0.00336618

PTTG1	2.648069112	0.00336618

POLR2H	0.800969908	0.00336618

HSPC171	0.943477566	0.00336618

CDK5	1.312412092	0.00336618

GSTO1	0.892220932	0.00336618

OSR2	-1.022750912	0.00338184

ITM2C	-1.974534084	0.003382504

N4BP2L1	-1.444202618	0.003397209

PHLDA2	2.938093076	0.003415033

CCDC121	-1.751784626	0.003415033

NP	2.604829986	0.003415033

KIAA0367	-2.07162447	0.003415033

CCNF	2.745100774	0.003415033

CIRH1A	0.898177802	0.003426141

TARDBP	-1.196163846	0.003441704

ZNF362	-1.12308905	0.003451487

TRAPPC2L	0.764919028	0.003474057

ANKRD12	-0.777012534	0.003474057

ZXDA	-1.356235374	0.003474057

CDCA4	1.440442182	0.003476483

LRRC59	1.584823954	0.003516873

NUDT5	1.27778495	0.003526687

SNORD36A	0.915298978	0.003526687

EXD3	-1.64220027	0.003587575

RERGL	-4.31518965	0.003598654

FHOD1	1.476747816	0.003608796

BRMS1	1.036616742	0.003608796

SYNC1	-2.25629725	0.003608796

RPL34	1.321247152	0.003613603

RPL23AP13	-1.181398428	0.003613603

TMEM16A	-2.18798241	0.003613603

RAGE	1.110103696	0.003613603

MRPL3	0.846206196	0.003613603

LOC727803	1.039881456	0.003613603

METTL11A	1.201900302	0.003613603

ATOX1	0.943942784	0.003613603

DBP	-1.52075947	0.003613603

TPI1	1.174407338	0.003613603

NUDT22	0.814759802	0.003613603

LARGE	-0.838261432	0.003613603

CBX7	-1.957358718	0.003613603

SDF2L1	1.685363872	0.003613603

LIMCH1	-1.98967536	0.003613603

PDE8B	-3.545105328	0.003614699

UCHL3	0.859084566	0.003614699

RCN3	1.793589564	0.003623776

SLC43A2	1.69161374	0.003623776

RARA	0.98362406	0.003658733

GARS	1.25901118	0.003663716

SMARCC1	0.707315438	0.003672291

NPY5R	-1.261550214	0.003705125

TIMM23	0.77756554	0.003711393

GAR1	1.086624166	0.00371852

CRY2	-1.312716668	0.003722197

TIPIN	1.710378518	0.003722197

CNKSR3	-1.287151434	0.003739321

ITPA	1.018675516	0.003749194

AGTR1	-2.719190822	0.003753444

LONRF1	-1.049478846	0.003753444

C6orf153	0.770978336	0.003763203

CSE1L	1.023205712	0.003763203

SENP7	-0.957977972	0.003763203

VARS2	1.002259148	0.003773329

IMP4	1.21986667	0.003811196

GMPPB	1.309679086	0.003811196

ACPL2	-1.68042121	0.003811196

NPTN	-0.993543022	0.003811196

SMARCB1	1.01393996	0.003811196

NFKBIZ	1.310323704	0.003819901

SUV39H1	1.02720258	0.003820004

MAD2L1	2.366495176	0.003836605

CYB5R2	1.186339364	0.003855136

HAS2	2.469331636	0.003860567

MEF2D	-1.11482078	0.003860567

PLEK2	2.440462642	0.00386243

TUB	-1.044174202	0.00386243

LOC653381	1.072000062	0.00386243

CKS2	2.465989168	0.003862463

CSNK1D	1.29463473	0.003862463

LOC653147	0.957414648	0.0038755

SPP1	3.613218802	0.0038755

TUBG1	1.240298462	0.0038755

FAM62B	-0.972093654	0.0038755

HSPC111	1.8090649	0.0038755

C15orf23	1.645852786	0.0038755

ANXA11	-1.08351579	0.0038755

PPAT	1.009568664	0.0038755

CDS2	-1.115974564	0.0038755

ZNF593	1.155360138	0.003882637

BACE2	0.903370092	0.003882637

COL9A2	-2.355712158	0.003882637

AMMECR1	1.084885654	0.003882637

CREBBP	-0.824865668	0.003882637

LAMA5	-1.066648526	0.003882637

LOC100130308	0.827974338	0.003888201

FAM20C	1.684445576	0.00389673

CDK2	1.093811562	0.003902836

MBNL1	-1.079747472	0.003906231

C1orf112	1.392646852	0.003906231

DACT3	-1.70207168	0.003906231

MFAP4	-1.994327356	0.00390959

C6orf125	1.201764638	0.00391045

TMED4	-0.987579916	0.003916619

PSME4	1.126210638	0.003917142

RRAS	-1.125334368	0.003931495

PCNA	1.41910417	0.003964752

KLHDC1	-2.151665172	0.003970718

PMM2	1.025496404	0.003976605

IFI44L	-1.935663534	0.003979947

ILK	-1.11110075	0.003994059

CLIC1	0.91757624	0.004022596

SMAD9	-1.863566232	0.004024587

LOC100132510	2.437426232	0.004026246

HSPBL2	-1.230678648	0.004041107

MORC3	-1.27271029	0.004041107

C1orf63	-0.992047316	0.004053716

SSRP1	0.979956308	0.004056326

WDR18	1.005706422	0.004056326

DPP3	1.744101636	0.004056326

LOC650580	-1.703597124	0.004074607

AURKAIP1	0.928955874	0.004074607

LOC653972	0.82783106	0.004074607

TMEM49	1.335757096	0.004074607

CYB561D2	1.333304488	0.004147117

IMPDH2	0.96385318	0.004147117

JOSD2	1.420583744	0.004147117

TMEM141	1.138723212	0.004149876

GABARAPL2	-0.870710372	0.004151088

ZXDB	-1.258425686	0.004159925

ANKS1B	-2.14478308	0.004164765

CAND2	-1.866665952	0.004171239

FUCA2	1.2548826	0.004188157

LOC728787	0.692747534	0.004191812

CPSF3	0.877555416	0.004207921

HARS	0.798721376	0.004232435

DPAGT1	0.902934974	0.004241168

ADAMTSL5	-1.536475698	0.004241168

LOC646044	0.90566113	0.004241168

MRPL15	1.103161316	0.004241168

ITGA7	-1.636567316	0.004252711

C3orf46	-1.382113372	0.004272852

ARHGAP22	2.526087904	0.004293454

ACVR2A	-1.013404684	0.004321172

ANPEP	3.30001161	0.004340699

RTN1	-2.71669668	0.004361408

H2AFZ	0.884876272	0.004389781

BACH2	-1.95791053	0.004389781

WDHD1	1.922530248	0.004389781

YAP1	-1.367419424	0.004389781

RAB9B	-2.629211396	0.004402979

VAMP1	-1.947044302	0.004402979

TRAF5	-1.63692273	0.004405861

UCA1	-2.380882794	0.004433336

LOC646038	0.910805734	0.004440633

NUP37	0.85338919	0.004440633

SH3BGRL	-1.35146076	0.004440633

PROM1	-4.407429522	0.004440633

LOC729774	1.091533248	0.004453234

RFC5	0.753944894	0.004468526

LOC345041	1.146569922	0.004473249

PCGF2	-0.82837685	0.004497441

KAT2B	-1.190238146	0.004499242

LOC643733	-1.511579878	0.004499341

IL6	5.583193084	0.004510495

UAP1	1.197634908	0.004514624

TRPC1	-1.590471952	0.004514624

C12orf11	0.992216058	0.004514624

ARID4A	-0.931346804	0.004514624

GTF3A	1.03469234	0.004524463

RBM27	-1.20767943	0.004535441

VGLL3	-1.785445644	0.004568929

LOC648526	-1.87955676	0.004581822

SLC25A23	-1.087648786	0.004581822

ECE1	1.533814654	0.004582916

C3orf31	0.771902674	0.004586474

PLCH2	-0.7034157	0.004586474

CENPN	2.748906804	0.004615579

WDR81	1.346115376	0.004615606

NOC2L	1.012980956	0.004628859

COX5A	0.700956622	0.004634706

TP53BP2	-1.358431338	0.004634706

KCTD10	-1.532096466	0.004634706

RASL11B	-2.177088944	0.004634706

UPP1	1.638317918	0.004634706

STOML2	0.833665088	0.004634706

RAI2	-1.833289228	0.004636061

FAM129B	1.178110878	0.004636267

LAGE3	1.05692082	0.004657897

ODF2	0.893831082	0.004657897

ATP5J2	0.881725224	0.004657897

CENPF	3.130018446	0.004664531

FMO4	-1.676471674	0.004664531

PPP2CB	-1.084225042	0.004672192

LIMK1	1.87613643	0.004672192

ZBTB20	-1.140900152	0.004672192

PRPF4	0.88150675	0.004672192

ATF6	0.908351336	0.004672192

GOT2	1.003725662	0.004672192

CSRP2	-1.266435384	0.004672192

FGL2	-1.828531974	0.004701989

ROBLD3	0.908000512	0.004701989

AGTRAP	1.723265456	0.004701989

C19orf28	1.264500414	0.004701989

RALGPS1	-1.360062076	0.004701989

DDX52	0.847756716	0.004725652

EBF1	-0.973240212	0.004748289

ATPGD1	-2.489193186	0.004774377

C14orf142	0.798354976	0.004774377

TMEM167A	0.979931926	0.004779405

RBMS3	-1.622008096	0.004865287

MAPKAP1	0.90705553	0.004873259

FAS	0.929763488	0.004873259

PDZD4	-1.77030801	0.004886852

SNORD80	1.214527506	0.004919357

C11orf48	1.104591132	0.004919357

AP2S1	1.128463776	0.004919357

ANXA2	0.879156176	0.004919357

TEAD4	1.927898878	0.004919357

PCGF5	-1.669475298	0.004919357

LOC401152	-1.286705308	0.004919357

YARS2	0.858150422	0.004919357

RNF146	-1.065373844	0.004919357

LOC729406	1.226935318	0.004919357

FARSA	1.116597322	0.004919357

HN1	1.334587726	0.004919357

EPS15	-1.042146282	0.004919357

RTN4IP1	0.873744692	0.004919357

SMTN	-1.175764284	0.004919357

RGS2	2.164947564	0.004990963

TNC	2.622189718	0.004992919

LOC100129034	0.813386566	0.005008778

GPER	-1.447237114	0.005010169

ALG8	0.854398302	0.005034552

LOC392285	0.790411116	0.005034552

HIST2H2AC	1.483952176	0.005034552

NR3C2	-2.159158698	0.005034552

SNORD114-1	1.254094588	0.005034552

ZNF177	-0.924076262	0.005037494

PUS1	1.125989278	0.005037588

COL28A1	-2.652939152	0.005063945

PUS7	1.094269984	0.005063945

SNORD104	1.008055208	0.005063945

C5orf41	-1.455256854	0.005076113

DBNL	0.79045398	0.005076113

RPL26L1	0.75042951	0.005076113

HSD17B8	-1.010056676	0.005076113

YWHAE	0.829450728	0.005076202

ARHGEF9	-1.50731894	0.005076202

ADSL	0.74180984	0.005076202

UCK2	1.31986447	0.005176122

IRF2BP2	-0.907036464	0.005176122

LRP5	-1.289315094	0.005176122

SNAI2	1.638380478	0.005176122

KIF20A	5.107262274	0.005205791

ANKRD37	-1.655488652	0.005205791

NRAS	1.06738563	0.005205791

CCL2	2.203209488	0.005228445

WASF3	-1.408794988	0.005246286

TIMP3	-2.99395934	0.005246286

ECHDC2	-1.29383356	0.00524682

NME2	0.894840256	0.005253088

HDHD1A	0.874364614	0.005255127

STRA13	1.032772532	0.005255127

FURIN	1.457897988	0.005255127

LOC648470	-1.322730458	0.005271118

SOX15	-2.488479916	0.005289314

C3orf75	0.625094914	0.005301252

GSPT1	0.99130955	0.005329326

OIP5	2.227803378	0.005336666

TTC39C	1.985407754	0.00536821

DUSP3	-0.837560524	0.005373327

WDSOF1	1.140484436	0.005380351

C16orf93	1.806299232	0.005380351

NOP16	1.682737064	0.005401031

ITPKC	1.216490626	0.005401031

TOB1	-1.070499872	0.005401031

LOC645993	-2.410268646	0.005413781

PRMT5	0.869610004	0.005414241

SYNCRIP	0.989700768	0.005414241

LIN7B	-1.413132076	0.005441491

PSMB2	0.876743868	0.005465261

VIPR2	-2.668514526	0.005465261

HNRNPAB	0.900405446	0.005465261

AMT	-1.497987234	0.005465261

AOF2	0.77837587	0.005476827

LOC647856	0.814407596	0.005476827

FUT8	1.189651224	0.005476827

IGFBP3	2.958376026	0.005476827

CSTF2	0.88450368	0.005476827

ATOH8	-1.854880128	0.005476827

NPNT	-2.325038054	0.005476827

ICAM3	1.067830382	0.00548486

C20orf127	1.927829474	0.00550022

RNASEH2A	1.970503218	0.005530367

ZFP106	-0.978622248	0.005552531

SOX8	-1.31386856	0.005552531

CDH6	1.50128003	0.005552531

SLC5A6	1.4755645	0.005552531

ENOX2	-1.08501877	0.005552531

PPP2R4	0.709556842	0.005552531

SIRT1	-0.903186556	0.005552531

C8orf38	0.844837492	0.005552531

ADH5	-1.30277058	0.005552531

WDR12	1.106298592	0.005552531

ABCC3	2.040637996	0.005552531

VEGFB	-0.818532668	0.005552531

ZMYND19	1.175882994	0.005552531

CAB39	-0.688166746	0.005594083

LOC391075	0.972613164	0.005622982

CD302	-1.820295676	0.005643822

RUSC2	-1.385807452	0.005659488

TRIB3	1.85167136	0.005663985

FAM120A	0.690037732	0.005685619

NOP2	1.157698042	0.005742424

XPNPEP1	0.634793218	0.00574664

ESR1	-1.785670584	0.00574664

PSMG1	1.05753271	0.00574664

C22orf32	-1.5933476	0.00574664

KITLG	1.30704166	0.00574664

CBX3	0.884258066	0.00574664

GPI	0.93378841	0.00574664

MPP7	-2.569222706	0.00574664

RNF216	-0.849211882	0.00574664

ZBED4	0.715021708	0.00574664

PLOD3	0.927282198	0.005769151

LOC653888	1.488416992	0.005781355

MTRF1	0.914302198	0.005781355

SPARCL1	-2.078527298	0.005781355

FAM13C1	-1.5431123	0.005813266

SLC35B2	1.374250988	0.005814987

ST3GAL2	1.407432348	0.005814987

C9orf69	0.922113606	0.005836219

SRM	1.446418156	0.005896926

AURKA	3.268847198	0.005908223

APH1B	0.971923874	0.005909875

PES1	1.04284011	0.005913758

EIF3B	0.810328098	0.005919241

HRCT1	-3.116309916	0.005919241

SAMM50	0.742944538	0.005919241

YPEL1	-2.785428538	0.005930609

DTL	2.879719514	0.005950794

RGS5	-2.404620514	0.005961536

C1orf212	0.998312914	0.00597223

DYNC1I1	-1.832569064	0.005972668

FTHL2	0.965549938	0.005972668

LMNB2	1.666808698	0.005972668

CD63	0.758101926	0.005972668

PRIM1	1.06802034	0.005972668

HDDC3	0.86653087	0.005972668

CCDC77	0.798779488	0.005972668

FYCO1	-1.308227422	0.005978786

FILIP1	-1.353745654	0.005991876

LOC100128266	1.148144932	0.005991876

BRCA1	1.298357346	0.005999215

LOC100130506	-2.043316868	0.006009228

FNIP1	-0.78988873	0.006020954

MSRB3	-1.63113808	0.006037167

MYO1F	2.106294064	0.006038776

UBXN11	1.26662245	0.006038776

CCDC123	1.165294628	0.006044899

LOC387882	-2.20040723	0.006064754

BMPR2	-0.931221334	0.006084238

CSAD	-1.348753766	0.006084238

SFRS18	-0.965302978	0.006089048

LOC730132	1.578571516	0.006095383

GRK5	-1.053545902	0.006095383

GTF2F1	0.801602224	0.006095383

DNAJB11	1.084687298	0.006095383

DMPK	-2.603849534	0.006095383

RFX2	-1.896094312	0.006095719

RAN	1.022931938	0.006098014

LOC100129297	0.738522138	0.006098014

ZNF469	1.577747604	0.006098014

HAUS8	0.979885216	0.006098014

ARSB	0.985096708	0.006098014

F12	1.618693752	0.006117141

HIPK2	1.250184662	0.006117141

PKNOX2	1.706685102	0.006117141

ARPC5L	1.387284886	0.006126629

RBL2	-0.791408974	0.006165351

PSMD11	0.872493188	0.006177856

CCDC99	1.361446386	0.006204869

SLC39A3	0.71342865	0.00621423

LOC728908	1.033676018	0.00621423

CIAPIN1	0.824096114	0.006220575

TMEM159	-1.56432467	0.006220575

DCTPP1	0.958406018	0.006221555

LOC645762	0.995841564	0.006269765

JUNB	2.169225264	0.006269765

RUVBL1	1.385702382	0.006269765

VCP	0.818918454	0.006269765

C6orf108	1.02972857	0.006269765

CIB1	0.80469015	0.006332009

SLC1A5	1.42461892	0.006361181

FAM125B	-1.15687842	0.006378218

PRNP	-0.9505636	0.00637928

ANKRD49	-0.892460482	0.00637928

PRELID1	1.027279724	0.006408404

RANBP1	1.006513966	0.006408404

AP1S1	1.278970092	0.006408404

LOC100132060	-1.535436024	0.006408404

GLRX3	0.812014974	0.006408404

ASF1A	-0.744970672	0.006408404

C9orf116	1.422941624	0.006414888

CCDC107	-1.114291768	0.006440117

RACGAP1	1.980492022	0.00644603

C3orf64	-1.191052246	0.006447014

PREPL	-1.050630228	0.00646029

MYOCD	-3.36478216	0.00646029

CRY1	-1.005332808	0.006478992

ZWILCH	1.31897135	0.006478992

TMEM144	-1.224389422	0.006478992

LOC100131785	0.850938294	0.006478992

CCDC115	-0.993844068	0.006486374

MRPL14	0.822953486	0.006496382

PSMD5	0.959485292	0.006497313

LTBP4	-1.911038	0.006497313

SNX8	1.24426703	0.006497313

NCBP1	0.869014152	0.006497313

STXBP5	0.791241804	0.006500288

ZNF683	-1.684048648	0.006515951

SLC35C1	0.79068992	0.006515951

ATP13A2	1.086099862	0.006515951

HSF4	-1.19392189	0.006541309

RFC2	1.024790664	0.006541309

ODC1	1.254086868	0.006547463

HSPD1	0.92496189	0.00656626

FJX1	1.822056738	0.00656626

LOC647855	1.66363909	0.00656626

RUNX1	1.624279658	0.00656626

AGAP3	1.076990374	0.006575585

RRP7A	1.254772862	0.006579606

HIBCH	-0.83214915	0.006581763

LOC650157	0.825461814	0.006602436

TNFSF12	-1.058719574	0.006602436

EPHB1	2.015146136	0.006604109

SLC36A1	1.288672106	0.006608618

CDCA7	2.011213792	0.006621698

NRD1	0.724159384	0.006623406

PODN	-3.577124318	0.006623406

LOC731954	-1.537051558	0.006625521

MSN	0.831209826	0.006625521

LOC401317	-2.373507222	0.006625521

GP1BA	0.808425178	0.006625521

NFIA	-1.376759194	0.006635304

GNL2	0.68829077	0.006642886

SLIT2	-1.405933088	0.006657632

CHAF1B	1.922212074	0.006657632

LOC732415	2.265278384	0.006658598

PDGFRA	0.905330144	0.006705112

FLJ20699	0.827826492	0.006708701

PDE3B	-1.222266682	0.00673055

SLC6A1	-1.813813514	0.006730974

GNPNAT1	0.696516272	0.006739159

TXNL2	0.824623464	0.006743497

REV1	-0.794391508	0.006743497

ENC1	1.692814532	0.006761873

UBL3	-1.134030358	0.006790005

ABCB10	-0.803024292	0.006790005

RAB3GAP1	-0.871115604	0.006790005

SLC15A4	-0.814377046	0.006790005

PPRC1	0.824590226	0.006794424

NOL6	0.823415064	0.006804309

SLC35A2	0.870031552	0.00681912

UHRF2	0.832920662	0.006826243

LONRF2	-1.888746228	0.00683413

MAP4K3	-0.926407714	0.006865993

NTF3	-2.522681962	0.006944336

SAC3D1	0.965252724	0.006949576

PIK3R2	0.996129932	0.006995736

GRIA2	-3.934538966	0.006995736

GOSR2	0.762298128	0.006995736

LOC727761	1.16916766	0.006995736

C7orf58	-2.006366414	0.007020654

C21orf45	1.215994656	0.007020654

SLC16A10	2.351116636	0.007020654

PCSK7	1.036145792	0.007030773

LOC647054	-1.27765088	0.007030773

PAPD5	-0.966450324	0.007030773

PSMD14	0.848334424	0.007058645

MIR199A2	1.03326019	0.007070937

NBLA00301	-1.715808222	0.007088883

TLN1	-0.96313097	0.007106863

LPCAT3	1.052729542	0.007129321

LOC100130003	0.70351862	0.007135987

LMOD1	-2.506300254	0.007164421

EXOSC4	1.187346504	0.007164421

BTC	-3.065127962	0.007199951

PDCD6IP	-1.135958016	0.007221342

RBBP6	-0.909791224	0.007245362

RAB4A	-1.478106292	0.007245362

NACC2	1.789541812	0.00725655

ATP7A	-1.093022338	0.007273502

PRSS3	1.944205228	0.007273502

NDFIP1	-0.985871888	0.007285873

GDI2	0.67828478	0.007307194

PLOD1	1.083465044	0.007327664

LOC100128888	-1.421524802	0.007328665

CLEC5A	3.575290112	0.007341618

LOC646900	1.055728068	0.00734317

MRTO4	1.355395636	0.007384811

PAQR4	1.69366266	0.007417653

GPATCH4	1.467796352	0.007417653

ITPRIP	0.967869566	0.007428621

C17orf96	1.881128224	0.007428695

NOLC1	1.23115498	0.007444709

LOC644563	0.858740232	0.007454483

CREBZF	-1.025784598	0.007479665

C12orf10	0.65903753	0.007479665

ZBTB47	-1.131939322	0.007509281

MYC	2.427111368	0.007509281

CDKN1A	1.37597568	0.00753044

SSPN	-2.228388792	0.007568209

FIBP	0.94132456	0.007575876

PUM2	-0.697933834	0.007608707

ATP5G1	0.711330836	0.007614432

C5orf4	-1.28503105	0.007614432

SCO2	1.429652756	0.007620573

LOC100134361	-1.75239763	0.007632313

TP53I11	1.536933482	0.007666895

TTK	3.179950978	0.007666895

LOC728830	-1.136944544	0.00771022

TCTEX1D2	1.038226886	0.00771022

POLR3B	0.873424208	0.00771022

POMP	0.739297126	0.00771022

RPN1	0.845634262	0.00771022

RSU1	-0.826929784	0.00771022

CYP2U1	-1.58553602	0.00771022

SRRD	0.962092194	0.00771022

MYH10	-1.846220258	0.007721051

MRPL52	1.078975704	0.007737187

MRPL24	0.757608018	0.007754269

SDHAF2	0.743957652	0.007754269

DNAJC3	1.587909928	0.007754269

LOC729768	0.97097534	0.007754269

PDIA6	1.316530984	0.007754269

ENTPD3	-0.827787806	0.007782917

THOP1	1.47895363	0.007786296

RPS21	1.170363234	0.007794139

STXBP3	-0.874571422	0.007794139

STK19	0.798499244	0.00780067

FAM126B	-0.774764378	0.007813118

HSPC047	-1.361468226	0.007813118

SPCS3	1.260861448	0.007813118

C14orf143	1.236121738	0.007813118

SLC25A15	1.171528886	0.007829989

GPR126	-1.660042208	0.007829989

NNMT	1.45060477	0.007854489

TLE1	-1.0096237	0.007883717

GLCCI1	-2.042898148	0.007883717

LOC147727	-0.930953538	0.007890778

FADS1	0.90497743	0.0079028

LOC100132457	0.671819778	0.0079028

LOC286367	-1.427250442	0.00797324

KCNMB1	-1.998039468	0.00797324

CLASP2	-1.111904654	0.007997762

HSPB7	-2.329758928	0.007997762

TMEM19	-1.045712316	0.007997762

ENAH	-1.75419749	0.007999421

PGM3	1.090836316	0.007999421

B9D1	1.194199072	0.008024142

MCAT	0.915034748	0.008026407

POLQ	2.469290318	0.008035409

TATDN2	0.607101806	0.008035409

LRRN1	-1.883875792	0.00803551

OXCT1	-1.059185468	0.008049168

FANCG	1.277051858	0.008049168

ADCK4	1.02248668	0.008050555

TMEM178	-3.472682736	0.008067925

C20orf27	0.988608668	0.008074115

PPIH	0.85790641	0.008074115

SHQ1	1.048694904	0.008096552

RNF180	-1.392074926	0.008122529

BLMH	1.008866656	0.008122529

ICK	-0.95511078	0.008143308

SERAC1	-1.457243122	0.008148513

SPRED1	0.731991048	0.008148513

ALS2CR8	-1.281727378	0.008148513

LOC678655	-1.157263946	0.008148513

ADAM19	1.440043194	0.008157577

ARHGDIA	0.765664844	0.008192919

MYLIP	-2.05922264	0.008192919

PRKDC	0.93224757	0.008192919

ITGA8	-2.549950958	0.008197784

GNL3	0.934957036	0.00821376

DHRS4L2	1.099441704	0.008221231

RSBN1	-0.93366802	0.008221231

RUVBL2	0.808541086	0.008221231

KIAA0020	0.897517356	0.008226211

LYPLA2	1.047356126	0.008240366

SDSL	1.407733492	0.008249477

SERPINI1	-1.88319392	0.008249477

ERAL1	0.665500776	0.008249477

FHL1	-2.080149556	0.008277061

MRPL17	0.994884714	0.008293302

C17orf62	0.89270784	0.008293302

IQSEC1	-1.061265866	0.008293302

PET112L	0.884076748	0.008293302

PNN	-0.711234632	0.008293302

SORBS2	-2.02545054	0.008293302

PSMC3	0.60562523	0.008293302

C6orf173	2.147053328	0.008293302

DOCK1	-0.74452357	0.008293302

TMEM43	-0.764734702	0.008293302

AEBP2	-1.108132228	0.008298136

HAMP	1.763405808	0.008298136

STOX2	-1.308342238	0.008298136

ISOC2	0.991268554	0.008298136

MLLT11	1.532028332	0.008311171

ZW10	0.693740076	0.008319402

PTPN2	0.94320775	0.008349567

PLEKHA2	-0.772311884	0.008349567

RAD51AP1	1.983013218	0.008387649

PAM	-1.218679898	0.008387649

HMOX1	2.916214018	0.008415476

PPP2R5A	-0.854549038	0.008420419

TMEM5	0.77012377	0.008458404

PTGES2	0.883800174	0.008468505

IKBKE	1.74320345	0.008480506

SUPT6H	0.63065497	0.008480506

ELL2	1.839775	0.008480506

LOC650111	1.632821178	0.008497267

SPRYD4	0.919355866	0.008501582

C19orf61	0.975013036	0.008501582

GLTSCR2	-0.682450784	0.008508777

LOC642513	-0.914007508	0.008508777

RASGRP2	-1.724210938	0.008508777

SSU72	0.782490548	0.008511492

RECQL4	3.371218292	0.008511492

SLC22A3	-2.45711443	0.008512921

SEMA3B	-1.64417217	0.008520086

PCDHB4	-1.622725778	0.008530531

SPINT2	-2.00863309	0.008558918

ZBTB9	0.791710042	0.00859694

PYCR1	2.437325488	0.008621663

NIP7	1.08025685	0.008652487

THOC4	1.097493932	0.008696625

LOC727967	1.238430614	0.008696625

HSPB2	-1.249877094	0.008696625

SNORA3	2.609590612	0.008696625

VIPR1	-1.715261206	0.008700899

FAM181B	-2.076432188	0.008700899

NR3C1	-1.591437888	0.008700899

NOLA1	0.922923296	0.008703313

ATP6V0B	0.850351284	0.008715831

TSSC1	0.6411474	0.008715831

TEF	-1.996722972	0.008715831

LOC642452	1.539694682	0.008715831

INTU	-0.902831266	0.008715831

C13orf27	0.9468637	0.008733868

PPP2R1B	2.038506314	0.008733868

C14orf28	-1.49452756	0.008760305

RUNX1T1	-0.861737262	0.008760305

WDR34	1.66517996	0.008768223

ALG14	1.082817056	0.008785853

LOC650515	0.952873022	0.008791163

AKTIP	-0.814323416	0.008798546

REXO4	0.681343792	0.008801025

MRPS7	0.834447068	0.008804

ITGAE	0.701368398	0.008807297

GOLGA8B	-1.318831508	0.008822167

LOC651697	1.219362764	0.008822167

CCNB1	2.504771602	0.008822167

WWP2	-1.04199512	0.008843899

PHTF1	0.843419162	0.008845684

C22orf13	0.709646464	0.008856364

LOC100129566	0.72781251	0.0088724

MUT	-0.796263718	0.0088724

GLIS3	0.96381647	0.008875773

AAGAB	0.723418844	0.00888172

HEATR1	0.978673152	0.008921205

KIAA0391	0.724428534	0.008923906

TRAF7	1.398975096	0.008985592

G6PC3	1.050078674	0.009026175

OR4K13	1.309715574	0.009033986

RBM17	-0.734645548	0.009047395

EGFLAM	1.315203198	0.009060157

LMTK3	-1.335877868	0.009060157

P2RY11	1.467363996	0.009060157

OSBPL2	-1.016726636	0.009086326

HMGB3	2.63452822	0.009086326

TUBA1C	0.97198626	0.009086326

FKBPL	1.262289774	0.009124914

SRGAP1	1.486103844	0.009124914

SHRM	-1.795151602	0.009124914

TFPI	1.144417836	0.009135887

TMEM132A	1.957577364	0.009189336

TMED9	1.008699664	0.009190379

LOC390876	0.890795368	0.009197494

PDS5B	-0.69951132	0.009238636

ALG1	1.499838786	0.009238636

KIF22	0.991443318	0.009238636

APP	-0.915757806	0.009238636

RPUSD3	0.64838025	0.009238636

LYRM2	0.689487108	0.009260628

TACO1	0.59993646	0.009261754

RPUSD4	0.687249102	0.009261754

MIF	1.045686108	0.009261754

CDC14B	-1.152926298	0.009261754

LOC100132289	-0.997270832	0.009273563

TMEM39B	0.681274822	0.009273563

MAPT	-2.257050538	0.009273563

MRPL27	0.827325182	0.009293188

OSM	2.353560152	0.009306077

PIK3C2B	-1.132535656	0.009316926

KCNMA1	-1.796038494	0.009322745

NID2	2.729789708	0.00932326

SLC7A8	1.100391844	0.009331095

GNB5	-1.439124288	0.009331095

GCDH	0.720622652	0.009362301

NSUN7	-2.787094394	0.009370892

COPS3	0.662572806	0.009370892

DDX56	0.7916269	0.009391973

SLC25A19	1.04213173	0.009394913

PSMA5	0.628783206	0.009394913

LOC221710	1.55198197	0.009394913

TFPI2	2.378341334	0.009424495

HECTD2	-0.775312834	0.009425636

MMAA	1.026584642	0.009427301

LPP	-1.253120286	0.009427806

TWSG1	-1.263380198	0.009427806

PPP1R12C	-0.823444908	0.00942867

ASPM	4.409051698	0.009434315

SNRPD2	0.652654704	0.009434315

GLE1	0.632505052	0.009490048

C21orf70	1.048025244	0.009490048

SLC13A1	1.565366618	0.009501404

LOC653411	-1.45159633	0.009521797

BEND6	1.872681968	0.009574961

ALG5	0.747182482	0.009593181

DKC1	0.783082166	0.009600593

TRMT11	-1.038191808	0.009604747

FREQ	-0.970610484	0.009639077

FOXM1	3.351612226	0.009639077

ISCU	-0.810731894	0.009664612

POP1	1.334652924	0.009674412

FAM180A	-1.993892458	0.009678637

PSMA6	0.667730112	0.009701528

KCNB1	-1.813886226	0.009701528

NDRG3	-0.988337036	0.009732717

C5orf53	-1.334136116	0.009732717

TRAF2	1.589483562	0.009749128

C20orf3	0.95856884	0.009774905

LMBRD1	-0.992746978	0.009798164

PSMC3IP	1.201059972	0.009801679

HAND2	-1.449807918	0.009813172

EZH2	2.49034425	0.009829246

FAM110A	1.270367416	0.009832484

SERINC3	-0.69483626	0.009832484

HYOU1	1.03018195	0.009832484

ATP2A2	-0.820795546	0.009857485

MCAM	-1.800562398	0.009878336

POLD2	0.942998242	0.009917841

SNRPG	0.658062674	0.009956201

C12orf48	2.510849878	0.009976333

ATP6V0E2	-1.17411212	0.009976333

PSMD2	0.656214356	0.009976333

PPIL1	0.775248768	0.010000752

SERPINB8	1.578172912	0.010000752

ILF2	0.686280444	0.010000752

LOC100134393	1.143025588	0.010012464

ADRM1	0.699327044	0.010012464

RAPGEF2	-0.839645854	0.010012464

PTK2	-0.65632971	0.010012464

LOC731049	1.52509574	0.010016786

SHC4	-3.19533979	0.010016786

C14orf169	0.78224674	0.010016786

BEX4	-1.397652102	0.010016786

H2AFX	1.645849498	0.010017357

F2RL1	3.11610515	0.010017357

BCL2	-1.124426312	0.010018408

FEZ1	-0.861617916	0.010036188

LOC652696	1.910512912	0.010036188

CALM1	-0.816145754	0.010054594

PA2G4	0.870198742	0.010054594

LOC91431	1.57246246	0.010054594

BCAP29	-0.774100132	0.010054594

PARM1	-1.801494214	0.010054594

DAGLB	0.861265674	0.010054594

GINS3	1.614190468	0.010054594

SESTD1	-0.86888043	0.01010015

CROP	-0.91411367	0.010106597

ATIC	0.805216322	0.010125506

UBE2H	-0.941921004	0.01013058

BHLHE22	-2.743926588	0.010149429

LOC731314	1.70067052	0.010149429

ITGB4BP	0.737188352	0.010181986

LOC730278	1.127599962	0.010189474

C19orf33	-1.581661998	0.010194207

LOC100127885	-0.97143944	0.010204355

MATN2	-2.110258704	0.010239834

SMOC2	-2.046595916	0.010258651

TMEM111	0.70177835	0.010258651

PID1	-1.514029204	0.010263653

COG8	0.93215307	0.010263653

C8orf83	-0.855828324	0.010264047

ITM2A	-3.341076042	0.010276212

C8orf34	-3.492026472	0.010277537

BRI3BP	0.79804504	0.010285086

BEXL1	-1.093521824	0.010285086

LOC100130093	-1.740629062	0.010324423

SCRG1	-1.90462367	0.010349693

MIF4GD	-0.950059264	0.010355733

HGS	0.62619157	0.010381438

TP53I3	1.833631726	0.010390993

SF1	-0.976108428	0.010411064

PGD	1.65649035	0.010411064

GPC5	-1.531006546	0.010411646

C11orf82	2.532888226	0.010433643

MTMR11	-1.15724487	0.010480468

ACP2	0.84925434	0.010494077

RBBP8	1.251177454	0.010495838

KCTD5	0.68014816	0.010502973

PDGFD	-2.517858994	0.010502973

NOP56	1.065003374	0.010526967

DES	-3.209981446	0.010526967

TSGA14	1.181438212	0.010526967

UBE2C	4.266668182	0.010526967

MOAP1	-1.206736216	0.010542949

EPB41L5	-0.980317154	0.010542949

SF3B5	0.623848576	0.010542949

AHCY	0.819927956	0.010542949

FBXL18	0.634923674	0.010548913

MLPH	-2.53624656	0.010548913

CDK4	0.674287008	0.010548913

STIM1	-0.790565198	0.010548913

MSL3	0.813672338	0.010548913

DAG1	-1.070018178	0.01055359

LGALS2	-1.768293924	0.010571341

FLJ41603	-1.047694288	0.010575418

SGMS1	-1.113548424	0.010578279

SNX1	-1.055029604	0.010581312

TOMM22	0.721596256	0.010584321

TMEM208	0.778979568	0.010628321

C9orf3	-1.781556422	0.010634751

PISD	0.617285208	0.010642959

DDX39	1.20559769	0.010645374

MVD	0.81594795	0.010697641

PSIP1	-0.995743772	0.010741233

MAPKSP1	-0.940627142	0.010753245

XRRA1	0.983875406	0.010761403

LOC642282	1.598449078	0.010767277

PKD1	-1.276626764	0.010775388

KLHL23	-1.493066602	0.010778758

TP53	0.953217988	0.010778758

SLC38A10	1.181862444	0.01078398

KAZALD1	-1.55829849	0.01078398

PTBP1	0.77571242	0.01078398

LOC728666	0.902280728	0.01078398

COLEC11	-1.573293162	0.010802733

TPD52L1	-1.226333262	0.01081255

RPS6KA4	0.859043672	0.010820272

TIMP2	-1.050765738	0.010873341

ZNF322A	-0.627238302	0.010873341

SYNC	-1.93633398	0.010890555

LOC732165	1.37557246	0.010942732

CD68	1.027833284	0.010947791

TSC22D2	-0.688486392	0.010950023

ERO1L	0.822740008	0.010971736

ALKBH2	0.760708192	0.010971736

SLC39A1	0.830642284	0.011039935

SPNS1	0.906273502	0.011046516

LOC400506	1.088258774	0.011046516

TXNDC16	-1.458693014	0.011046516

GSR	0.822586894	0.011046516

FAHD1	0.84783476	0.011046516

SAT1	1.120347648	0.011046516

PROCR	1.688468698	0.011046516

SKP1A	-0.972949604	0.011046516

GAS6	-1.636337476	0.011046516

ABCF1	0.68607215	0.011046516

TOR1AIP1	-1.069444234	0.011085446

IPW	-2.074675784	0.011103693

GULP1	-2.074067572	0.011103693

FSTL4	1.44604521	0.011103693

TANC1	-1.078686826	0.011202519

SYT11	-1.19079234	0.011206638

CNPY2	0.695359728	0.011210277

NOP58	0.829337866	0.011212508

LOC727758	-1.180832336	0.011212508

LOC100133697	1.397510002	0.011212508

MGAT2	0.982577344	0.011212508

WWC3	-0.963613364	0.011212508

SGPL1	0.846562052	0.011212508

RIMKLB	-0.98658982	0.011212508

NDUFA9	0.55392267	0.011248987

PLS3	-1.028534848	0.011262623

LOC440311	-1.390465386	0.011306871

LOC100130886	0.640984894	0.011309679

WBP4	-0.837674222	0.011309679

CENPK	1.9408705	0.01131774

ELOF1	0.689733158	0.01131774

C17orf108	-1.283441148	0.011329385

CYBRD1	-1.344388172	0.011332229

NAB1	-0.684111522	0.011385806

KPNA2	1.863155234	0.011385806

SFRS2IP	-0.599160758	0.011385806

SYT15	-0.98509073	0.011385806

SNRNP40	0.656877172	0.011398424

WDR57	0.786885992	0.011458677

CTSL1	1.19387	0.011458677

C20orf177	-1.147123496	0.011479598

PAPSS2	1.286883528	0.011482982

RMI1	1.152927108	0.011496731

GCAT	1.540921446	0.011496731

LOC441377	1.12134139	0.011498634

PCMTD1	-0.866280044	0.011503435

C1QBP	0.920284156	0.011503435

N6AMT1	-0.981566056	0.011513746

LOC391532	0.72127452	0.011516208

CCNA2	2.378201706	0.011516208

FAH	0.869215116	0.011516208

NME1-NME2	0.79845211	0.011516208

HNRPH3	-0.850694224	0.011516208

C7orf68	2.2106592	0.011516208

LOC728635	1.21204283	0.01151751

MARK1	-1.36611603	0.011537511

CNTN3	-2.080805838	0.011537849

GAD1	-2.060756734	0.011537849

ANKRD33	1.300841568	0.011564662

DMD	-2.741152874	0.011599367

PCNP	-0.823615262	0.011599367

RRM1	0.93584339	0.011599367

LOC100134584	-1.13146773	0.011599367

SYNPO2	-1.812801654	0.011599367

ITGA9	-1.151469448	0.011649501

SLC38A7	1.168692802	0.011700912

NUP93	0.968631408	0.01170791

ANKMY2	-1.01634644	0.011709811

C9orf100	1.164323948	0.011711704

EFTUD1	0.671691894	0.011713608

N6AMT2	0.615250736	0.011713608

RARS	0.601196282	0.011736068

COPG2	0.884956026	0.011749577

PLK4	2.470478494	0.011749577

YES1	0.721255876	0.011753636

RGAG4	-1.533228904	0.011753636

AHNAK	-0.842294164	0.011753636

GLA	0.821777516	0.011753636

C21orf81	-1.8559604	0.011784183

TTC38	0.741264966	0.011784183

OMD	-2.313579484	0.011784183

UTP11L	0.781368148	0.011784183

NR1D2	-1.39766472	0.011784183

LOC653874	1.370542638	0.011786498

C6orf115	0.903844514	0.011801437

CYP4X1	-2.54311166	0.011827938

PDE1B	0.849847012	0.011842899

CRISPLD1	-2.564924388	0.011870163

SOD2	2.442397938	0.011895722

DIDO1	-1.015271158	0.011914462

NR2F2	-0.939016604	0.011944419

RGS11	-1.324574602	0.011944419

EXOC6	-0.645791954	0.011944419

ZDHHC24	1.373372464	0.011992818

FARS2	0.612521316	0.012003492

ATP1A1	0.795389236	0.012003492

CARD6	1.890504008	0.012014824

C10orf10	2.057228468	0.012014824

ASCC3	0.886167136	0.012014824

C12orf44	0.786942508	0.012014824

STEAP3	1.282373182	0.012014824

C3orf21	1.018787696	0.012014824

DEPDC1B	2.22569867	0.012022565

MFSD5	0.780481902	0.012022565

LOC100133866	-1.276517226	0.012022565

FAM86A	1.064816822	0.012022565

SLC25A36	-0.855791598	0.012034525

SPA17	1.086303652	0.012034525

ANGPT1	-1.8082137	0.012034525

ANKHD1	-0.745386428	0.012042891

ABLIM2	-1.788211416	0.012042891

TMEM180	0.882253896	0.012077527

SMUG1	0.626460444	0.012091939

CASP4	0.927046176	0.012139122

SNTA1	-1.010050238	0.012139122

COL8A2	-1.528664656	0.012154536

IDH3A	0.994764702	0.012154536

MCM5	1.787647256	0.012154536

GPRASP2	-0.952517192	0.012172054

NXN	-1.189650114	0.012172054

BUB3	0.636445744	0.012172054

SNORA65	1.317023042	0.012194415

LOC645937	1.03827792	0.012217624

SEMA4B	1.378379892	0.012217624

ICOSLG	1.203312648	0.012248714

TNFRSF10D	2.172122086	0.012248714

ANXA3	-2.142929174	0.012274258

FGFR1	-1.117681742	0.012274258

SNHG1	0.79731306	0.012274258

LOC92249	-1.0532845	0.012274258

PRG2	1.058432342	0.012303966

YPEL5	-0.982172064	0.012312362

PSRC1	1.790930862	0.012315932

FAM129A	-1.355598096	0.012341469

CDKN1B	-1.018358136	0.012367114

ATP1B2	-1.793698296	0.012367114

RAP1A	-0.915709794	0.012367114

ABLIM1	-1.149567118	0.012367114

DHX8	0.647983688	0.012397937

LOC401127	0.976588674	0.012411422

PSMD1	0.52408179	0.0124139

C19orf10	1.74297481	0.012451847

TBRG4	0.992456082	0.012460378

CHN2	-1.999710916	0.012502186

LIG1	1.419483734	0.012502186

SEPP1	-3.495366508	0.012506677

CARS	0.85183263	0.012508694

TTLL12	1.278120056	0.012508694

CBX6	-0.730723932	0.012508694

LOC85389	1.141752196	0.012525093

RCADH5	-1.432769288	0.01255827

ABCE1	0.871601836	0.012575969

B4GALNT1	2.003795386	0.012596938

XYLB	1.642378968	0.012596938

ZBTB4	-0.788083766	0.012608424

SP4	-1.080058802	0.012617581

MYOZ1	-2.558089304	0.012617581

SEC61B	0.657093288	0.012618598

OPRK1	-1.32973657	0.012618598

TXLNA	0.593927996	0.012618598

RPA3	0.990059654	0.012618598

BSG	0.706733962	0.012637032

LOC400464	-1.001447246	0.012641638

IGSF9B	-1.923464546	0.012641638

TOP1MT	0.79434593	0.012663728

SSBP4	0.73826569	0.012663728

NRBF2	0.992148288	0.012664632

PEX13	0.963773844	0.012664632

RPN2	0.898189034	0.012684699

BTLA	-2.11889156	0.012695796

ASTN2	-1.466929014	0.012712211

ARHGEF12	-0.702371058	0.012712211

ADAT3	1.084355424	0.012712211

OLR1	3.62909197	0.012712211

DSN1	1.132413574	0.012712211

DPM2	0.914955404	0.012736245

C4orf46	0.920576416	0.012752267

SERPINA1	2.323506014	0.01280617

NTN4	-1.156184914	0.01280617

COQ3	0.700637062	0.012810177

FREM1	-3.098126638	0.012895446

PAFAH1B3	1.89106882	0.012899029

MRPS15	0.6004765	0.012899029

C2orf29	0.613475306	0.012899029

BMP4	-1.402428598	0.012920654

DNAL1	1.030079048	0.012926071

MID1	-0.848133246	0.012935704

TRMT1	0.893395134	0.012935704

LOC642197	0.92225458	0.012942792

GDF10	-1.94677277	0.01300894

C16orf75	1.336938032	0.013020842

WSB1	-0.906199168	0.013020842

LEPR	-1.553225568	0.013046029

ZNF235	-1.367606076	0.013105113

IFRD2	1.001796502	0.013115341

LOC729608	0.835275478	0.013153152

ABCC4	0.845313348	0.013162149

EIF4G1	0.893807804	0.013162149

ELF2	-0.803385738	0.013176345

SENP6	-0.635586548	0.013176677

NDUFAF2	0.68005287	0.013197283

RAMP1	-2.145359918	0.013203659

MRAP2	-2.227263576	0.013203659

COL4A1	0.806924344	0.013209485

C16orf61	0.602156558	0.013209485

CCT2	0.911471138	0.013209485

SCHIP1	-1.15798142	0.013209485

FCGR1A	1.844974964	0.013209485

SLC24A3	-1.844403048	0.013209485

ARHGAP10	-1.213254996	0.013239378

ABCF2	0.724499286	0.013246758

EIF6	0.664539684	0.013263845

LOC647346	-1.292512008	0.01327683

LOC646567	0.942261204	0.013313111

LYST	-1.131272378	0.013326057

AZI2	-0.661684842	0.013334394

MOBKL2A	0.771439042	0.013397972

YPEL2	-0.999704604	0.013439333

THUMPD2	-1.331482716	0.013439333

MGC39900	-1.069465158	0.013439333

RELB	0.982964854	0.013458367

FLJ90650	-1.865381494	0.01348027

CAPN7	-0.757229412	0.013494574

MAPK13	1.826080578	0.013525398

TPX2	3.23609603	0.013525398

SPOP	-0.839644876	0.013525398

KATNB1	0.848157826	0.013542277

LAMB1	1.18932283	0.013542277

PPM1H	-1.347021956	0.013542277

BMS1	0.880000388	0.013542277

LOC441484	0.652934836	0.013542277

UNQ1940	-2.111384378	0.013542277

LRRC20	1.037085954	0.013542277

FBXL2	-1.156706042	0.013582164

LOC441136	1.572897522	0.013582398

WEE1	-0.813080842	0.013599022

COMMD9	0.84838505	0.013600196

KIAA0907	-0.55871669	0.013611589

ZBTB8OS	0.731475462	0.013619611

NUP155	0.82911584	0.013684552

SNORA73B	1.919926978	0.013684552

OLFML2A	-2.491864978	0.013684552

KCNAB1	-3.241440354	0.013684552

LOC400455	0.797701356	0.013684552

LOC645979	1.133059166	0.013697397

VAMP2	-1.040128404	0.013697397

LOC390578	1.275048076	0.013697397

LOC728257	-1.75558846	0.013736181

RNF19A	-0.78049816	0.013787381

SERPINA3	2.89151544	0.013815303

LOC389662	-0.86359694	0.013864322

BPTF	-0.680868144	0.013894074

C6orf117	-2.199748158	0.013921537

LOC648749	-1.179671392	0.013939612

TMEM48	1.072606972	0.013939612

WDR67	0.871707214	0.013940154

SRXN1	0.960565538	0.013959556

NCRNA00095	0.885296576	0.013973531

CA12	2.378625634	0.013973531

LOC643841	-1.842113466	0.014012697

APEH	0.660798552	0.014013418

POLA1	0.809257762	0.014027744

KLF12	-0.928433248	0.014027744

MMP23A	-1.77871518	0.014064721

POLR1C	0.843787552	0.014064721

ZEB1	-1.405379492	0.014064721

WARS	0.844582462	0.014064721

LOC647531	1.815604888	0.014064721

TAF4B	1.173040904	0.014064721

ZFHX3	-0.943965618	0.014064721

ACAT1	-0.692656084	0.014093614

MRPL35	0.594441932	0.014095132

AMY2A	-1.03471944	0.014095132

BAZ1A	0.831543532	0.014095132

SLC39A7	1.052471224	0.014095132

DBNDD1	-1.6403425	0.014103449

RBM28	0.81699085	0.014132275

TCEB3CL	2.416552838	0.014132275

IL8	3.866336006	0.014137206

LOC728263	0.935509688	0.014229237

ZNF10	-1.275630258	0.014233889

APEX1	0.641798796	0.014241548

FRAG1	0.64291218	0.014253216

AFAP1L2	-1.7728964	0.014260714

DHRS4	0.7431283	0.014279411

RNF141	-1.047665626	0.01429487

DAAM2	-1.152902662	0.014304412

RPESP	-2.066134606	0.01434776

ZNF704	-2.388076012	0.014365988

TSPAN2	-2.942532632	0.014368549

LOC728564	0.787226718	0.014368549

PREB	0.845899772	0.014368549

WDR77	0.69584924	0.014368549

LOC728531	-1.42770299	0.014377614

FBXL6	0.653793084	0.014399682

KIAA1949	0.70583781	0.014402042

LOC650200	-1.837256542	0.014404891

SLC4A2	0.910835956	0.014458747

TNRC6B	-0.665237136	0.014458747

CLPTM1L	0.681204944	0.014464033

H2AFY2	0.950421694	0.014464033

HSPA12A	-1.00310754	0.014464033

LDHA	0.854220102	0.014464033

BVES	-1.771208826	0.014464033

MYH11	-2.149770548	0.014504409

LOC730273	-0.997693206	0.014509824

DHDDS	0.81346867	0.014509824

DIP2C	-1.187919708	0.014536989

MTIF2	0.565466376	0.014536989

SPEN	-0.726749214	0.014538732

PRDX4	0.89160894	0.014672726

FAM46B	-1.826049454	0.014672726

DNAJB5	-1.275498584	0.014680081

CTSF	-1.33635692	0.014680081

KHSRP	1.067669578	0.014680081

ATP6V0A2	0.51578325	0.014680081

GCNT2	-1.574561592	0.014683082

SNORA67	1.299972484	0.014723761

GTF3C6	0.835334824	0.014729292

MGC102966	2.201236428	0.014729292

C6orf111	-0.902790096	0.014729292

ALPK1	0.838521448	0.014729292

THG1L	1.907488702	0.014745798

TRIM27	0.774434656	0.01477612

COL4A3	-3.26840615	0.01477612

MAFB	1.089506156	0.014784853

TSFM	0.76698121	0.014812774

USE1	0.900447774	0.014845288

GAB1	-1.307370442	0.014845288

ELOVL3	1.11995839	0.014900786

RPL35	0.558389794	0.014945853

RDBP	0.833869122	0.014948361

MAP2	-1.294667296	0.014954811

INADL	-1.393910932	0.014954811

LOC645644	1.4079241	0.014975659

KIAA0664	1.014573646	0.014975659

MGC71805	-0.86015067	0.014987277

METTL13	0.710173594	0.014993557

UBA2	-1.143965878	0.014993557

RASL12	-1.478634564	0.015008128

MMP23B	-1.839511404	0.015010495

MEGF6	-1.917890308	0.01502182

HSDL2	-0.920777554	0.015033477

TGFBI	1.296880744	0.015085332

SNORA33	1.13029603	0.015085332

KIAA2026	-0.773693484	0.015085332

LOC391655	1.593676518	0.015090811

USP33	-0.690423994	0.015091234

LOC649076	0.530723866	0.015091234

TFPT	0.654945764	0.015107439

DNAJC9	0.863017146	0.015118242

ZNF219	-0.730685468	0.015118242

CRELD2	0.961336084	0.015129244

NUDT6	0.638411678	0.015174663

PDSS2	0.772199638	0.015174663

SNRNP200	-0.974367108	0.01519465

GOLGA8A	-1.428633616	0.015217406

FTHL11	0.882731598	0.015230007

OSTC	0.93385132	0.01527343

LOC644214	1.029167404	0.015339014

LGR6	-2.445357468	0.015344199

TTC14	-1.08435608	0.01535622

IGSF3	-1.288375774	0.015407765

FERMT2	-0.728465576	0.015434637

USF1	0.873372028	0.015439223

DOCK3	-1.069100032	0.015471865

NOC4L	0.694158722	0.015486199

LIMK2	1.344142042	0.015486489

PSMC5	0.628858072	0.015488097

LOC341784	1.055768516	0.015527141

RRS1	1.134545726	0.015527141

C15orf39	0.841465184	0.015535877

PTPN11	-0.727647498	0.015535877

HSP90B1	0.870495998	0.015535877

PVR	0.96553238	0.015535877

KIF15	2.453643308	0.015535877

HSPA2	-1.980784924	0.015535877

DDOST	1.155404468	0.015553054

OXR1	-1.028326046	0.015554368

DKFZp547K054	-0.921853186	0.015560548

PRRT2	-1.907338996	0.015585064

EXOSC3	0.696546944	0.015591591

C21orf122	-1.181634946	0.015591591

RER1	0.63934245	0.015591591

CCDC128	-0.685697926	0.015591591

SH3GLB1	0.774641046	0.015591591

MAGEE1	-0.90102547	0.015591591

MAPK4	-2.409447448	0.015591591

FAM84B	-3.14548851	0.015591591

ABL2	1.908644086	0.015591591

XPO6	0.776734088	0.015591591

LOC728556	0.67720424	0.015591591

CLK4	-1.427270782	0.015591591

H2AFY	0.62516693	0.015591591

FAM63B	-1.36496223	0.015591591

LSM4	0.952516868	0.015591591

VPS16	0.628740398	0.015591591

STAG3L1	-1.160884008	0.01560849

C9orf61	-1.107455432	0.015612648

SPTBN1	-0.951437326	0.015615516

LOC729260	0.671745174	0.015643709

NCKIPSD	-0.898021166	0.01564666

RPAIN	-0.620062324	0.015662065

LOC731751	0.923020462	0.015662065

C10orf65	-1.736836206	0.015662065

PLEKHA1	-0.6007505	0.015662065

LOC92755	1.075652122	0.015662065

KIAA1324L	-2.030384488	0.015662065

LOC651073	-1.232634942	0.015662065

RAP1GDS1	0.688944938	0.015662065

LOC91664	-0.916100688	0.015678387

KIF11	3.2351616	0.015688083

BEND7	-0.94537431	0.015747192

SSH3	-1.087866832	0.015766507

LOC100134587	-1.915482238	0.015788211

ICT1	0.646943686	0.015801205

INO80B	0.745478076	0.015802886

ARMC6	1.042676656	0.015802886

LOC645241	-0.7645706	0.015803139

LOC440043	0.594975786	0.015830961

WSB2	0.870423042	0.015832379

NAGPA	0.805664176	0.015837055

ALDH6A1	-1.105785338	0.015955105

LOC652624	0.625113526	0.015955105

DNAJC5	0.828466288	0.015965615

TRIM56	-0.880353874	0.016020462

CALCOCO1	-1.44542638	0.016077999

ZC3HC1	0.550674826	0.016107189

WDR55	0.763996628	0.016149219

SRPK1	0.675643192	0.016149219

HOMER3	1.379934086	0.016149219

TRIM23	-1.11261699	0.016149219

GABPA	-0.9003107	0.016149219

SMARCA4	0.72069086	0.016149219

MLL5	-0.723521898	0.016150207

MYADM	-0.788706298	0.016153148

C10orf11	-1.040338948	0.016173691

TAPT1	-0.938389648	0.016173691

RBM20	-1.913958494	0.016173997

ZNF443	-0.585650228	0.016180673

COMMD4	0.81070846	0.016188571

LIN52	-0.601819388	0.016188829

LILRA6	2.152163656	0.016188829

C19orf53	0.512911742	0.016204532

RNF113A	0.566716804	0.016205574

LOC100130154	1.176521178	0.016234652

TFAP4	1.488596568	0.016281294

PDLIM3	-1.622209224	0.016294546

SP100	0.801226102	0.016300145

RFC3	1.216292814	0.016317233

GLT25D2	-2.418461134	0.016317233

THADA	0.64274107	0.016317233

FADD	0.74962873	0.016320159

SNRPA1	0.638963852	0.01632665

CEBPD	1.431576486	0.016337582

LOC400027	-0.706960708	0.016378815

PMPCA	0.77148192	0.016444982

PXMP2	-0.923210216	0.016459969

TAGLN2	0.978356492	0.016469125

RAVER2	-1.17473355	0.016475348

MSH6	0.551109946	0.016475348

F10	-2.49199002	0.016501232

LOC100134304	1.058013398	0.016516247

MRVI1	-2.124010832	0.016516247

ERCC1	0.69931562	0.016516247

LSM12	0.622953254	0.016516247

SCXA	-2.153894182	0.016516247

PGR	-2.200536034	0.016523129

LOC648740	1.086826514	0.016524091

KDELR2	1.278768916	0.016563965

SH3GL1	0.798944654	0.016563965

TCEAL2	-1.840678982	0.016563965

C19orf22	1.132603498	0.016563965

FBXL7	-0.897548956	0.016598945

SMARCC2	-0.764477926	0.016628248

ATP5D	0.56170994	0.016661884

DAZAP1	0.796892348	0.016661884

NPC1L1	-2.131488924	0.016661884

CSK	0.924818988	0.016661884

ERI1	0.977262818	0.016666103

C12orf32	0.79596678	0.016692048

LOC128192	1.042005628	0.016692048

NCALD	-1.496839142	0.016692048

HSD17B14	-1.276660392	0.016692499

ZFPM2	-1.042865274	0.016692499

TSPAN9	-0.506043194	0.016748792

LOC646817	1.1054182	0.016749533

ZNF598	0.711562886	0.016753118

PATE3	1.548350244	0.016794174

TBC1D7	0.990764524	0.016794174

SNAPC4	0.796325386	0.016822335

OSMR	1.513071348	0.016830663

SPARC	-1.11944205	0.016830663

CABLES1	-1.02474057	0.01684258

MAPBPIP	0.7449431	0.01684258

FLNA	-1.208298158	0.016849548

MRPL36	0.582033636	0.016855126

C11orf70	1.100038632	0.016855126

NUP50	1.747751696	0.016855126

PGAM5	1.015345074	0.016855126

RASD2	-2.533680208	0.016855126

GPBP1	-0.55748318	0.016855126

FXYD1	-1.930217002	0.016855126

BBS2	-0.735107134	0.016877513

C16orf57	0.660822492	0.016881523

STRN3	-0.823602514	0.01692542

FAM122A	-1.079371442	0.01692542

PAFAH1B1	-0.622512272	0.016941124

NDP	2.372130278	0.01697506

GNAI1	-1.146578044	0.016992929

TERF1	-1.42927872	0.016992929

MYO6	-0.634350236	0.016992929

GGT3	1.242257956	0.01702664

CLIP4	-1.21090915	0.017032641

DST	-1.113247176	0.017035352

NTN1	-1.66122352	0.017056167

LOC643778	-1.36464018	0.017057231

PLEKHA7	-1.357202448	0.017057231

NDUFS4	-0.651089816	0.017163209

RTCD1	0.704381498	0.017168822

TCEAL1	-1.337942646	0.017173483

CHSY3	-1.04699316	0.01720779

C4orf48	2.41379721	0.017221382

LOC729680	-1.125173828	0.017221382

C7orf20	0.720427988	0.017221382

PCYOX1	-1.08840342	0.017241861

LOC732150	1.887743966	0.017290228

CAPZA1	0.88942878	0.017292289

POGZ	-1.000468412	0.017302719

TMEM183B	0.624507782	0.017310674

RRBP1	0.831578836	0.017319781

C8orf30B	1.896429714	0.017322572

SLC43A3	1.152223804	0.017322572

MCM3	0.830084466	0.017333811

B4GALT1	0.8667998	0.017333811

PHACTR4	-0.518505662	0.017359885

PPP1R14B	1.020828582	0.017381954

KPNB1	0.644130664	0.017395771

SCPEP1	-0.912945526	0.017422447

SFRP4	-2.085833888	0.017435085

TMEM18	-0.730430844	0.017456738

TPM2	-0.822104038	0.017467083

LOC649445	0.646027148	0.017467083

NPAS2	1.02931621	0.017476482

CHCHD5	0.785842338	0.017476482

TNFRSF11B	-2.065289882	0.017478093

ZBTB40	-1.09744823	0.017508774

HNRNPH3	-0.750305372	0.017535599

TOMM40L	0.930033012	0.017535599

CHTF18	1.511194878	0.01756371

FAM195A	0.993604022	0.01756371

ZNF295	-1.042039582	0.017621403

TXN	0.639465232	0.017626739

APEX2	0.91120272	0.017626739

SEC61G	0.673138634	0.017626739

AKAP11	-0.632376448	0.017626739

METAP1	-0.615086086	0.017644434

MGC16384	-1.35713431	0.017649375

PSAT1	1.263898644	0.017656859

TUBG2	-0.95968511	0.017665791

LOC643007	0.921504684	0.01767061

AGPAT5	1.030283866	0.017687153

TGFB1I1	-0.883204226	0.017704353

ZNF654	-0.772288446	0.017715408

WDR25	0.848995102	0.017715408

TBC1D22A	0.83180127	0.017715408

MPV17L2	1.211736082	0.017715408

TUFM	0.83688489	0.017715408

MEIS3	0.800440308	0.017751595

ACADM	-0.600732174	0.017769308

C11orf58	-0.675323674	0.01778361

RHOQ	-0.621749808	0.017808004

FZD4	-0.712412754	0.017828003

MKLN1	-0.65034107	0.017857683

LOC100133489	1.361546144	0.017857683

FASTKD1	0.701442918	0.017857683

C1orf21	-1.956939914	0.017858285

PSMA4	0.608456744	0.01790068

FRYL	-0.54495587	0.017913927

MS4A2	-2.389336256	0.017913927

KIAA0363	-1.17760284	0.017913927

PTRH2	0.83671527	0.017913927

ECM2	-2.396733842	0.017913927

SOX12	-1.273080108	0.017934163

UBE1	0.929008218	0.017990929

PPP1R3E	-0.658177854	0.017994325

FAM113B	-1.031226032	0.018004368

LOC729200	0.557035982	0.018004368

ZNF680	-0.636918434	0.018009215

RPF2	0.708150876	0.018013912

MIB1	-1.103381018	0.018013912

MRI1	-0.789738762	0.018077439

FOXC1	-1.097769748	0.018120174

BCKDK	0.586259684	0.018142139

PABPC4	0.61884895	0.018142139

DHX35	-0.874088958	0.0182006

LOC134997	0.74777887	0.01820451

LOC648024	1.19258737	0.018262975

WISP1	1.494591772	0.01826605

ASMTL	-0.533339742	0.018266542

ZNRF3	-1.094828022	0.018278495

DNAJB4	-1.52249132	0.018292348

PNPLA6	0.82242283	0.018302345

TUFT1	-1.133752336	0.018302345

PHC2	1.062221248	0.018325419

ZNF529	-0.771465062	0.018325419

RFTN1	-0.781979012	0.018325419

PNKP	0.855178718	0.018325419

LOC731724	-1.132487576	0.018325419

CHEK2	1.768481818	0.018325419

C9orf46	0.643862544	0.018329727

SNORA18	0.686564012	0.018340858

DPH2	0.709652808	0.018340858

KNTC1	1.302843258	0.018340858

LOC100134424	1.02866149	0.018355726

TOMM5	0.77843282	0.018380292

PRKAR1A	-0.84960308	0.018380292

SMG6	0.912343622	0.018416667

MCM7	0.94772777	0.018431139

SNRPD3	-0.700207092	0.01843881

LOC643888	-1.664828112	0.01843881

NET1	-1.51857092	0.018544154

MMP28	-1.839952132	0.018548865

AATF	0.82927582	0.018550903

OBFC2B	0.917745428	0.018562076

GK5	-1.093087608	0.018582205

PFN2	-1.151245286	0.018598524

ECGF1	1.935674556	0.018606546

TMED1	0.594214922	0.018661364

LOC648434	-0.91820067	0.018686614

RSBN1L	-0.782062638	0.018690106

MGAT4A	1.166267676	0.018690106

SEC61A1	0.897489758	0.018771891

INTS5	0.776679618	0.018771891

ARF3	0.68821986	0.018807838

TRIM33	-0.53792085	0.018807838

SLMO1	0.757652648	0.018807838

LOC644353	-1.22727319	0.018807838

PRPSAP1	-0.68386751	0.018810254

JMJD1C	-0.576876714	0.018838196

TACC1	-0.67648678	0.018851745

GALNT11	-0.565043502	0.018884744

OSBPL9	-0.747806656	0.018907022

MED30	-0.971337534	0.018907022

SMARCD1	0.568117382	0.018907022

RASL11A	-1.107973804	0.018914769

MIR586	1.087473162	0.018914769

HSPB6	-1.169990084	0.018914769

CBFA2T2	-0.757754588	0.018914769

CPXM1	1.66860577	0.018914769

LPPR2	0.752208846	0.018914769

PDZK1IP1	2.844510488	0.018958656

HERC3	-0.957550404	0.018958656

CENPE	2.50494339	0.018958656

LDLR	1.31300438	0.018967884

TMEM132B	-1.403777424	0.018975265

ZNF787	0.910793018	0.019006829

LMCD1	-1.325788232	0.019006829

GAP43	1.537675346	0.019006829

PIGW	1.370003274	0.019006829

LRIG3	-1.766348918	0.019006829

CTGF	-1.089905116	0.019006829

LOC730029	0.62455789	0.019037772

PDLIM1	-0.63643208	0.019042457

EIF2C4	-0.985872222	0.019052387

CCDC138	1.11232779	0.019057261

GLMN	0.598010724	0.019057261

ZNF789	-0.873272016	0.019121656

FTHL12	0.815757154	0.019201589

LETM1	1.287640626	0.019231538

LOC644799	-0.79089577	0.019244854

COX10	0.686266308	0.019258602

RNF185	-0.846532892	0.019285641

C11orf46	-0.96129035	0.019286708

C20orf72	0.712565716	0.019351215

GADD45B	1.762884696	0.019408953

MYO9A	-0.66712709	0.019483496

HSZFP36	-0.639551302	0.019487389

FIBIN	-1.766841804	0.019509968

CCNB1IP1	-0.66423927	0.019531272

MORN2	1.096311954	0.019534996

TMEM147	0.578037516	0.019571473

CRBN	-0.908217632	0.019581705

LOC728554	0.808159148	0.01964497

LY6H	2.07704462	0.0197005

CPE	-1.404075304	0.0197005

BEX2	-2.49007268	0.019735766

RAB18	-1.126223774	0.019735766

LOC283953	0.832544182	0.019735766

RCOR3	-0.856918088	0.019735766

MCCC1	-0.57215999	0.019735766

C13orf34	1.279811214	0.019735766

METTL7B	2.30522388	0.019735766

PSMC1	0.523861274	0.019735766

ELK1	0.782634966	0.019738037

PPM1G	0.626752484	0.019738037

ATG5	-0.640287822	0.019748027

LOC151162	0.590583098	0.019748027

PIK3C2A	-1.009311456	0.019748027

C13orf29	1.4631214	0.019748027

PNRC2	-0.665874878	0.019827214

SMAD5	-0.533359886	0.019829053

SLC25A46	0.575382988	0.019831183

TMEM38A	-1.61964163	0.01986303

SURF4	0.89785131	0.01990443

SCAMP3	0.751635458	0.019920143

PMEPA1	-1.110836768	0.019920387

BAX	1.194618278	0.019920387

PRDM16	-1.600758038	0.019949186

LOC653463	1.225677132	0.019949186

STAG2	-0.725165592	0.019967711

LOC100132992	0.718774704	0.019968937

LOC401115	1.746545294	0.020021102

LOC653162	0.557743076	0.020057509

ABCB4	-1.863014244	0.020090539

MEF2A	-1.036384882	0.02015227

SHKBP1	0.886728452	0.020163521

LOC653472	-1.713605788	0.020228796

PHLPP2	-0.636397458	0.020248024

ESD	-0.658805202	0.020248625

C22orf39	-1.082098026	0.020277116

FBXL3	-1.278115226	0.020277116

UTP14C	-0.749177396	0.020326387

FH	0.622277244	0.020366646

HPRT1	0.538395628	0.020366646

C14orf133	0.84625961	0.020366646

GZMK	-3.08601585	0.020470917

LOC92659	1.105683168	0.020496816

LOC645175	0.970949546	0.020500341

TBC1D10B	0.892907662	0.020500341

EMR2	1.88179878	0.020508702

YTHDC1	-0.582992374	0.020511853

MOCOS	2.49675868	0.020612674

PLIN2	1.512734726	0.020612674

PHF20	-0.545430664	0.020612674

LOC644994	1.571661174	0.020613007

LOC651212	-0.991352126	0.020613007

FDPS	0.688399584	0.020613007

C14orf79	1.053309502	0.020613007

CDH20	-1.218469474	0.020632929

VPS13A	-1.101170378	0.020632929

ZNF518A	-0.719147498	0.020654854

PCF11	-0.62392873	0.020655788

APCDD1L	2.234532092	0.020668353

CRYZL1	-0.922783398	0.020675958

BCL6	0.859134334	0.020688968

LRCH2	-0.896305392	0.020688968

TXNL1	0.66329649	0.020714259

C5orf46	-2.241309248	0.020756415

RHOT1	-0.529122074	0.020758381

CYB561	0.953155588	0.020799916

LOC728734	-0.606042008	0.020799916

COQ2	1.203214504	0.020799916

KLK1	-1.564180784	0.020799916

DHTKD1	-0.65509222	0.020799916

SUPV3L1	0.532662858	0.020804171

ZNF493	-0.861692336	0.020804171

USP6NL	-0.636189202	0.020811762

UTP14A	0.566611328	0.02081476

ZGPAT	0.625393886	0.02081476

EME1	2.656886598	0.020973373

LOC729009	0.909038828	0.021027411

FAM109A	0.779002956	0.021033782

C12orf76	-0.955694836	0.021067082

NIPA2	0.687277416	0.02106969

LOC728823	1.031353942	0.021107969

PCDH7	-1.488549938	0.021107969

SGSM1	-1.199988864	0.021107969

FLJ25006	-0.85544887	0.021107969

GADD45GIP1	0.9362485	0.02112536

PRELP	-2.19604932	0.021176608

DNMT1	0.869005536	0.021212411

RERE	-0.595613636	0.02122987

PLEKHH2	-1.636239038	0.021233081

EBNA1BP2	0.75637567	0.021233806

MTA2	0.63404121	0.021233806

TERF2IP	-0.672422012	0.021233806

FTHL3	0.90435417	0.021238323

TSPAN7	-3.256896166	0.0213087

FAM27A	2.969100396	0.021314365

RECK	-0.906666028	0.021314365

CCNI	-0.676108334	0.021314365

LOC100129499	-1.072088142	0.021314365

BEX5	-1.193453938	0.021348526

MRPS11	0.54051686	0.021348526

ARGLU1	-0.70783231	0.021386093

LOC652097	1.228613276	0.021396161

CREB5	-2.177951428	0.021396161

SNORD15B	1.172411354	0.021396161

LOC442597	1.947803766	0.021439011

ANKRD50	-0.884478454	0.02144235

LOC401010	1.28543837	0.021461347

PLEKHF1	-0.530696722	0.021481409

PHF23	0.759970342	0.021481409

DDX28	0.675045772	0.021512827

VAMP4	-0.937408502	0.021623402

CPSF6	-0.76834221	0.021669435

LOC343052	1.284181784	0.02167637

RFX5	-0.561627424	0.021724778

PRPF19	0.830795244	0.021732158

NUDT1	0.810588876	0.021756327

FPR1	2.214069854	0.021791986

MAMDC2	-2.896691574	0.021791986

MT1L	2.03619309	0.021791986

PRDM2	-0.924006808	0.021831486

SUB1	-0.984228242	0.021983751

PSME2	0.661717794	0.021983751

FAM87A	2.140582398	0.022016157

LOC650368	1.039539002	0.022042709

KCTD12	-1.010501878	0.022042709

TES	-0.833157958	0.022061446

HK3	2.890401766	0.022061446

DCAF16	-0.93364635	0.022147484

ZNF451	-0.713467092	0.022147484

RAB22A	-0.783907168	0.02219122

EXOG	0.828177252	0.022221586

HABP4	-1.176193808	0.022238442

PPP1R3C	-1.586327904	0.022280537

C2orf7	0.732709028	0.022332548

PAPD4	-0.52642353	0.0223409

DCTN5	0.605859882	0.0223409

PITRM1	0.75583594	0.0223409

PDZRN4	-2.256821664	0.022369766

GPC4	-1.701199256	0.022399691

MAPKAPK2	0.622003626	0.022399691

UBE2I	0.634507526	0.022432228

L3MBTL	-1.09324919	0.022503204

SMAD3	0.802116282	0.022528384

KPNA1	0.639153664	0.02253193

FAM13AOS	-2.008174698	0.022586472

NUSAP1	2.962734668	0.022590468

SHPRH	-0.793046738	0.022590468

PARP1	0.736279688	0.022590777

FLJ22639	-2.288780066	0.022628398

ERCC4	-0.928491678	0.022628398

RCN1	0.690023208	0.022645688

SKP1	-0.950237462	0.022686834

PJA2	-0.615101124	0.022686834

ABCA11	-1.810176416	0.022691501

PARD6G	-2.140562808	0.022723462

TRUB2	0.887448148	0.022736153

C7orf55	-0.84185277	0.022736153

C12orf5	0.861776662	0.022736153

ACLY	0.659518792	0.022736153

MED13	-2.149342156	0.022744457

ZNF230	-1.110478208	0.022744457

WDR79	1.631929174	0.022744457

KCTD17	0.908031126	0.022744457

LOC651453	0.791351578	0.022744457

METRNL	-0.8311744	0.022748991

LOC728653	-0.6702773	0.022792766

LRFN4	0.759327134	0.022844465

IDE	0.869849906	0.022956703

C12orf28	-1.795030848	0.022984141

CPEB4	-1.104849004	0.023021001

ITCH	-0.765198026	0.023021001

C7orf41	-0.975891362	0.023023613

NRP1	0.803326414	0.023023613

MRPL13	0.58065451	0.023023613

DPH3	0.725744526	0.023023613

TMTC4	0.97795166	0.023023613

KCTD6	-0.630000276	0.023029435

LOC100133477	0.61358752	0.023029435

NUDT7	-0.957737228	0.023029435

AP2A1	0.96699714	0.023029435

GBA	0.787996074	0.023029435

FAM50B	-1.013124146	0.023029435

ERLIN1	0.843842916	0.023108445

LOC647150	0.722192588	0.023108445

LOC441506	0.602823836	0.023108445

ARPC1B	1.122757946	0.023108445

LRRC6	1.14767824	0.023108445

YIPF6	-0.682125924	0.023108445

LOC728037	1.047387712	0.023137979

S100A10	1.031111836	0.023137979

TMEM101	0.559879292	0.023137979

CTDSPL	-0.714128662	0.023137979

LARS	0.518354652	0.023143925

PBRM1	0.615506216	0.023176548

LOC642795	-1.103193528	0.023207564

KIF20B	1.30870856	0.02326316

AMY1C	0.656692152	0.023272897

GMPS	0.81663277	0.023272897

ZNF514	-0.870687734	0.023272897

ANKRD52	1.06713338	0.023272897

PALLD	-1.435438988	0.023313856

EIF4A2	-0.615514016	0.023314298

CENPO	2.043648762	0.023314298

LOC338799	-1.155420508	0.023343467

PQLC3	-0.864143978	0.023425556

LOC729595	-0.638470754	0.023425556

CTPS2	-0.634381432	0.023425556

NUAK1	-0.9659464	0.023502232

DNAJA4	-1.448832006	0.023502232

TMEM183A	0.553530918	0.023503897

LOC729646	0.856603622	0.023505526

LOC649679	0.806223188	0.02354189

POLR2D	0.700593474	0.023569645

ARHGEF10L	-1.094676676	0.023569645

JAZF1	-0.825315364	0.023571623

RGS1	2.353257308	0.02358905

GMPPA	0.97800151	0.023619683

THBD	2.41013757	0.023691914

BCL3	1.335769286	0.023691914

LOC648984	1.18025087	0.023691914

IL10RB	0.601081506	0.023705794

UBE2J1	0.974653246	0.023705794

TNFAIP6	2.003009786	0.023705794

PLEKHB1	-1.46678125	0.023705794

LOC645381	-0.586500306	0.023792675

AIFM1	0.577499302	0.023792675

ACTR10	-0.62517425	0.023797821

SH3GLB2	-0.610592272	0.023803591

LOC641768	1.10665117	0.023803591

NBPF1	-0.798407604	0.023803591

LSM2	0.514514802	0.023803591

INSIG2	-0.699918872	0.023815024

KLHL29	-0.589320868	0.023815024

LOC649049	0.860115196	0.023839819

LOC644830	-0.979320978	0.023849926

SEC23B	0.627519544	0.023918806

TK1	3.23820499	0.02395976

SFXN5	1.299192184	0.02395976

MGC3020	-0.932806978	0.02395976

ARMET	0.966436522	0.023963129

C1QTNF6	1.68811236	0.023963129

ZNF248	-0.63714896	0.023963129

SGMS2	1.022572764	0.023963129

MT1F	2.317165186	0.023982904

LOC100132032	0.852766148	0.023996118

TSPYL4	-1.150495028	0.024043497

SVIL	-1.023869972	0.024043497

TBC1D1	-1.058780204	0.024043497

PIR	-0.761638494	0.024079731

GPBP1L1	-0.686942968	0.024105294

BCL2L12	1.02858538	0.024108865

NHS	-1.362824966	0.024144487

FBN1	0.962152616	0.024144487

SPHAR	-1.521108164	0.02414468

RORA	-0.964768184	0.024151567

USP5	0.60890594	0.024172315

C3orf26	1.052838694	0.024182514

RAB6B	-1.204912188	0.024182514

FLJ10081	-0.547583904	0.024182514

LOC644464	0.560811686	0.024182514

GALIG	-0.91357537	0.024182514

LOC642469	0.96725514	0.024190614

CXorf59	0.988428842	0.024224939

LOC729686	0.854860358	0.024256749

TWIST1	0.7760985	0.024262347

UBA1	0.794278896	0.024262347

PTPRS	-1.70501493	0.024305122

CPEB1	-1.460167618	0.024317952

TYMP	2.144908678	0.024317952

LEPREL1	-1.083809448	0.024317952

ZNF644	-0.546370104	0.024317952

FBXW11	-0.506293034	0.024330309

C5orf5	-0.913756368	0.024330309

AIG1	-0.95903916	0.024390642

WDR60	-0.761953134	0.024390642

LOC729446	-0.559605016	0.024411693

DDX17	-1.068080712	0.024411693

FAM158A	0.868820174	0.024411693

SCARNA7	-1.738222642	0.02441612

COL5A3	2.993214454	0.024416793

GBGT1	1.032774846	0.024416793

CNIH4	0.609702416	0.02444375

LOC401093	-2.11767699	0.024462466

IL4R	0.892260932	0.0245078

QTRT1	0.808234188	0.024508142

LIN54	0.600659762	0.024509745

DUSP26	-1.94835023	0.024509745

CAD	1.180105406	0.024517232

ZNF83	-1.06435848	0.024517232

ZFAND1	-1.07352356	0.024528368

CCDC137	0.930093398	0.024559489

CHMP2B	-0.94325406	0.02456605

YAF2	-0.546176328	0.02460106

CD2BP2	0.622229126	0.024602541

FBXO3	-0.836633482	0.024602541

SNX25	-0.709368662	0.024630457

LOC647000	0.989728194	0.024631755

SLC25A13	0.897923238	0.024631755

CXCL1	2.55732446	0.024645878

CYB5R3	-0.651783138	0.024654934

POLDIP2	0.596724366	0.024682597

SEC22C	-0.554564352	0.024682597

FAT3	-2.1480874	0.024682673

PSMD13	0.614331338	0.024682673

ATP8B2	-0.591146516	0.024697776

GNG7	-0.998189054	0.024697776

CDK8	-0.786052204	0.024751525

PSMB6	0.5183108	0.024751525

HINT3	-0.945645548	0.024751525

TRAPPC6B	-0.592686294	0.024828446

EXOSC6	0.580725314	0.024828446

LSM14A	-0.51089498	0.024841783

GPNMB	-1.364093936	0.024841783

ANKLE2	0.503202524	0.024841783

TTPAL	0.713552644	0.024848522

CEP55	3.990596816	0.024848522

ABR	-0.829981734	0.024901627

LOC100132395	-1.224081092	0.02490594

CD276	1.523455934	0.024995144

LOC728661	-0.875587206	0.024995144

C15orf59	-1.04562815	0.024995144

ZNF302	-0.584085554	0.025019623

CRYAB	-1.154753932	0.025019623

KDELC1	1.052527678	0.025033398

RNF38	-0.718943334	0.025033398

RPL22	-0.792436678	0.02504056

NAAA	-0.78751063	0.02504056

TC2N	-2.836137736	0.02520454

LOC100133328	0.76189726	0.025213445

PPIAL4A	0.646486314	0.025213445

NUP88	0.597433688	0.025220953

SGCE	-0.871335274	0.025309514

LOC732300	0.952014824	0.025309514

GLYCTK	1.187164722	0.025309514

AKAP1	-0.939306616	0.025309514

PTGR1	1.028372274	0.025309514

ZFYVE21	-0.855473556	0.025315892

RAVER1	0.685274104	0.025328941

CYTH2	1.393997604	0.025328941

ZMYND11	-0.687319714	0.025328941

MPV17	0.670996546	0.025328941

NR1H3	0.59450881	0.025328941

PHACTR1	-0.993587436	0.025328941

GNPTG	-0.616193538	0.025328941

GPATCH3	0.813022054	0.025328941

ERF	0.769670186	0.025328941

NPIP	-0.646970612	0.025365971

TCF7L1	-1.03541361	0.025365971

SLC6A16	-0.811046314	0.025366555

MSL3L1	0.96695785	0.025366555

KIAA0562	0.9482491	0.025499713

WFDC1	-2.267699592	0.025575278

UACA	-1.24885105	0.025583418

FSTL3	-0.804397104	0.025594168

RNASEH2B	0.732184506	0.0256012

ZNF350	-1.179002796	0.0256012

TM7SF2	-1.189901548	0.025619085

TEK	-2.073497878	0.025661506

ZBTB32	1.28947712	0.02566539

MAP4K4	0.865906472	0.02566539

ATL2	-0.739197142	0.025693854

LOC650646	0.94852082	0.025725275

YTHDC2	-0.730014244	0.02576369

DGKA	0.746600382	0.025799161

ERP44	-0.55608745	0.025799161

LOC402644	1.11756201	0.025807247

RALGAPA1	-0.78550777	0.025823742

GSK3B	0.976392056	0.025824566

SHROOM3	-1.74004554	0.025870145

FLJ39653	-0.815695918	0.025870776

MT1M	1.707392736	0.025902522

BAGE3	-1.359571424	0.025927053

ZBED5	-0.685146368	0.02595605

FHL3	0.882510736	0.026029841

COL4A4	-1.812427382	0.026029841

EPM2A	-1.351265986	0.026034968

SSPO	-1.123282026	0.026034968

RHBDD3	1.323949132	0.026044247

LOC649999	-1.155328716	0.026088203

ANKFY1	-0.678974626	0.026117656

SYNGR1	-1.196427134	0.026117656

MLLT10	-0.787559808	0.026168843

C3orf17	-1.030636468	0.026183405

LOC643995	1.221358024	0.026183405

LOC285741	-0.649539632	0.026219645

RNASE4	-1.130566062	0.026231592

BCAP31	0.626424816	0.026237803

EIF2B3	0.723596072	0.026237803

COL3A1	1.303485344	0.02625325

PPP1CB	-0.92835368	0.02625325

KIDINS220	-0.757237368	0.02625325

FAM136A	0.59832948	0.026268629

MICALL1	1.291132874	0.026304675

MSC	1.449786788	0.02634097

ZNF471	-1.15172617	0.02634097

TARS	0.538716432	0.026455184

SLTM	-0.52227299	0.026475912

ZBTB42	0.72907872	0.026527411

SLC40A1	-1.390378682	0.026550569

ZNF259	0.749307376	0.026550569

C20orf24	0.630086838	0.026558628

KIAA0240	-0.81224475	0.026558628

MAK16	0.66253722	0.026590881

SLC6A9	1.642133374	0.026604314

MYBBP1A	1.210596528	0.026621014

RIN1	0.838078472	0.026621014

MITF	-1.112318714	0.026659548

IKZF5	-0.790243914	0.026669328

SSR1	0.662341372	0.02669584

RAB5B	-0.51696731	0.026702833

SLC35A4	0.530376346	0.026705803

RFWD3	0.812815348	0.026721188

LOC728368	0.530106842	0.026743727

LOC652881	-1.140549412	0.026777212

SNRPB	0.69196697	0.026778896

CCL16	-1.837056012	0.026790154

PPFIA2	-1.902742852	0.026823486

C18orf1	-2.37324427	0.026823486

PHF1	-0.784031732	0.026823486

LOC729970	-1.396234368	0.026823486

LOC100128252	-0.894040562	0.02683551

LOC643293	-0.963412516	0.02685213

CDR2L	0.64813192	0.026854596

PAQR7	0.801689356	0.026922335

IRS2	-1.255435388	0.02704464

LOC649853	1.163895226	0.027068724

MUC1	1.146676018	0.027068724

KCNN4	1.082096936	0.027068724

LOC388707	0.65072338	0.027068724

LOC387703	0.607862336	0.027068724

TM9SF1	0.591234224	0.027068724

FIT1	1.258301712	0.0271262

ZNF706	-0.747973718	0.0271262

DNAJC7	0.624527374	0.027143855

C10orf2	1.340307504	0.027156765

COL18A1	-0.965196192	0.02716719

TUBB	0.83530413	0.027196096

CCDC15	0.99849387	0.027196096

FAM80B	-1.137942594	0.027227854

LOC645349	1.362711392	0.027254886

FAP	1.86902345	0.027284072

CX3CR1	-1.586657092	0.027284072

PSMD8	0.667265614	0.027290735

DCPS	0.768989806	0.027290735

GSTM3	-1.507007618	0.027301787

HM13	0.610016158	0.027301787

FRAS1	-1.250778188	0.027311587

LOC641996	1.100751522	0.027312859

TPMT	0.856232408	0.02732548

EXOD1	1.885152826	0.02732548

TMEM55A	-1.059113374	0.027339688

TPST1	0.675805718	0.027339688

SLC25A39	1.197661104	0.027339688

PLSCR3	0.534513512	0.02736736

TPST2	0.848010528	0.02736736

STK10	1.18032077	0.027379678

SND1	0.914388786	0.027390313

CEP152	0.875153752	0.027390313

IRAK3	0.893929848	0.027408013

SFXN1	1.342937132	0.027424867

EPHA4	-1.254207674	0.027424867

KCNA5	-2.71627201	0.027424867

XRCC3	1.101386966	0.027460412

KBTBD9	-0.675786894	0.027472892

LOC730994	-0.877593716	0.027506932

HSPA4L	-1.365398692	0.027557673

C21orf63	-0.993212202	0.027561051

RARRES2	-1.486479366	0.027583481

TTL	0.794316922	0.027625406

VRK1	0.984273718	0.027650603

KRTCAP2	0.515121158	0.027650603

WDR68	1.084957544	0.027650603

ROBO3	-1.571997576	0.027710534

PLD6	-1.038011066	0.027710534

PC	1.72565982	0.027711817

FLJ12355	-1.009517522	0.027762308

NFKB2	1.027542226	0.027762308

MTX1	0.851855328	0.027762308

GMDS	-0.781213562	0.027764144

NRGN	1.206666538	0.027781428

SASH1	-0.526807262	0.027781428

PCDHB10	-1.390842384	0.027823846

PLD3	-0.639040732	0.027823846

LOC653375	0.68163732	0.027834147

LOC644990	-1.504977572	0.027838624

ARHGAP15	-1.940888924	0.027838624

MPDZ	-0.971754472	0.027838624

PRC1	2.297807336	0.027860219

NBEA	-1.394089444	0.027911866

TMEM97	0.54271235	0.027911866

ACAP2	-0.550368872	0.027911866

TRAM2	0.629577552	0.027911866

SLAIN1	-1.777442152	0.027957317

LOC729816	1.769568374	0.027957317

C14orf93	0.620426822	0.027957317

PPP1CA	0.741030138	0.027957317

WDSUB1	-1.20714754	0.027957317

LIFR	-1.467762164	0.027957317

STRAP	0.59858479	0.027982319

C1orf133	-1.801087476	0.027982319

RALGAPB	0.570197562	0.027982319

C9orf86	0.804854724	0.027982319

HRSP12	0.634358396	0.027982319

COL1A1	2.038369438	0.028036484

LOC389141	0.58143179	0.028044001

SLC17A9	1.605876342	0.028063664

NEIL1	-0.955099126	0.028124802

GSS	0.832373706	0.028124802

TMEM14D	-0.555193764	0.028124802

PRR13	0.527745578	0.028144406

SNORD68	0.889465836	0.028146704

LOC732425	1.038012988	0.028192774

DUSP28	-0.65278625	0.028225459

TRIP12	0.51382915	0.028225459

RHOG	0.819218492	0.028237363

LOC651849	1.14891865	0.028295769

TCP11L1	0.634874264	0.02830493

HDAC5	-1.606750118	0.028328933

CLN6	0.939454192	0.028328933

C21orf34	-1.576369802	0.028328933

FLAD1	0.733368046	0.028526702

CSNK1G1	0.69237218	0.028530455

TEX10	0.583007552	0.028532827

ProSAPiP1	-1.184504622	0.028532827

UBE2E2	-0.519994484	0.028532827

LIPT1	-1.015817732	0.028608163

SS18L2	0.545138232	0.028640441

OGT	-0.764899086	0.028640441

RAB2B	-0.640182172	0.028640796

LOC388524	0.500625976	0.028670889

LOC100132673	0.852469626	0.028701826

MTHFD1L	1.005695918	0.028720114

MRPS33	0.635374604	0.028720114

LOC653157	-1.119980496	0.028730611

CA4	-2.301578338	0.028780697

LOC652968	-0.671520836	0.028780697

CKAP2	1.232613452	0.028838959

GARNL3	-1.328165778	0.028841823

PXMP4	-0.7373443	0.028841823

ZNF573	-1.164703854	0.028843467

SLC2A12	-1.71254606	0.028860291

TIMM8B	0.508366658	0.028870379

EP300	-0.615251304	0.028881647

CCDC136	-0.943137146	0.028911113

SLC7A1	1.059115008	0.028931675

WDR27	-0.729415364	0.028942323

MPZL1	0.606886342	0.028942323

CHCHD3	0.674222964	0.028942323

SDPR	-2.838855322	0.028947696

PLS1	-1.592921674	0.028952012

MEMO1	0.635656052	0.028998211

RPS26P11	0.854778546	0.029001913

FAM59A	-1.525082822	0.029050111

ZNF251	-0.76467666	0.029050111

C9orf127	-1.209329244	0.029082772

TMEM106B	-1.139349946	0.029113865

DHX32	0.601660638	0.029134785

NAV1	0.756489724	0.029160724

LOC402342	1.719688874	0.029216917

SORBS3	-0.607675272	0.029224196

LOC100129201	0.62929076	0.029250899

NCL	0.637421586	0.029250899

LOC650321	0.96016426	0.029271347

TM2D3	-0.555954822	0.029317969

TMEM170B	-0.84766219	0.029398633

BAZ2B	-0.577318122	0.029398633

TRNAU1AP	-0.769415042	0.029398633

C19orf47	1.562598328	0.029398633

RAF1	-0.531142332	0.029412799

LOC654074	0.814923356	0.029415247

TSPAN8	-3.002241698	0.029442667

TRAF3IP2	1.254228606	0.029504092

RHOU	-1.008310214	0.029509565

SGCB	-0.95251686	0.029513204

ENG	0.904816916	0.029513204

ZDHHC11	-1.027241184	0.029525603

NKTR	-0.835620734	0.029542258

RFXANK	0.764197408	0.029689265

MOSPD3	0.500588328	0.029709256

MEOX2	-2.368625448	0.029745393

LOC92497	-2.022306042	0.029745393

SDCCAG3	0.657746184	0.029817551

UBE2W	-0.823600176	0.029817551

LOC441481	0.810934506	0.029817551

JMY	-1.293480596	0.029838607

LOC642156	1.059001944	0.029838607

S100A8	4.23578416	0.029838607

ARID5B	-0.71141623	0.029853282

DHX34	0.608973452	0.0298699

C15orf29	-0.700496204	0.030003037

ZNF643	1.10093951	0.030003037

BUD31	0.501596192	0.030038578

P4HB	0.853614002	0.030099747

C1orf162	1.318343956	0.030124011

CASKIN2	-2.41190541	0.030125617

ALDH1B1	-1.959436814	0.030139849

HAVCR2	1.446160868	0.030182474

LOC642361	-0.636059172	0.03018677

TncRNA	1.231844508	0.03018677

BTAF1	-0.524886542	0.03021328

ARPC4	0.708993398	0.030266604

NDUFA10	1.089349748	0.030314271

NCOA1	-0.693335852	0.030318119

CACNA1C	-1.055182034	0.030336238

CHD6	-0.751352904	0.030382384

TP53BP1	0.668214434	0.030394047

LOC653505	0.801616088	0.030515783

PLXNB1	-1.291226094	0.030515783

TMEM50B	-0.57794086	0.030569941

RAB11FIP2	-0.721050826	0.030569941

RILPL1	-0.831478012	0.030569941

CTNNBIP1	-0.710700168	0.030569941

AMN1	-1.483203976	0.030569941

LOC729708	2.65906953	0.030569941

VCL	-0.734980682	0.030582686

SLC25A42	-0.622551836	0.030582776

LOC728193	1.562458072	0.030585387

NDUFB2	0.507923668	0.030614799

TEAD2	0.64077057	0.030627131

RNPC3	-0.994516158	0.030627131

SOX13	-0.8692138	0.030643258

SMC2	0.60183989	0.030661909

ZNF788	0.72847206	0.030687695

RPS6KA5	-0.76869824	0.030689492

SLC20A2	-1.039283036	0.030703016

SLC25A11	0.717112994	0.030828623

CHD3	0.980861634	0.030828623

NOC3L	0.605596206	0.030828623

CCDC74A	1.648443076	0.030828623

FAT1	-0.829831388	0.030829867

FRMD4B	1.591168754	0.03086586

ESRRA	0.928377878	0.030928453

LOC91461	-1.329162786	0.030928453

SOSTDC1	-1.099158636	0.030950395

CHIC2	0.693713416	0.030965011

VAPA	-0.667170082	0.030977026

BTN2A1	-0.538317896	0.030977026

ASAP2	-0.834766408	0.03097845

LOC389168	0.502663428	0.03097845

FAM107B	-1.088998546	0.030988402

SCRN1	-0.785117842	0.031039632

C20orf20	0.755769156	0.031043161

LOC647588	-1.176690644	0.031055475

TMEM189-UBE2V1	0.67310039	0.031083254

NAMPT	2.273998162	0.031113613

TCFL5	-0.663297028	0.031113613

BCAT1	1.69035554	0.031119784

KLHL3	-1.15646712	0.031140629

MRPS28	0.721210704	0.031169287

GPN1	0.502033572	0.031187247

LOC100130131	1.811928178	0.031187247

SNORA57	1.2072987	0.031187247

KIAA0692	1.06603459	0.031187247

ATP5B	0.53032781	0.031187247

NT5C2	-0.563897512	0.031187247

KIAA1683	-1.635798004	0.031187247

LOC730382	0.680755648	0.031218941

LOC389904	1.111330026	0.031268276

GBP4	-1.702478362	0.031268276

LOC407835	0.7848344	0.031268276

CD300LG	-3.083202696	0.031278364

ELMOD2	0.71629197	0.031278364

LOC730092	-0.72008715	0.03137829

ANKRD26	0.768970822	0.031381069

LOC728126	0.571780674	0.031383716

ATP6V0C	0.650217686	0.031384898

LOC440737	0.501122606	0.031391612

ANKRD57	-0.681945396	0.031391612

ATP7B	-0.56787469	0.031391612

ID2	-1.59646967	0.031391612

ACTL6A	0.567203864	0.031406699

AFG3L2	0.535913114	0.031482136

PNMA3	-2.691850534	0.031482136

IFIT1	-1.25466957	0.031495509

C2orf64	-0.772681126	0.031505739

TSPAN12	-1.514386448	0.031516129

USP15	-0.530557336	0.031516129

PI4K2A	0.510974264	0.031578166

RAB38	1.053236598	0.031589133

2-Mar	-0.659518704	0.031744944

LOC100134711	-0.9342036	0.031826614

FTSJ2	0.617527866	0.031881011

CYTL1	-2.296851014	0.031881011

CCNDBP1	-0.871993344	0.031898769

IARS	0.53482357	0.031901877

PKDCC	-1.062265814	0.031910602

LOC100129697	-1.539024726	0.031928414

CYTH3	-0.92886812	0.031936108

HEG1	-0.800623142	0.03196881

ZNF512	-0.793824258	0.032092048

SLC25A29	-0.754046404	0.032092048

ASAH1	-0.867585262	0.032128477

HLA-DPA1	-1.686033822	0.032128477

PHIP	-0.599155706	0.032137944

ADAMTSL3	-2.001234866	0.032142267

HYI	-1.269657222	0.032142267

PARP3	0.713799516	0.032181153

TMEM45A	0.868445224	0.032227342

TM7SF3	-0.635540204	0.032261415

CTPS	-0.684032648	0.032269901

C1orf107	0.792402968	0.032369583

ZFAND2B	-0.658487516	0.032426498

SHISA3	-1.300320962	0.032442844

LOC339843	-0.758978332	0.032485913

SMARCA2	-0.660870902	0.032492803

UNC50	-0.59315302	0.032492803

SGTA	0.702505518	0.032492803

ZYX	-0.596332038	0.032492803

A2M	-0.973687778	0.032492803

REEP5	-0.742375982	0.032515755

SLC26A10	-1.21694099	0.032528829

TMEM63B	1.05703464	0.032620962

GTPBP4	0.677780886	0.032620962

EIF4EBP1	1.40514774	0.032620962

LOC652595	0.80699362	0.032620962

SVEP1	-1.975372052	0.032630909

ZRANB2	-0.699271746	0.032636464

BHMT2	-1.112146164	0.032643999

FLJ23584	0.664348566	0.032643999

E2F2	3.233346132	0.032647915

MEIS1	-0.933758286	0.032647915

MED16	0.611028424	0.032758195

ZSCAN18	-0.8063571	0.032758195

LOC439953	0.61713983	0.032758195

IQCC	0.65989078	0.032774877

ACACB	-1.417302794	0.032788869

KCNK17	-3.111961888	0.032788869

LOC646750	1.087042904	0.032870483

SNRPA	0.553759874	0.032895765

AVPI1	-0.589845476	0.033041062

RNY4	-1.146690174	0.033041708

SMARCD2	0.988618034	0.033059584

FASN	1.51392681	0.033116784

LXN	1.837745994	0.033116784

ILVBL	0.625953372	0.033138078

COL4A5	-1.51106528	0.033178126

FAM75B	1.001982218	0.033180596

MED25	0.553964092	0.033315801

PPP1R9B	1.52572132	0.033315801

PIP4K2C	-0.689425596	0.033358855

RNF13	-0.653247882	0.033411464

ABCA3	-1.417726746	0.033459719

PAR5	-1.370357522	0.033459719

IFI30	2.055624334	0.033476051

PPAN	1.00403274	0.033633903

RPL23A	0.661650292	0.033662733

B4GALT7	0.70256527	0.033715032

LOC728006	0.564458116	0.033731813

TMEM93	0.511840336	0.033739849

TMEM185B	0.6650191	0.033836845

ZNF84	-0.687013056	0.033873558

LOC100130230	-1.193577784	0.033879267

DEF6	1.196389012	0.033906987

PRMT1	0.847189762	0.033919637

LOC100131261	-0.558122724	0.033948123

LOC644128	-0.77434284	0.033948123

LOC646316	-0.746037848	0.033948123

ZNF533	-2.316114832	0.033973137

ZNF668	0.64837141	0.033977456

MLL	-0.65619506	0.034039718

SC65	1.301590214	0.034075427

NOMO1	0.66066636	0.034143574

NR2C2AP	0.823785778	0.03414416

DSCAM	-0.95120811	0.034181638

FLOT1	0.692516984	0.034181638

PCNX	0.64413408	0.034181638

RNF149	0.703400866	0.034208883

EIF4A3	0.628240588	0.034208883

TEDDM1	1.852735206	0.034282232

PTGDS	-2.803915724	0.034282232

LOC646821	0.725504502	0.034291747

ERBB2	-0.7813455	0.034302668

EIF5A2	-0.788353172	0.034302668

ASCC2	0.577089414	0.034319331

LOC100132139	0.919443576	0.034363671

C7orf43	0.619143558	0.034368907

FAM190B	-0.874710982	0.034368907

C14orf167	-0.728355388	0.034368907

FLJ12949	0.598212266	0.034368907

PLEKHG4	1.189756558	0.034414379

PIP5K1C	-0.650088842	0.03449634

TUBA1B	0.571770534	0.03449634

LOC645018	0.964300632	0.03449634

TMEM80	-0.76176849	0.034578449

LOC727914	1.490065738	0.034578449

RPS26L	0.910710054	0.034613565

CRTAP	-0.810256618	0.034613565

TOPBP1	0.680444376	0.034613565

PLAU	1.744870714	0.034613565

SCIN	-1.728294266	0.034646768

C6orf162	-1.12738213	0.034660305

IFI35	0.595097622	0.034681055

KARS	0.571776082	0.034696565

TTLL5	0.719851416	0.034737222

LOC100133473	0.882259304	0.034749676

SNORD12C	1.61570283	0.034749676

PRMT2	-0.549091678	0.034749676

CCT6A	0.642824152	0.034769992

ZNF629	0.798515238	0.034879487

STARD13	-0.552651446	0.034986714

NIPA1	-0.501820462	0.034986714

TNFSF9	1.06222206	0.034986714

NIPAL1	1.756799418	0.035011456

TIGA1	-0.821344914	0.035045435

COL14A1	-1.698570018	0.035045435

TAGLN	-0.705796694	0.035111735

HNRNPL	0.756588532	0.035111735

EFHD2	0.952114106	0.035111735

C19orf39	-1.266443396	0.035111735

SOD3	-1.332449298	0.035175642

PABPC3	0.86114744	0.035265017

SH3PXD2B	0.916833808	0.035332782

PRKG1	-2.040414496	0.035378441

KIAA1737	-0.634966764	0.035393797

DYRK2	-0.545841908	0.035393797

USH1G	0.799597862	0.035431921

SNORA70C	-1.282756532	0.035487289

C16orf81	1.077394056	0.035493256

CEBPG	-0.620692518	0.035555619

FBXW9	0.876730672	0.03556223

SLAIN2	-0.950151452	0.03556223

SMC4	0.74556423	0.03556223

CCL15	-3.997102574	0.035572214

FAM86C	0.861554084	0.035573067

IQCG	0.615569886	0.035639554

GOLGA6B	-1.126169406	0.0356709

LOC648099	0.64924642	0.035727168

POMGNT1	0.588192444	0.035862633

NUTF2	0.859440566	0.035862633

MBP	-0.862612998	0.035862813

LOC100130932	0.683544886	0.035862813

ANXA10	-1.231274698	0.035862813

ADCY5	-1.925308634	0.035935493

HMGCR	0.567933186	0.035935493

BEND3	0.86203368	0.035986202

ARID2	-0.667680892	0.036029369

TCEAL4	-0.807709758	0.036029369

DOK5	1.282511742	0.036029369

KIAA0831	-0.630448904	0.036037402

LOC442535	-1.73547521	0.036139908

LOC387820	-0.600591784	0.036198322

PSMD4	0.540304754	0.036202957

GIMAP7	-2.31474342	0.036265484

NLRP12	1.421952872	0.036268006

LOC729535	0.935384238	0.036278283

SIDT2	0.744999272	0.036364786

MLYCD	-0.844612256	0.036423807

PHOSPHO2	-0.75992351	0.036424088

SLC37A3	-0.509118994	0.036433306

INCENP	1.628064192	0.036592047

OPTN	-1.008329586	0.036592047

ZNF346	-1.160220768	0.036602007

TWIST2	0.740408954	0.036624284

MGC72080	0.843893136	0.036663857

SLC25A5	0.61040848	0.0367115

RNF216L	0.594970312	0.036803319

AMOT	-1.047139744	0.036883243

MKI67IP	0.641829316	0.036941589

C13orf1	-0.759602634	0.037100371

PIP4K2A	-0.586287964	0.037152824

SNORD16	1.200598564	0.037152824

FLJ42875	-1.832427692	0.037152824

CSPP1	-0.553224846	0.037152824

LOC653324	1.131697338	0.037152824

LOC440978	-1.65173709	0.037166704

DFNA5	-0.673077948	0.037218639

LOC642528	1.133698708	0.037218639

RBM15	0.515892586	0.03723064

AK2	0.63101066	0.03740125

LSM7	0.537830842	0.037423233

REXO2	0.538674486	0.037431104

UVRAG	-0.60180455	0.037496253

ATXN1	-0.60308916	0.037522748

ST13	-0.740473422	0.037623249

FBXO5	0.84254701	0.037705599

MGC3731	0.629323434	0.037705599

NCRNA00219	-0.8328696	0.037742614

PGRMC1	-0.66540004	0.037744591

LOC729841	1.066917596	0.037745391

NME3	-0.834587234	0.037845836

RNF160	-0.538521326	0.037854458

TUBGCP2	0.70551501	0.037880607

ABCA1	0.971533494	0.037880607

SPOPL	-1.126301018	0.037898162

ABHD2	0.90927291	0.037953729

AP1B1	0.640338952	0.038078401

WDR46	0.697845804	0.03807911

ZNF503	-0.86795553	0.038086996

C12orf45	0.546821838	0.038104288

LRRC45	1.395065074	0.038145281

TICAM2	0.78705823	0.038145281

VGLL4	-0.598100494	0.038149973

SSSCA1	1.156624114	0.038149973

KCTD7	-0.961676932	0.03815644

LOC400304	-1.064560246	0.038178778

ATG9A	0.697561682	0.038178778

ME2	0.734672946	0.038199975

DBNDD2	-0.703688882	0.03827287

FLRT2	-1.61202568	0.03827287

VISA	0.704860686	0.03827287

IMPA2	0.889254808	0.038339985

DNAJC17	0.711561934	0.038362445

UNC5B	1.256260884	0.038471742

GTF2E2	0.673737652	0.038471912

LOC644615	1.431425674	0.038569979

PTBP2	-0.741016062	0.038609448

KCNK4	-0.7345009	0.03861378

SNORA45	0.67402698	0.03861378

MRPL4	0.842448836	0.03861378

FBLN5	-1.161053002	0.038621233

SPIN1	-0.957540776	0.038628525

SPC24	1.750668276	0.038668351

ZZEF1	-0.536210066	0.038668847

KIAA1712	-0.594140556	0.038668847

GRINA	0.70918187	0.038745131

FTHL8	0.89741876	0.038754827

MT1G	3.367840484	0.038805331

ETV3	-0.542320356	0.038840635

LOC645659	-1.44668873	0.038840635

PTDSS1	0.541342944	0.038850313

C1orf115	-1.19373888	0.038922336

TGM2	1.242724678	0.038922336

C1orf149	-0.918772428	0.038959123

SLC41A3	-0.727418386	0.039017518

C20orf134	1.930723736	0.039017518

KIAA1128	-0.784746208	0.039017518

ALMS1P	0.957755336	0.039017518

GOT1	0.569621898	0.039017518

TMEM44	0.83725276	0.039017518

C21orf126	0.690149332	0.039017518

LCAT	-1.088739878	0.039017518

DPH5	0.532918024	0.039084523

ATP9A	-0.88595461	0.039134551

C3orf19	-0.523336256	0.039231529

VN1R2	0.73140485	0.039231529

GGH	1.420279846	0.039231529

LTBR	0.765062724	0.039231529

SMURF1	-0.750098504	0.039318546

GUCA2A	1.517756692	0.039340313

JAM2	-1.141450002	0.039349603

ZNF805	-0.612922628	0.039353922

NINJ1	-0.668405656	0.039353922

NBPF11	-0.524634772	0.039353922

HPS1	0.776613206	0.039353922

PIP5K2A	1.209969672	0.039378971

TASP1	-1.407055026	0.039476629

RPL23AP53	-0.738397348	0.039482776

LOC653344	0.95676857	0.039482776

LOC648057	-0.790841696	0.039482776

IER3	1.593284024	0.039584398

HAAO	-0.514298768	0.039617239

GPRC5A	-2.263531582	0.039617239

VKORC1L1	0.884607174	0.039665104

MRPS16	0.730522748	0.039665104

GATS	-0.907272634	0.039665104

LOC645652	1.371101288	0.039665104

CPXM2	-1.166128006	0.039670476

TOM1	0.528452962	0.039671693

TUBB4Q	0.940560832	0.039700758

LOC100133609	1.554148632	0.039724883

TUBB2C	0.940485946	0.039727642

MAP2K2	0.819578364	0.039727642

PRPH	-1.664183848	0.039819998

FNBP1L	-1.016759658	0.039826345

C9orf106	1.099524724	0.039826345

KLRAQ1	-0.673132428	0.039848975

CROCCL2	-1.285006442	0.039894095

HPS5	-0.743222024	0.039901553

LOC729764	-0.704381932	0.039901553

LOC650215	0.574351976	0.039901553

PEG3	-0.826422058	0.03998801

LOC728312	1.2916603	0.03998801

ZCCHC8	-0.556042144	0.03998801

USP47	-1.356303502	0.039999436

SP3	-0.58279143	0.039999866

ZNF385B	-1.418710488	0.039999866

ESRRAP2	0.547953496	0.039999866

DOCK11	-0.731157026	0.040043873

LOC651816	1.280821746	0.040056227

SMAD4	-0.506756216	0.040056227

KIAA0194	0.508536414	0.040056227

LOC100129960	-1.310668776	0.040066064

ZC3HAV1	0.561133694	0.040066064

LOC649851	1.366407646	0.040066064

FRMD8	0.891623338	0.040082218

ANLN	3.366828948	0.040155914

SCARF2	-0.822047286	0.040157229

HNRPDL	-0.556990024	0.040157229

MYO10	1.073698502	0.040237289

MT1X	2.323465236	0.040237289

LOC728602	0.58070435	0.040237289

RBPMS2	-0.99800754	0.040237289

LOC440063	1.097671096	0.040237289

MFGE8	-0.916863796	0.040359967

FAM38A	0.928590494	0.040473193

COASY	0.606108798	0.040473193

MFHAS1	0.852909026	0.040473193

FLJ20718	0.71365526	0.040475535

LOC728937	0.730334488	0.040521492

MPHOSPH6	0.69280502	0.040570553

TACC3	2.235039026	0.040570553

LOC642325	-1.197887964	0.040604944

PHACTR2	-0.599893576	0.040604944

CYP39A1	-1.39203882	0.040651017

GLT25D1	1.05666162	0.040711779

LOC90120	0.577066988	0.040723029

KIAA0232	-0.904122314	0.040723029

LOC653125	-1.103027224	0.040775203

FUCA1	-1.1114756	0.040801301

COTL1	1.59306758	0.040852642

FAM174A	-0.838345566	0.040878646

DAAM1	-1.036065136	0.040901773

ZNF282	0.667179092	0.040937462

LOC643997	1.04632747	0.040946926

ATRX	-0.739965844	0.041002451

CKMT2	-1.704538464	0.041002451

ALDH5A1	-1.007201014	0.041002451

CD34	-2.416122688	0.041017728

HLA-DOB	-1.325840974	0.041017728

TXNDC5	0.63035222	0.041017728

IFNAR1	0.638463332	0.041020174

LOC100131360	0.639553738	0.041020174

BBX	-0.645492094	0.041120702

COL5A2	1.284572668	0.041120702

DENND5B	-1.336555314	0.041120702

WDR38	1.079221048	0.041120702

RAMP3	-3.502597542	0.041181969

LOC731196	-0.824619056	0.041223569

LAT	-0.887606708	0.041247892

CD2AP	-0.886115832	0.041262873

C21orf56	0.649706974	0.04127786

TMEM77	-0.606649526	0.04127786

ERRFI1	0.81660545	0.041327961

PPBP	2.198904514	0.041553734

MIER1	-0.772851668	0.041559413

PKHD1L1	-1.620857578	0.041559413

LOC643319	0.795304862	0.041668161

ZCCHC11	-0.509234282	0.04168237

GNRH1	-0.848708174	0.04168237

LOC728031	0.541815642	0.041772801

PFN1	0.513902902	0.041772801

PCMTD2	-0.593343072	0.041772801

COL13A1	-1.65225579	0.041772801

LOC388681	-1.168732098	0.041772801

SLC35F1	-3.029409082	0.041853359

TOMM20	-0.588162272	0.042012595

IFRD1	-0.644650074	0.042057977

RBM12B	-1.08793947	0.042057977

CWF19L2	-0.715146662	0.042068513

NLN	1.007969386	0.042072174

FKBP9L	-0.521445444	0.042072174

LOC647568	1.048814158	0.042135959

TMTC1	-1.152436976	0.042135959

RBMS1	0.634219224	0.042135959

SERTAD1	0.804058644	0.042166847

MAP1B	-0.948843796	0.042169133

CD300A	1.895621918	0.042261703

BAIAP2L2	1.739697486	0.042277665

ZNF266	-0.616434726	0.04235989

KEAP1	0.61939676	0.042411644

ADPRH	1.027247264	0.042416522

CCM2	0.631352432	0.042416522

LOH3CR2A	-1.24015827	0.042482901

OMA1	-0.702747748	0.042482901

MANSC1	-0.762494056	0.042538961

PEX11G	0.867572122	0.042538961

CCT3	0.578317746	0.042588125

CD97	-0.797596946	0.042593234

DDIT4L	0.82469542	0.04262682

LOC388564	-0.53818763	0.04262682

AFTPH	-0.514376266	0.042629111

LOC652864	0.70926144	0.042632581

PDGFRB	0.695901332	0.04267454

VDR	1.005054056	0.042761081

GOLPH4	-0.52251543	0.042939378

VPS4B	-0.525214458	0.042951834

WASL	-0.591278608	0.042951834

LOC728047	-2.77801673	0.042951834

GNG5	0.542370498	0.042951834

GATAD2B	-0.689504694	0.042951834

LOC399491	-0.577566778	0.042978531

CCDC151	1.519923328	0.043013473

GUF1	1.03380543	0.043019776

ZNF695	1.50671616	0.043081848

BCCIP	0.582233542	0.043081848

KIAA1602	0.75492606	0.043127829

C10orf104	-1.33467533	0.043128837

HTR2B	-2.088706038	0.043293122

ZNF317	0.603290376	0.04330156

SGIP1	-0.816216902	0.043340525

GOSR1	-0.685384118	0.043340871

AIF1L	-2.2717341	0.043355204

C16orf88	1.146254428	0.043395963

ADRB2	-0.714646838	0.043399721

MRPS14	-0.721656594	0.043479834

SLC25A1	0.62646117	0.043479834

MRPS21	0.522055206	0.043479834

HOXA2	-1.672289784	0.043479834

TMED3	0.8803692	0.043495123

FAM10A7	-0.894534812	0.043534167

PECR	-0.650857968	0.043547575

LOC343184	0.628407702	0.043547575

TMSB10	0.82040335	0.043606582

AGPS	0.9692381	0.043606582

PDE5A	-1.280977326	0.043606582

LOC652115	1.35274766	0.043606582

LOC286512	-0.741138236	0.043637985

TMEM14A	-0.70494464	0.043642732

SPPL2A	0.523210444	0.043642732

NUP85	0.560855504	0.043662767

MARS	0.5804438	0.043662767

CST3	-0.552811026	0.043703579

C6orf168	-1.484252002	0.043703579

C2orf67	-1.06582642	0.04374466

RNF103	-0.656052944	0.04374466

CCDC73	1.353817044	0.043749385

PTPRM	-0.925350876	0.043749385

SELM	-0.79888486	0.04379434

NSBP1	-0.689537336	0.043797257

C1orf53	1.457649428	0.043815133

SEPN1	0.812955566	0.043828974

C19orf25	-0.574419996	0.043862675

PGCP	-0.999818352	0.043874739

TPD52L2	0.507531968	0.043905805

OTUB1	0.591721094	0.043919799

TLE6	2.04413642	0.04398957

MYBL1	-1.320372146	0.044051239

ZNF532	-0.692838614	0.044149574

LOC644517	0.81170687	0.044154276

AIM2	-1.702671384	0.044154288

FCRLB	0.7833849	0.044154335

EIF4E3	-1.029458132	0.044241125

LOC729057	0.884904862	0.044264642

TBL1XR1	-0.741824586	0.044276583

PPDPF	0.818392498	0.044277772

SAAL1	0.54987434	0.044277772

C2orf40	-2.080821952	0.044293389

HMP19	0.934515448	0.044309829

LOC201229	-1.233982592	0.044324803

CCDC135	0.798874794	0.044330505

ZC3H7A	-0.522465406	0.044380173

ZNF540	-1.732020944	0.044437083

TOLLIP	-0.596788576	0.044437083

LOC647080	0.633874844	0.044437083

POLD1	1.34171029	0.044437083

TGIF1	0.701135084	0.044437083

REEP4	0.95401491	0.044459755

SMPD2	0.960561876	0.044537544

BNIP3L	-0.58055712	0.044564229

ZC3H12A	1.536313622	0.044628601

ANKRD46	-1.224373144	0.044693914

C17orf41	1.053691038	0.044844707

TRPV2	1.098623044	0.044899968

C9orf150	-1.297755676	0.04498719

CCDC21	0.776658278	0.045018507

LOC644934	0.72258946	0.045018507

SRI	-0.686350784	0.045061445

C16orf7	0.885164852	0.045066379

MYLK4	-1.120708788	0.045097473

SERTAD4	-1.89925722	0.045097473

MPP5	-0.59223013	0.045097473

ZNF615	-0.748074544	0.045102789

CALCOCO2	-0.848183474	0.045200444

POLR2J	1.171866416	0.045200444

FAM64A	2.310806098	0.045231617

HS2ST1	0.888265098	0.045231617

WBSCR22	0.560210808	0.045254715

KIAA0146	0.56500253	0.045295976

MECP2	-0.601321318	0.045295976

TSPO	0.54234279	0.045316005

STAG3L2	-0.788541554	0.045349505

HMHA1	-0.909568176	0.045349505

LOC651575	0.723134068	0.045363067

APRT	0.768725178	0.045363067

CPB1	-2.891028138	0.045406674

WASPIP	-0.688966788	0.04542731

ZC3H12C	-0.648300318	0.045454462

CPS1	0.557445754	0.045473355

C20orf55	0.605715274	0.045479815

STK16	1.035515302	0.045530086

ITPR1	-1.237758614	0.045530086

FAM50A	0.539147774	0.045597728

ZNF418	-1.169931256	0.045601412

GJC2	-2.284207582	0.045601412

GRPEL2	0.656835812	0.045632409

VPS25	0.536318902	0.045644935

LOC388796	0.679655382	0.045697645

PIP5K1B	-0.878862464	0.04569993

ARFGEF2	1.032738602	0.045737611

NFKBIE	1.369079512	0.045787385

SCAPER	-1.131724532	0.045826118

ZNF365	-1.689834284	0.045828234

RPL15	-0.603163114	0.045828234

LOC440928	-0.88895429	0.045859958

SH2B2	1.203745866	0.045860329

PMS1	-0.670887024	0.045876527

LOC440900	1.2672847	0.045972992

TACC2	-0.779595954	0.046189563

DDEF2	-0.844038088	0.046293484

ST7	-0.573574866	0.046374557

RPAP1	0.614795054	0.046403864

ELF1	-0.663946352	0.04642615

LANCL1	-0.539577218	0.046433503

POLE3	0.584205752	0.046433503

PFKFB4	0.92020333	0.046433503

LOC728802	-1.496151636	0.046474085

SOCS3	1.861531352	0.046489725

COQ7	-0.774433038	0.046489725

GALNT12	-0.984766716	0.046489725

DNAJC21	-0.771818524	0.046493441

MYST4	-0.93918812	0.046504041

FAM110B	-0.575902416	0.046534583

DDAH2	-0.677393006	0.046563553

ARL1	-0.592180194	0.046602634

MKX	-2.872448884	0.046653717

SDHD	-0.561529508	0.046929228

LOC390671	-0.886643426	0.046971309

NOD1	1.154460036	0.047000417

DLG4	1.086920874	0.04700374

UPF3A	-0.600304376	0.047106286

SNRPN	-0.976474264	0.047193404

ETFB	0.596619484	0.047193404

C13orf33	1.106589146	0.047244424

LOC442582	-0.672613088	0.047249479

POLS	-0.509614612	0.047331967

AZIN1	-0.580088944	0.047331967

FAM46A	0.757039784	0.047368461

SH3BGR	-1.131168848	0.047368461

RND2	-0.933026846	0.047368461

ADAT2	1.06007791	0.047368461

ULK3	1.180182448	0.047368461

PLCL2	-0.886463436	0.04742702

LOC100131971	0.979108252	0.047456904

ZMPSTE24	-0.56356836	0.047507135

SLC38A11	-1.212673754	0.047510567

ATPBD4	-0.734573764	0.047510567

DNM3	-0.847324036	0.047570657

CNDP2	0.698147916	0.047570657

LOC644935	-0.58686192	0.047570657

TRIM4	-0.738699554	0.047570657

CAPN12	-0.767422796	0.047570657

RNF40	0.648031224	0.047651654

SEPW1	-0.757671388	0.047651654

RABGAP1	-0.879636808	0.047657806

FLJ20444	-0.568814952	0.047696861

DNAJB14	-0.63065402	0.047820605

ZNF821	-0.608053374	0.047851289

C11orf10	0.508192722	0.047851289

PIGU	0.557913966	0.048005674

CNTFR	-2.438971432	0.048012459

NMNAT3	-1.060793666	0.048018081

RTP4	-1.061775776	0.048018081

FKBP10	1.609208314	0.048018081

IFIT5	-1.11747269	0.048145668

YIF1A	0.654145788	0.048237225

NOMO2	0.576678846	0.048256228

SNORD35B	1.09323613	0.048274389

EIF4G3	0.694963804	0.048274389

SNORD52	0.874279492	0.048305118

GLRX2	0.659244014	0.048380777

OGN	-1.959831424	0.048464673

LOC340260	1.070848404	0.048503191

POLR3K	0.70349299	0.048530416

APEG1	-1.113203498	0.048648243

LOC648609	1.027714632	0.048657519

VPS36	-0.751877554	0.048657519

SNX16	-0.70854446	0.048682305

SASH3	1.562293666	0.048781214

WDR47	-0.519346472	0.048858218

PIK3R1	-0.930515614	0.048858218

IER5L	0.939152716	0.048884554

HLA-DRB4	-1.507831498	0.04893526

SPIN3	-0.758486204	0.048956184

KIAA0355	-0.684278566	0.049029574

UBE4B	-0.578920134	0.049029574

CNTNAP1	0.795784484	0.049170628

INMT	-2.021320478	0.049170628

FAM98A	0.644069248	0.049190557

LOC729234	-1.249511602	0.049204377

PTPRO	-1.227452558	0.049231098

YIF1B	1.1800823	0.049264468

LOC653820	1.141579398	0.049389946

CSMD2	1.419501062	0.049447382

RNASEH2C	-0.82636538	0.04947514

C12orf29	-0.801410284	0.04947514

EID1	-1.014671526	0.04947514

SSR2	0.713998996	0.049482341

PARD6A	-1.366756632	0.049482341

CRHBP	-0.82375716	0.049573605

WDR45	0.519806548	0.049604372

LOC647481	1.026658288	0.049650787

PXMP3	-0.587663964	0.049665152

RBPMS	-1.00409885	0.049683018

LOC729799	-1.075460878	0.049706342

ARHGEF19	1.041055208	0.049706342

CSNK2A1	0.536630924	0.049706342

ZNF154	1.145004646	0.049753575

C7	-2.197241178	0.049774429

CADM1	-1.722828786	0.049776466

ITM2B	-0.60424842	0.049897454

SLIT3	-0.9504088	0.049898516

MIR571	-1.090358012	0.049928673

TRA1P2	0.785380116	0.049928673
